# The Use of Machine Learning to Predict Prevalence of Subclinical Mastitis in Dairy Sheep Farms

**DOI:** 10.3390/ani14162295

**Published:** 2024-08-06

**Authors:** Yiannis Kiouvrekis, Natalia G. C. Vasileiou, Eleni I. Katsarou, Daphne T. Lianou, Charalambia K. Michael, Sotiris Zikas, Angeliki I. Katsafadou, Maria V. Bourganou, Dimitra V. Liagka, Dimitris C. Chatzopoulos, George C. Fthenakis

**Affiliations:** 1 Faculty of Public and One Health, University of Thessaly, 43100 Karditsa, Greeceagkatsaf@vet.uth.gr (A.I.K.);; 2 School of Business, University of Nicosia, Nicosia 2417, Cyprus; 3 Faculty of Animal Science, University of Thessaly, 41110 Larissa, Greece; 4 Veterinary Faculty, University of Thessaly, 43100 Karditsa, Greece

**Keywords:** machine learning, mastitis, prediction, sheep, support vector machines

## Abstract

**Simple Summary:**

We developed a computational model by employing machine learning methodologies in order to perform predictions regarding the level of prevalence of mastitis in dairy sheep farms. Data for the development of the model were obtained from the findings of a study performed countrywide in Greece in 111 sheep farms. Unsupervised learning methodology was applied for clustering data into two clusters, one with high and one with low prevalence of subclinical mastitis, and, subsequently, a computational model was obtained by means of Support vector machines. The model was verified by taking into account the findings of two subsequent studies in dairy sheep farms, which were performed independently of the initial study. The overall accuracy of the model for the estimation of the level of prevalence of the infection (<25.0%/≥25.0%) in dairy sheep farms was >96%. The findings of this study indicate that machine learning algorithms can be usefully employed in predicting the level of subclinical mastitis in dairy sheep farms, which will help farmers set up appropriate health management measures for controlling the infection.

**Abstract:**

The objective of the study was to develop a computational model with which predictions regarding the level of prevalence of mastitis in dairy sheep farms could be performed. Data for the construction of the model were obtained from a large Greece-wide field study with 111 farms. Unsupervised learning methodology was applied for clustering data into two clusters based on 18 variables (17 independent variables related to health management practices applied in farms, climatological data at the locations of the farms, and the level of prevalence of subclinical mastitis as the target value). The K-means tool showed the highest significance for the classification of farms into two clusters for the construction of the computational model: median (interquartile range) prevalence of subclinical mastitis among farms was 20.0% (interquartile range: 15.8%) and 30.0% (16.0%) (*p* = 0.002). Supervised learning tools were subsequently used to predict the level of prevalence of the infection: decision trees, k-NN, neural networks, and Support vector machines. For each of these, combinations of hyperparameters were employed; 83 models were produced, and 4150 assessments were made in total. A computational model obtained by means of Support vector machines (kernel: ‘*linear*’, regularization parameter C = 3) was selected. Thereafter, the model was assessed through the results of the prevalence of subclinical mastitis in 373 records from sheep flocks unrelated to the ones employed for the selection of the model; the model was used for evaluation of the correct classification of the data in each of 373 sets, each of which included a test (prediction) subset with one record that referred to the farm under assessment. The median prevalence of the infection in farms classified by the model in each of the two categories was 10.4% (5.5%) and 36.3% (9.7%) (*p* < 0.0001). The overall accuracy of the model for the results presented by the K-means tool was 94.1%; for the estimation of the level of prevalence (<25.0%/≥25.0%) in the farms, it was 96.3%. The findings of this study indicate that machine learning algorithms can be usefully employed in predicting the level of subclinical mastitis in dairy sheep farms. This can facilitate setting up appropriate health management measures for interventions in the farms.

## 1. Introduction

In dairy sheep, mastitis causes significant financial effects due to the reduction in milk yield, the downgrading of milk quality, and the rejection of milk after antibiotic administration. Subclinical mastitis is the most frequent disorder contributing to the ‘milk-drop syndrome in ewes’ (in >85% of such cases), a syndrome defined at the flock level and characterized by the reduced milk yield of ewes [1]. Moreover, mastitis has been considered to be an important welfare problem in sheep flocks [2,3].

Machine learning, which is a subfield of artificial intelligence, focuses on the development of algorithmic models that can enable learning and improving performance on specific tasks without explicit programming. In machine learning, an algorithm can be trained based on a particular dataset to recognize patterns and to make predictions or decisions based on that dataset. The two main types of machine learning refer to ‘supervised learning’ (an approach where the algorithm is trained on a labeled dataset with each input paired to the correct output, with the algorithm learning to correspond inputs to outputs and to make predictions or decisions based on new, previously unseen data) and ‘unsupervised learning’ (an approach where the algorithm is provided with a dataset with no labeled outcomes, aiming to uncover hidden patterns or structures therein, e.g., clustering similar data points together or dimensionality reduction) [4,5,6].

Machine learning can be used widely and has been gaining popularity in various scientific and technological fields, including the diagnosis of diseases. Many recent papers have illustrated examples of disease diagnosis based on machine learning and described an increased efficiency in cost and time [7,8]. Diagnostic processes based on machine learning are considered to have few limitations, and, moreover, the procedure cannot be overwhelmed by factors associated with human nature, e.g., fatigue. More often, for the creation of models for disease diagnosis based on machine learning, data in tabular or visual form may be employed [7].

A recent topic search in the Web of Science database under the terms [[*mastitis* OR *mammary infection*] AND [*artificial intelligence* OR [*machine learning* OR *machine-learning*] OR *deep learning* OR decision tree* OR vector machine* OR *naive Bayes* OR *k-nn* OR *neuronic* OR *anomaly detection* OR *association rules* OR *recommendation systems* OR algorithm* OR architecture* OR *optimization*]] revealed a total of 65 original articles; of these, 59 articles (91%) described studies on the diagnosis of mastitis [9]. In most cases, these articles referred to the prediction of the development of acute clinical [10,11] or subclinical [12,13] mastitis in individual cows. Indeed, only one article referred to the diagnosis of mastitis at the population level in dairy cattle farms [14]. Fewer articles (14%) referred to the treatment of mastitis in cattle as guided by machine learning [15,16]. With regard to the approaches of machine learning, only one of these articles employed methodologies of unsupervised learning. The methodologies employed most frequently in the studies described in the published articles were decision trees (in 68% of published articles) and Support vector machines (in 28% of published articles) [9].

Notably, no article has been published thus far on the use of machine learning in mastitis in sheep.

Thus, the lack of any relevant studies on the application and use of artificial intelligence methodologies in ovine mastitis becomes evident. The specific objective of the present study was to develop a computational model with which predictions regarding the prevalence of mastitis in dairy sheep farms could be performed. Our hypothesis was that by employing specific variables related to flock health management and the climatological patterns present at the location of farms, predictions could be made regarding the prevalence of subclinical mastitis in sheep flocks.

## 2. Materials and Methods

### 2.1. Field Data and Dataset Used for the Construction of the Computational Model

**Figure 1:**
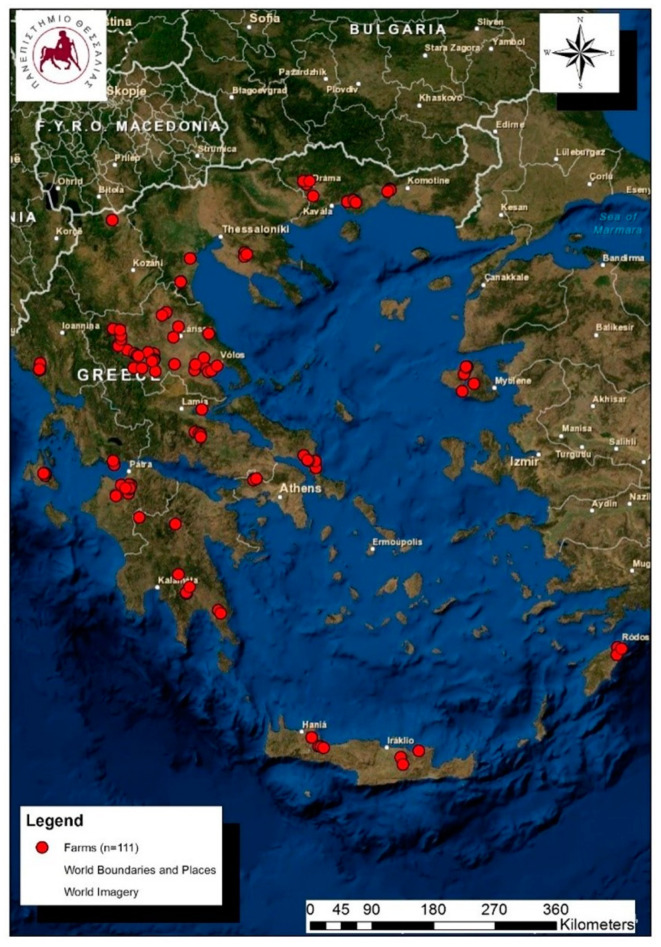
Location of 111 sheep farms around Greece that were included in a countrywide investigation on subclinical mastitis, records from which were used in the construction of the computational model.

The data employed for the construction of the computational model had been obtained during a large countrywide field study performed throughout Greece. The data were obtained during visits to sheep farms that were located in all 13 administrative regions of the country. In total, 111 dairy sheep farms were visited for the collection of samples and information (Figure 1).

All the details of the study were described by Vasileiou et al. [17]. In brief, milk samples were obtained from both mammary glands of ewes on each farm after performing a standardized clinical examination of the udder (observation, palpation, comparison between glands) and were processed by established bacteriological and cytological techniques [17]. The methodology for selection of the ewes for sampling was presented in detail by Vasileiou et al. [17] and is also described briefly hereafter: on each farm, 20 clinically healthy ewes (at least *secundiparae*) were selected for sampling (after exclusion of *primiparae* ewes and ewes with clinical mammary abnormalities) by using an electronic random number generator among animals that walked into the milking parlor. Overall, ewes sampled during this study represented 6.1% of the total ewe population in the farms (median among farms: 7.7% (interquartile range: 6.8%)) (Table S1).

Subclinical mastitis was confirmed in ewes in which a bacteriologically positive milk sample ([a] > 10 colonies of the same organism and [b] no more than two different types of colonies) with concurrently increased score (≥‘1’) in the California Mastitis Test and an increased proportion of neutrophils and lymphocytes (≥65% of all leucocytes) was detected [18,19]. During the visit to the farm, information was obtained from the farmer on management aspects applied at the farm [20].

The variables used for the construction of the computational models are described in Table 1 and further discussed in Section 4.2. In two of the farms, we obtained milk samples from ewes in two different months of their lactation period; these were used as separate records. Hence, we used 113 records for the construction of the model.

During the procedure for the development of the computational model, the following general steps were taken: (i) definition of the problem, (ii) establishment of the desired outcomes, (iii) preparation of the data, (iv) feature scaling, (v) splitting of the data and evaluation of the model, and (vi) tuning of hyperparameters (Table S2). These multiple considerations ensured the efficacy, robustness, and applicability of the computational model constructed and thereafter assessed (verified) during the study.

### 2.2. Implementation of Machine Learning Algorithms

The scikit-learn library (version 1.4.10 for Python [21]), an open-source library for machine learning that provides various tools for data mining and data analysis tasks, was employed for implementing the algorithms for the machine learning work throughout this study.

### 2.3. Evaluation for Construction of Computational Model by Means of Supervised Learning

The supervised learning methodology is applied in situations where the training data include information absent in unseen test examples. In such cases, the objective would be for the acquired expertise to predict the missing information for the data under evaluation, with the environment acting as a ‘teacher’ by providing additional information (labels). The methodology includes two main types of tasks: classification and regression. In classification tasks, the aim is to predict the categorical class labels of new instances based on previous observations, whilst in regression tasks, the aim refers to predicting a continuous numerical value based on input features. The primary difference between classification and regression tasks lies in the nature of the target variable; in classification tasks, target values are categorical (i.e., with discrete values), whilst in regression tasks, target values are continuous (i.e., with continuous values).

The first step referred to proceeding with the selection of the optimal prediction model. The method for selecting the most suitable hyperparameters of the model was based on the performance of the combination of hyperparameters in the validation set. The following supervised learning tools were applied to predict the level of prevalence of subclinical mastitis by using 17 independent variables (Table 1): decision trees, k-NN (k-nearest neighbors algorithm), neural networks, and Support vector machines. The classification of farms was predicted in one of two categories based on the level of prevalence of the infection. These two categories were created as follows: farms with prevalence of subclinical mastitis < 25.0% and farms with prevalence of subclinical mastitis ≥ 25.0%; the threshold of 25.0% was used, as it was the median target value among these 113 records.

Each of the above four tools was employed with a different combination of hyperparameters. For each combination of hyperparameters, 50 different evaluations were performed by using a combination of resampling, shuffling, and *k*-fold methods (*k* = 5). In each evaluation, the dataset was split into a ‘training set’ and a ‘validation set’. The model was set up on the ‘training set’, and its performance was evaluated on the ‘validation set’. The distribution of scores/errors for each combination of hyperparameters was assessed using boxplot, comparison of means and medians, and comparison of ranges in order to determine the optimal combination of hyperparameters that would provide the best model. In total, 543,948,800 assessments were made during this evaluation (Table S3).

Overall, in the above evaluations, using the supervised learning methods across all models, regardless of tools and combinations of hyperparameters, the mean accuracy was 51.1% for the classification of farms into one of two categories (Table S4). Hence, these model evaluations were deemed unsuitable, and they were rejected.

### 2.4. Evaluation for Construction of Computational Model by Means of Unsupervised Learning

The unsupervised learning methodology is applied when the data lack labels, i.e., when inputs are processed with no corresponding outputs. In such an approach, the algorithm operates autonomously, discovering relationships or patterns within the data without explicit guidance. The input consists of a collection of elements denoted as *X*, along with a defined distance function operating over this set. The output entails dividing the domain set *X* into distinct subsets, forming a partition, that is, $C=(C_{1},\ldots ,\;C_{k})$, where $X=\cup_{i=1}^{k}C_{i}$ and for all $i\neq j\;C_{i}\cap C_{j}=\emptyset$.

**Figure 2:**
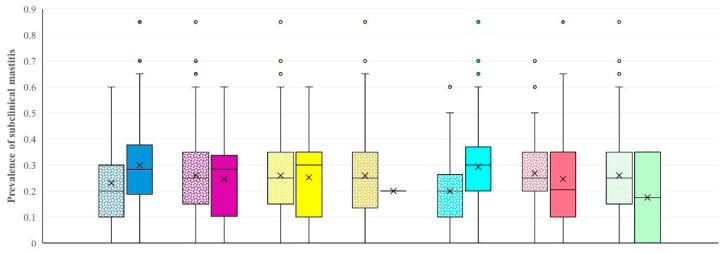
Box and whisker plots of the two clusters of sheep farms according to level of prevalence of subclinical mastitis, created by applying unsupervised learning tools (from left to right: Affinity propagation (blue), Birch threshold 3 (purple), Birch threshold 4 (yellow), Hierarchical clustering (orange), K-means (azure), Spectral clustering (red), and Spectral clustering rbf (green)).

The following unsupervised learning tools were applied in order to allocate the data into one of two clusters based on 18 variables (i.e., the 17 independent variables as described above (Table 1) and the target value, i.e., the prevalence of subclinical mastitis): Affinity propagation, Birch, Hierarchical clustering, K-means, and Spectral clustering. Based on the results of the clustering tools and models as applied by using all the above methods, the K-means tool showed the highest significance in the difference of the median prevalence of subclinical mastitis between the farms in the two clusters (‘low prevalence’ or ‘high prevalence’): the median (interquartile range) prevalence of subclinical mastitis among farms within each of the two clusters created by the K-means tool was 20.0% (interquartile range: 15.8%) and 30.0% (16.0%) (*p* = 0.002), whilst the estimated overall proportion of ewes with subclinical mastitis in the farms within each of the two clusters was 20.5% (95% confidence intervals (CI): 19.8–21.3%) and 30.4% (95% CI: 29.8–30.9%), respectively (*p* < 0.0001) (Figure 2, Table S5). Hence, the K-means tool was selected to continue with the construction of a computational model. Additionally, there were also significant differences between the two clusters created by the K-means tool in 10 of the 17 independent variables (Table S6).

K-means clustering is an unsupervised learning tool that is employed when dealing with numerous individual data points, each represented by vectors, where each entry within the vector denotes a specific feature. However, these data points lacked pre-assigned labels or classifications. These data points are organized into coherent groups, each group being linked to its respective center of mass, i.e., the centroids. The K-means algorithm aims to calculate the centroids that minimize the following: $\sum_{i=0}^{n}\underset{\mu_{j}\in C}{\min}{\left\|x_{i}-\mu_{j}\right\|}^{2}$.

### 2.5. Selection and Application of Computational Model

#### 2.5.1. Procedures

For the selection of the computational model, supervised learning tools were again applied to predict the classification of records into clusters of ‘low prevalence’ or ‘high prevalence’ created by the K-means tool by using the 17 independent variables, as detailed previously. On this occasion, classification was performed in order to predict the category for the level of prevalence of the infection. The following supervised learning tools were employed to develop the computational model for prediction: decision trees, k-NN, neural networks, and Support vector machines.

Decision trees embody a non-parametric supervised learning approach suitable for both classification and regression assignments. A decision tree is a predictor p:X↦Y, where a function from the space X of the features to the discrete space Y; the most common situation is Y={0,1} being binary. Usually, the splitting is based on one of the features of *x* or on a predefined set of splitting rules. Their purpose is to build a model capable of forecasting the value of a target variable by deriving simple decision rules from the dataset features. Essentially, such a tree functions as a piece-wise constant estimation. Serving as a hierarchical decision-support model, decision trees delineate decisions and their likely consequences, encapsulating chance occurrences, resource allocations, and utility assessments. Acting as a decision-support hierarchical model, decision trees outline decisions and their probable outcomes, encompassing chance events, resource expenses, and utility considerations, and they include several hyperparameters that can be adjusted to control the behavior and performance of the model. In the present study, we used the following hyperparameters tuned to optimize the performance of our model; for maximum depth, the nodes of the model were expanded until all leaves were pure or until all leaves contained fewer than the minimum number of samples required to split an internal node, in this case, 2. The minimum number of samples required to be at a leaf node was defined to be equal to 1.

k-NN, which is a neighbor-based classification, belongs to the realm of instance-based learning or non-generalizing learning, and, unlike methods that aim to construct overarching internal models, it simply retains instances of the training data. Classification is determined by a straightforward majority vote among the closest neighbors of each data point: a query point is assigned to the class that is most prevalent among its nearest neighbors. In classification problems, this approach would choose the category based on the majority category of the k nearest neighbors, while in regression problems, it would select the value using the weighted mean function, i.e., in order to estimate the value $\hat{f}\left(x_{i}\right)$, the relevant calculation would be $\hat{f}\left(x\right)=\sum_{i=1}^{k}\frac{f\left(x_{i}\right)}{k}$. Additionally, one can opt for more sophisticated functions, such as weighting by the inverse of distances, described as follows:

(1) $$\hat{f}\left(x\right)=\left\{\begin{array}{ll} \sum_{i=1}^{k}\frac{w_{i}\left(x_{i}\right)f\left(x_{i}\right)}{\sum_{i=1}^{k}w_{i}\left(x_{1},\ldots ,x_{k}\right)} & \mathrm{if}\;d\left(\vec{x},{\vec{x}}_{i}\right)\neq 0\;\forall \;i\leq k\\ f\left(x_{i}\right) & \end{array}\right.$$

or for more mathematically complex distance functions, like exponentially weighted by distance or using a Gaussian function (Gaussian kernel). Within the k-NN algorithm, there is the flexibility to change the distance function. A common approach involves exploring various values for the Minkowski distance, $d\left(\vec{x},\vec{y}\right)={\left(\sum_{i=1}^{n}\left|x_{i}-y_{i}\right|\right)}^{\frac{1}{p}}$, which allows for adapting the distance metric to better suit the characteristics of the dataset. The k-NN classification method is widely employed, with the optimal selection of the value *k* being highly contingent on the dataset. Generally, a larger *k* mitigates the impact of noise but may also lead to less well-defined classification boundaries. In the present study, for the number of neighbors (*k*), we specified the number of nearest neighbors to consider when making predictions as *k* = 1 to 10, and we also used two hyperparameters that could be tuned to optimize the performance of the model: for the distance metric, we used the distance metric of Euclidean distance, which is the Minkowski distance for *p* = 2; and for the weight function, we employed the uniform approach where all points in each neighborhood would be weighted equally.

The neural networks, as structures, have garnered significant attention in recent times and have emerged as a fundamental concept in contemporary machine learning. Their origins trace back to the inspiration drawn from the functioning of the human brain. Indeed, the inception of the first neural network can be attributed to the work of McCulloch and Pitts [22], who sought to model a biological neuron. A ‘McCulloch and Pitts’ neuron is a function $f:R^{d}\mapsto \left\{0,1\right\}$, with

(2) $$f\left(x_{1},\ldots ,x_{d}\right)=I_{R^{+}}\left(\sum_{i=1}^{d}w_{i}x_{i}-\theta \right)$$

where $w_{i},\theta$ are real numbers, d is a natural number, and $I_{R^{+}}$ is the real function with $I_{R^{+}}=0$ for x<0 and $I_{R^{+}}=1$ for x≥0. In the context of a neural network framework, the function $I_{R^{+}},$ termed activation function, θ, termed threshold, and the $w_{i}$ are termed weights. A more sophisticated model is the multilayer perceptron, which is the fundamental construction. Herein, we adopted the scheme proposed by Berner et al. [23]. A fully connected feedforward network is provided by its architecture $\left(N,\rho \right),$ where $L\in N,N\in N^{L+1}$ and ρ:R↦R, where ρ represents the activation function, L stands for the number of layers, and $N_{0},N_{L},N_{l}$ with $l\in \left[1,L-1\right]\subset N$ are the numbers of neurons in the input, output, and the l-th hidden layer, respectively. It is noted that the number of parameters is denoted by $P\left(N\right):=\sum_{l=1}^{L}N_{l}N_{l-1}+N_{l}$. Then, we defined the corresponding realization function $\Phi_{a}:R^{N_{0}}\times R^{P\left(N\right)}\mapsto R^{N_{L}}$, which satisfied that for every input $x\in R_{0}^{N}$ and parameters θ, where $\theta ={\left(\theta^{\left(l\right)}\right)}_{l=1}^{L}={\left(\left(W^{\left(l\right)},b^{\left(l\right)}\right)\right)}_{l=1}^{L}\in \prod_{l=1}^{L}\left(R^{N_{l}\times N_{l-1]}}\times R^{N_{l}}\right)$, meant that for every l the $W^{\left(l\right)}$ was a real matrix and $b^{\left(l\right)}$ was a vector, where $\Phi_{a}\left(x,\theta \right)=\Phi^{\left(L\right)}\left(x,\theta \right)$ and

(3) $$\begin{array}{r} \Phi^{\left(1\right)}\left(x,\theta \right)=W^{\left(1\right)}x+b^{\left(1\right)}\\ {\hat{\Phi }}^{\left(l\right)}\left(x,\theta \right)=\rho \left(\Phi^{\left(l\right)}\left(x,\theta \right)\right)\;\mathrm{with}\;l\in \left[1,L-1\right]\\ \Phi^{\left(l+1\right)}\left(x,\theta \right)=W^{\left(l+1\right)}{\hat{\Phi }}^{\left(l\right)}\left(x,\theta \right)+b^{\left(l+1\right)}\;\mathrm{with}\;l\in \left[1,L-1\right] \end{array}$$

and ρ was applied component-wise. Also, we referred to the matrices $W^{\left(l\right)}$ as the weighted matrices and to the vectors $b^{\left(l\right)}$ as the bias vectors. Further, we referred to ${\hat{\Phi }}^{\left(l\right)}$ and $\Phi^{\left(l\right)}$ as activation and pre-activation functions of the $N_{l}$ neurons in the l-th layer. The width and the depth of the neural networks were defined as $∥N{∥}_{\infty }$ and L, respectively. Moreover, we used as hyperparameters (a) the activation function, which included ‘*identity*’, ‘*logistic*’, ‘*tanh*’, and ‘*relu*’, (b) the number of hidden layers, with values of 10, 20, 50, 100, or 300, (c) the learning rate with the default value (0.001), and (d) the solver for weight optimization, which was ‘*lbfgs*’, ‘*sgd*’, or ‘*adam*’.

Support vector machines refer to powerful supervised learning models that work by constructing a hyperplane or a set of hyperplanes in a high-dimensional or even infinite-dimensional space, depending on the characteristics of the dataset. If it is considered that $S=\{\left(x_{1},y_{1}\right),\ldots ,\left(x_{n},y_{n}\right)\}$ is a training dataset where $x_{i}\in R^{d}$ and $y_{i}\in \left\{-1,1\right\},$ and this raining dataset is called linearly separable, indicating that such a hyperplane (hyperspace) $\left(w,b\right)$ exists that for $x_{i}$ holds that $y=sign\left(\left\langle w,x_{i}\right\rangle +b\right)$, the previous can be alternatively expressed in the form of inequalities as $y_{i}\cdot \left(\left\langle w,x_{i}\right\rangle +b\right)>0\;\;\mathrm{for}\;\mathrm{all}\;\;x_{i}$. The hyperplanes (hyperspaces) with this property are infinite; therefore, in order to obtain the optimal solution, it is necessary that $\arg\underset{\left(w,b\right)}{min}{\left|w\right|}^{2}\;\;\mathrm{for}\;\mathrm{all}\;x_{i}\;:\;y_{i}\cdot \left(\left\langle w,x_{i}\right\rangle +b\right)\geq 1$. There are several formulations for problem cases like the previous one that employ additional restrictions like the regularization terms, where the previous equation transforms to $\underset{w,b,\xi }{min}\left(\lambda ∥w{∥}^{2}+\frac{1}{m}\sum_{i=1}^{m}\xi_{i}\right)\;\;\mathrm{for}\;\mathrm{all}\;x_{i}\;:\;y_{i}\cdot \left(\left\langle w,x_{i}\right\rangle +b\right)\geq 1-\xi_{i}\;\mathrm{and}\;\xi_{i}\geq 0$.

When data under assessment cannot be clustered in a linear manner, a more sophisticated approach would be to embed the dataset into a higher feature space using the ‘trick’ of the kernel. The kernel function $K\left(x,x^{\prime }\right)=\left\langle \psi \left(x\right),\psi \left(x^{\prime }\right)\right\rangle$, where the function ψ refers to some domain space into some Hilbert space. The more commonly used kernels are: (a) the Gaussian kernel, defined as $K\left(x,x^{\prime }\right)=e^{-\frac{∥x-x^{\prime }{∥}^{2}}{2\sigma }}=e^{-\gamma ∥x-x^{\prime }{∥}^{2}}$, (b) the polynomial kernels, defined as $K\left(x,x^{\prime }\right)={\left(1+\gamma <x,x^{\prime }>\right)}^{k}$, and (c) the sigmoid kernel, defined as $K\left(x,x^{\prime }\right)=tanh\left(-\gamma <x,x^{\prime }\geq +r\right)$. In the present study, we used two hyperparameters tuned to optimize the performance of the model, specifically, the kernel (‘*linear*’, ‘*poly*’, ‘*rbf*’ or ‘*sigmoid*’; termed according to the Python scikit-learn library (version 1.4.10 for Python [21])) and the regularization parameter as C = 1, 2 or 3.

For each of the above tools, various combinations of hyperparameters were employed, as detailed in Table 2. During the process, 83 models were produced, and 4150 assessments were made in total.

#### 2.5.2. Data Management

Within each tool employed for the assessment, we compared the results of the 50 different evaluations made using each model by means of the following three measures of quality: (a) accuracy, (b) precision, and (c) recall. The best model produced by each tool was selected for comparison across the four tools employed. Subsequently, a comparison among tools was also performed by using the above three measures of quality.

The Kruskal–Wallis test was used to compare the measures of quality between models within tools, as well as between tools. Statistical significance was defined at *p* < 0.05.

### 2.6. Assessment (Verification) and Evaluation of Results of Computational Model

#### 2.6.1. Field Data and Datasets Used for the Construction of the Assessment (Verification) of Computational Model

Subsequent to the selection of the best model for the classification of records from sheep farms based on the level of prevalence of subclinical mastitis, that model was assessed by inputting records from two field studies that were performed in sheep flocks in Greece subsequent to the initial field study and independent of that one.

The first field study involved 12 sheep farms, which were visited four times during a lactation period, with repeated samples obtained from the same ewes throughout the lactation period; therefore, records from 48 farm visits to these flocks were available [24]. None of these farms were included in the initial field study, i.e., that used for the training of the model. In this field study, methods (sampling, laboratory techniques, etc.) identical to those employed during the initial field study were used.

**Figure 3:**
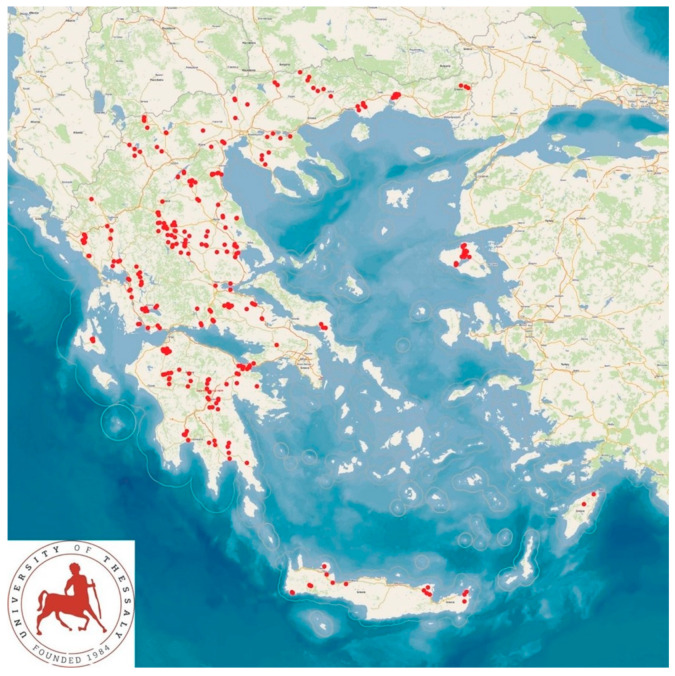
Location of 325 sheep farms around Greece that were included in a countrywide investigation, records from which were used in the assessment (verification) of the computational model selected.

The second field study involved 325 sheep farms located in the 13 administrative regions of the country (Figure 3), which were visited once [25]. Again, none of these farms were included in the initial field study. In this study, bulk-tank milk samples were collected for somatic cell counting. The prevalence of subclinical mastitis in these flocks was subsequently estimated by using the correspondence described by Fthenakis [26], based on the somatic cell counts in the bulk-tank milk of the farms.

In both of the above studies, information was obtained from the farmer on management aspects applied on the farm [20].

#### 2.6.2. Procedures

For assessment (verification) of the computational model selected previously, initially, each one of the 373 records (i.e., from the 48 visits (to 12 farms, each visited four times) and the 325 visits (to 325 farms, each visited once)) taken individually with the initial 113 records was clustered by using the K-means tool in unsupervised learning, as described in detail previously. In total, 373 sets of records were created: each of these sets included (a) the 113 records as above and (b) one of the 373 records as above (i.e., 114 records in each set).

Subsequently, the supervised learning model previously selected was used to evaluate the correct clustering of the test record within each of the 373 sets. Each of these 373 sets was assessed by using this model, each of which included a training subset (113 records, i.e., those employed previously, as detailed above) and a test (prediction) subset (one record). The record included in the test (prediction) subset was different in each of the 373 sets. The computational model used predicted the assignment of each of the 373 records into a class ‘0’ or ‘1’ that corresponded to the level of prevalence.

#### 2.6.3. Data Management

The class into which each record was classified by the model was compared to the cluster of farms, into which each of the records had been previously clustered by using the K-means tool. Further, the predicted classification of each record by the computational model into a category of the level of subclinical mastitis was evaluated against the prevalence of subclinical mastitis found in the respective farm.

The overall accuracy of the predictions was calculated as the proportion of farms correctly classified by the computational model. This was calculated separately for (a) the results obtained by using the K-means tool and (b) the prevalence of the infection on each farm. The proportion of farms with a prevalence of subclinical mastitis of (a) ≥20.0% or <20.0%, (b) ≥25.0% or <25.0%, and (c) ≥30.0% or <30.0%, correctly classified by the computational model into the ‘high prevalence’ (i.e., with prevalence of subclinical mastitis ≥ 20.0%, ≥25.0%, or ≥30.0%, respectively) or ‘low prevalence’ (i.e., with prevalence of subclinical mastitis < 20.0%, <25.0%, or <30.0%, respectively) category, was also calculated. The predicted prevalence referred to the veterinary diagnosis of the prevalence of subclinical mastitis in the flocks, i.e., by taking into account the results of a combination of bacteriological and cytological tests [18,19].

Comparisons between accuracies were made by using Pearson’s chi-square test. The median prevalence of subclinical mastitis in farms classified among each of the two categories of sheep farms created by the computational model was compared by using the Mann–Whitney test. Statistical significance was defined at *p* < 0.05.

#### 2.6.4. Analysis of the Importance of the 17 Independent Variables in Predicting the Prevalence of Subclinical Mastitis—Interpretation of Findings

SHAP (SHapley Additive exPlanations) values analysis, which is a means to explain the output of a computational model based on machine learning methodology, was employed in order to understand how the 17 individual variables used in the computational model influenced the predictions when using the model [27]. SHAP quantified feature importance based on principles of game theory and revealed how each feature contributed to the final output of the model. Through the use of the SHAP Python library, SHAP values were calculated for each prediction [27].

The values found represented the impact of each feature (i.e., each of the 17 independent variables) on the prediction’s deviation from the baseline. SHAP determined this impact by assessing how the prediction changed as features were progressively added to the model in all possible combinations [27].

## 3. Results

### 3.1. Selection of Best Computational Model

**Figure 4:**
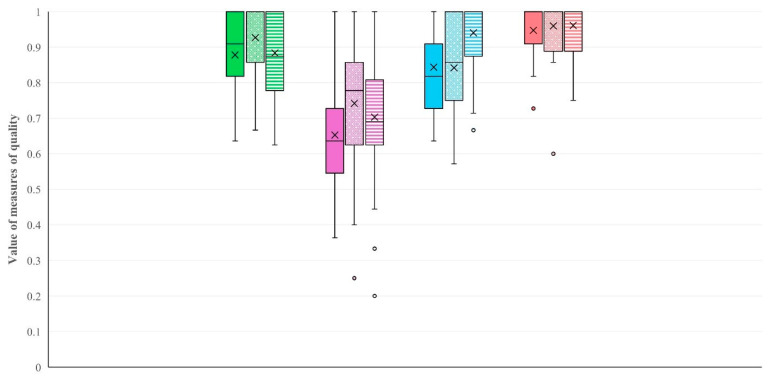
Box and whisker plots of the data for measures of quality in each of the four models selected after evaluation within each tool used during assessment for classification of records from sheep farms based on prevalence of subclinical mastitis (green: decision trees, purple: k-NN, blue: neural networks, pink: Support vector machines—full pattern: accuracy, dotted pattern: precision, striped pattern: recall).

The results of the evaluations performed by means of each model and each tool, which were employed for model selection, are presented in detail in Tables S7–S10. The measures of the center of the data for the measures of quality in each of the four models selected after evaluation within each tool are shown in Figure 4 and Table 3. There was clear evidence that the differences in the measures of quality among tools were significant (*p* < 0.0001 for all comparisons). Based on these, the model obtained by means of Support vector machines (kernel: ‘*linear*’, regularization parameter C = 3) was considered the best one for the classification of records obtained from the sheep farms based on the level (i.e., low/high) of the prevalence of subclinical mastitis.

### 3.2. Assessment (Verification) of the Previously Selected Computational Model

**Figure 5:**
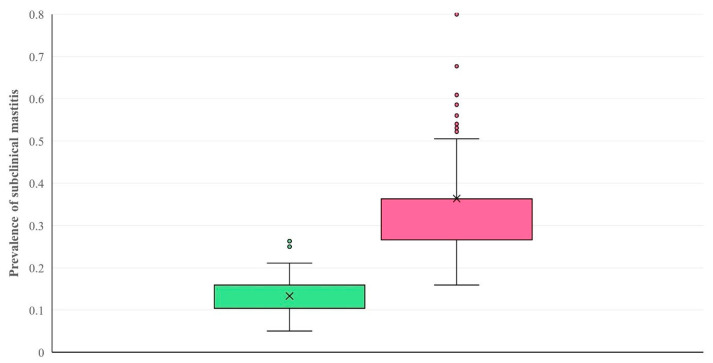
Box and whisker plots of the two categories of sheep farms according to predicted level (i.e., low/high) of prevalence of subclinical mastitis, created by using Support vector machines (green: farms assigned in category with low level of prevalence; pink: farms assigned in category with high level of prevalence).

Of the 373 records used in the assessment (verification) of the computational model (Support vector machines), this model correctly predicted and classified 351 records in either of the two categories, which corresponded to the two clusters previously created by the K-means tool. The overall accuracy of the computational model versus the results presented by the K-means tool was 94.1% (95% confidence interval (CI): 91.2–96.1%). The median prevalence of subclinical mastitis in farms within each of the two categories was 10.4% (interquartile range: 5.5%) and 36.3% (9.7%) (*p* < 0.0001) (Figure 5).

The highest accuracy of the computational model was achieved when considering ‘low prevalence’ and ‘high prevalence’ of subclinical mastitis at <25.0% and ≥25.0%, respectively. With this, the model correctly predicted 359 (of the 373) records corresponding to farms with respective levels of prevalence of the infection; thus, the overall accuracy for the estimation of the level of prevalence in the farms used for assessment (verification) of the model was 96.3% (95% CI: 93.8–97.8%) (*p* = 0.17 for comparison of results versus those obtained by the K-means tool). The proportion of farms with prevalence of subclinical mastitis < 25.0% correctly predicted by the computational model was significantly higher than the respective proportion of farms with prevalence ≥ 25.0%: 99.2% (95% CI: 97.1–99.9%) versus 90.7% (95% CI: 84.5–94.6%), respectively (*p* < 0.0001) (Table 4).

With the threshold at 20.0%, the model predicted 353 records corresponding to farms with respective prevalence of the infection; thus, the overall accuracy was 94.6% (95% CI: 91.9–96.5%). With the threshold at 30.0%, the model predicted 334 records corresponding to farms with respective prevalence of the infection; thus, the overall accuracy was 89.5% (95% CI: 86.0–92.3%) (*p* = 0.0006 for comparison of accuracy achieved with each of the three thresholds) (Table 4).

**Figure 6:**
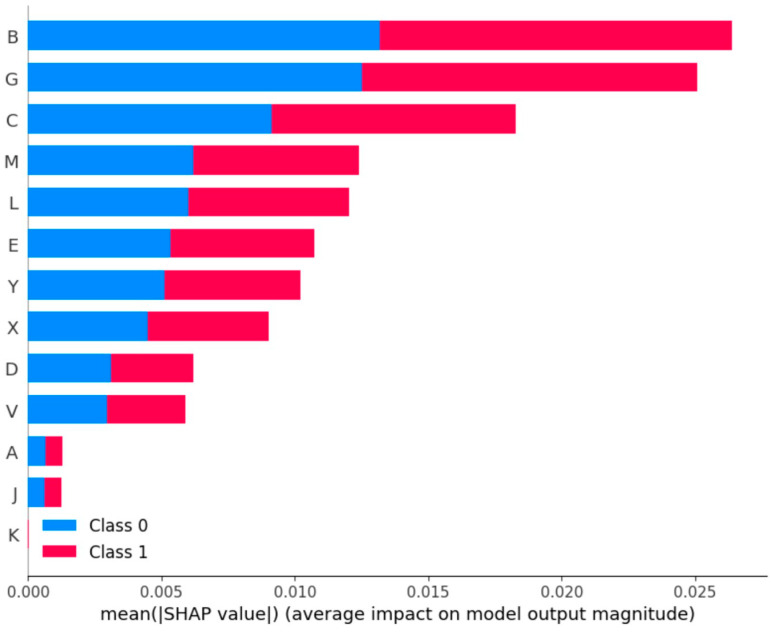
SHapley Additive exPlanations values for the importance of each of 17 independent variables in the prediction of the level (i.e., low/high) of prevalence of subclinical mastitis (B: breed of ewes, G: vaccination against staphylococcal mastitis, C: management system applied in farm, M: administration of antibiotics at the end of the lactation period, L: application of measures for mastitis control at the end of the lactation period, E: application of reproductive control, Y: altitude of farm location, X: wind speed at farm location, D: month of lactation period, V: annual precipitation at farm location, A: no. of ewes in farm, J: milking status of the ewes in farm, K: application of teat dipping; not included in output: minimum temperature of coldest month at farm location, distance of farm from other sheep farms, land use at farm location, and microhabitat at farm location) (class 0/1: low (<25.0%)/high (≥25.0%) level of prevalence of subclinical mastitis) during assessment (verification) of a computational machine learning model.

The results of the analysis for SHAP values for the impact of each of the 17 independent variables in the prediction of subclinical mastitis have indicated that (a) the breed of ewes, (b) the application of vaccination against staphylococcal mastitis, and (c) the management system applied in a farm were the variables that most influenced the prediction outcome. That impact was similar for farms with low (<25.0%) or high (≥25.0%) prevalence of subclinical mastitis (Figure 6, Table 5).

## 4. Discussion

### 4.1. Preamble

The manuscript presents the first attempt internationally to develop, evaluate, and make available a prediction model for the prevalence of subclinical mastitis in dairy sheep farms.

With regard to cattle mastitis, the relevant publications have focused on predicting the development of the infection in individual animals. This can be explained given the significant difference in value between the price of a cow and a ewe. Moreover, for predictions at the individual cow level, an increased amount of data for setting up the model and for effective ‘model training’ can be obtained through the daily records of animals. The wide use of electronic automatic monitoring systems in cattle farms facilitates the collection of such high numbers of data. Through these, details about various parameters, e.g., milk flow and milking time, volume of milk produced, protein and lactose concentration in milk, and milk electrical conductivity (EC), can be monitored and obtained, providing ample data for developing relevant models. In that way, high numbers of records can be collected and used; Ebrahimie et al. [28] used 345,000 milking records from individual cows, and Pakrashi et al. [13] used the records of 1,350,000 milk-days from 2390 cows. In contrast, many dairy sheep farms would not possess such equipment (e.g., in Greece, in 21.5% of sheep farms, hand-milking is still applied, whilst only 1.2% of milking parlors are equipped with automated monitoring systems [29]), which makes the collection of such data difficult at the individual animal level.

Thus, in dairy sheep, the prediction models would need to focus on the flock level, where the adverse effects of mastitis can be significant [30]. Whilst clinical mastitis is easy to diagnose based on the observation and clinical examination of animals [31,32], the diagnosis of subclinical mastitis requires the concurrent application of bacteriological and cytological techniques on milk samples [18,19]; moreover, the estimation of the prevalence of the infection within a flock requires the sampling of several animals. Hence, there is an interest in a tool for the prediction of the prevalence of the infection in sheep farms. This can be used by veterinarians active in the health management of sheep farms in order to make clinical decisions during field assessments of flocks.

### 4.2. Development of the Model

We have input into the model data from a large countrywide field study performed in Greece [17]. The study included farms located in all 13 administrative regions of the country; thus, a variety of locally applied practices and location-related factors were included. A total of 17 independent variables related to practices related to health management applied in the farms, as well as climatological parameters prevailing at the locations of the farms, were input as independent variables and were used to predict the prevalence level of subclinical mastitis in the flock.

The health management-related parameters included conditions found to be associated with the development of subclinical mastitis, e.g., sheep breed [33,34], or found to contribute to preventing the infection, e.g., anti-mastitis vaccination [35,36] or the so-termed ‘dry-ewe mastitis treatment’ [37,38,39]. The inclusion of the stage of lactation among these variables is also noted, given that the prevalence of subclinical mastitis progressively increases as the lactation period advances [30,40,41]. That way, the prevalence of the infection could be predicted more accurately and in accord with the stage of the lactation period when a clinical assessment would be taking place.

The inclusion of climatological parameters is in accord with findings related to the potential effects of weather conditions in the development of the infection. Early during the previous century, Leyshon [42] reported that mastitis in ewes occurred more often during cold weather conditions; nevertheless, in cows, evidence has been published indicating that in increased temperatures, mastitis could be more frequent [43], possibly because increased temperatures can play a role in reducing leucocyte counts in sheep [44] and impairing their function [45,46], thus compromising mammary defenses and rendering animals more susceptible to mastitis. More recently, similar relevant findings regarding the importance of climatic factors in the development of mastitis were also reported from sheep flocks [47]. Therefore, there was a scope to include climatological data related to the location of the farms in the model, as these may play a role in the development of the infection. It is interesting that in the only published study regarding the prediction of bovine mastitis prevalence at the farm level, weather-related data have also been included to improve the robustness of the model [14].

Mastitis in sheep is a multifactorial disease [30,41,48]. This has been taken into account and reflected in the increased number of independent variables (*n* = 17) employed for the development of the model, which provides an increased number of parameters associated with sheep health and which could be taken into account for making relevant predictions and has thus contributed to its high measures of quality. On the other hand, the inclusion of a higher number of parameters would have overemphasized patterns characteristic of the training data employed in this study [49,50] and, moreover, would make more difficult the collection of data for performing predictions about the prevalence of the infection in clinical settings.

In studies of machine learning applied for the prediction of mastitis in cattle, decision trees were found to be the tool employed more frequently, specifically in 72% of the relevant articles found during the literature search. The application of Support vector machines has been reported less frequently, in only 31.5% of the articles. In the present study, four supervised learning tools were employed, and Support vector machines provided the best measures of quality; thus, it was selected. The Support vector machines methodology was developed specifically for high-dimensional data problems, which explains its tendency to provide higher performance. Decision trees also performed well, followed by neural networks. Although k-NN is often preferred in studies focusing on classification prediction due to its simplicity, scalability, and ease of understanding, in the present study, it did not show good performance in predicting the suite of variables examined. In this context, it is also noted that in the only published study related to predictions of mastitis at the farm level in cattle, the Support vector machines tool was employed [14].

The development of Support vector machines was based on foundations of robust regression [49,50]. The tool can map response variables to a higher-dimensional space, which includes a ‘maximal separating hyperplane’. The target value would need to separate across this hyperplane into correct classifications [51]. The tool also allows for greater flexibility by including various kernel functions within it [49,50]. In the present study, four different kernel functions (linear, polynomial, radial basis, and sigmoid) were tested, each of which was combined with one of three different regularization parameters during the testing of various models in this specific tool.

Support vector machines can outperform decision trees, neural networks, and k-NN due to their effectiveness in high-dimensional spaces, making them well-suited for tasks involving a large number of features. The tool incorporates regularization parameters that aid in controlling overfitting, thus ensuring better generalization performance. Moreover, the methodology has a lower susceptibility to issues such as vanishing gradients, which can impede the training of neural networks, particularly when data are limited. Additionally, the use of Support vector machines typically requires fewer hyperparameters compared to neural networks, which simplifies the tuning process and reduces sensitivity to parameter selection.

### 4.3. Assessment (Verification) of the Model

The validity of the computational model deemed to be the best was assessed by evaluating the accuracy of predicting the level of subclinical mastitis in sheep farms unrelated to the ones used for the ‘training’ and development of the model. Data from these farms originated from two large-scale field studies that we had carried out subsequent to the initial study, i.e., the one on which the development of the model was based. In the first of these, farms were visited repeatedly, which allowed us to assess the same farms at differing stages of the lactation period [24]. In the second of these, farms were visited across the country as part of a large countrywide study of mapping the sheep industry in Greece [25,29]. Collectively, the 373 records used in the assessment (verification) of the model provided a variety of practices related to health management, time points within the lactation period, climatological conditions, and geographical locations, which, taken together, have provided a wide representation of the conditions in dairy sheep farms across Greece.

For the assessment (verification) of the model, we opted to use a test (prediction) subset that consisted of only one record on each occasion. This simulated field conditions closely, as, under clinical settings, predictions of the level of prevalence of subclinical mastitis would refer to one farm being under clinical investigation.

The categorization of sheep farms into ones with ‘low prevalence’ and ones with ‘high prevalence’ of subclinical mastitis can help to predict whether the prevalence of the infection in a flock under investigation is below or above average, as well as to provide information and consider variables that may affect this. This way, health management measures may be taken, and corrective actions may be initiated in order to improve the situation and lead to a decrease in the prevalence of the infection.

The emergence of the variables with the greatest impact among those requiring simple and easy-to-obtain answers during visits to farms (e.g., breed of ewes, vaccination against staphylococcal mastitis, management system applied in the farm, administration of antibiotics at the end of the lactation period, application of measures for mastitis control at the end of the lactation period, and application of reproductive control) for the prediction of subclinical mastitis makes the procedure relatively straightforward for clinicians. Key properties of SHAP values making them useful for model interpretation include (a) additivity (allowing for efficient computation, even in high-dimensional datasets), (b) local accuracy (providing an accurate, localized interpretation of the model’s prediction for a specific instance), (c) missingness (making SHAP values robust to missing data and ensuring that irrelevant features do not distort the interpretation), and (d) consistency (ensuring similar interpretation of the model’s behavior, even as the model’s architecture and parameters evolve) [27].

Moreover, the identification of these variables reflects, to a large extent, the adjustments that can be made in management practices in sheep flocks in order to improve the control of subclinical mastitis.

### 4.4. Overall View of the Procedure for Model Development and Assessment (Verification)

Overall, this study presented the development of a computational model for the classification of records into categories corresponding to clusters, which had been previously established by means of the K-means algorithm; then, it assessed its applicability and performance in clinical conditions. The optimal tool for this model, Support vector machines, correctly predicted and classified records (i.e., sheep farms), with an overall accuracy of 94% in comparison to the veterinary diagnosis of subclinical mastitis in the flocks, as based on the results of a combination of bacteriological and cytological tests [18,19]. A high accuracy of the computational model was achieved, with the threshold for the prevalence of the infection set at 25.0% to separate farms into ones with ‘low prevalence’ or ‘high prevalence’. This corresponds to the median target value of the initial set of 113 records, based on which the initial K-means clustering was performed. In this case, the model achieved an accuracy of 96%. This can be considered an indirect confirmation of the validity of the methodology employed in this study. It is also notable that a 25.0% prevalence of ewes with suboptimal milk production within a flock (the main reason for which is subclinical mastitis) is necessary to confirm a diagnosis of the ‘milk-drop syndrome of ewes’ [1]. This way, the optimum threshold in the prediction model goes together with the prevalence of suboptimal milk production in the flock. Specifically for farms with a prevalence of infection < 25.0%, the accuracy was as high as 99%, again in comparison to a veterinary diagnosis of subclinical mastitis in the flocks.

These findings confirm the strong performance of the Support vector machines tool for the classification of sheep flocks according to low or high prevalence of subclinical mastitis by using the 25.0% prevalence rate threshold.

### 4.5. Potential Constraints of the Proposed Model

During the development of a computational model, errors may mainly derive from the following sources:

- Data quality issues, for example, missing data (i.e., incomplete records, which can skew the model’s understanding and performance), outlying values (i.e., extreme values, which may influence a model disproportionately), or biased data (especially in models sensitive to anomalies). In our case, the use of a structured questionnaire for the collection of detailed information from farmers [20], the high number of farms considered for the construction and the assessment (verification) of the model, and the countrywide location of the farms have minimized such issues. In particular, findings from farms in all regions of Greece were taken into account during all stages of this study; this way, conditions prevailing throughout the country were taken into account, and factors of regional importance weighed less.
- Model overfitting (when a model ‘learns’ the training data too well and cannot discriminate ‘noise’ values and outliers, its performance on new data is harmed) or underfitting (when the principles for the development of a model are too simplistic to capture the underlying patterns within the data, leading to poor performance even on the training data). In our case, the use of variables with confirmed scientific significance for the development of mastitis has reduced those risks. In this context, many health-related factors have thus been assessed and included in the model that was finally developed.
- Model selection issues, for example, inappropriate model choice (i.e., use of a model unsuitable for the type of data available or the specific problem under investigation) or hyperparameter tuning (i.e., use of suboptimal hyperparameter settings) can lead to poor performance of a model. In our case, the use of supervised and unsupervised learning methodologies and the evaluation of a variety of tools and methodologies have lowered the relevant risks.

It is noted that the developed model is of value primarily for dairy sheep farms. Whilst there are similarities between sheep flocks in accordance with the type of production system (e.g., dairy, meat), there are also significant differences in various management-related practices (for example, the lack of milking in the latter flocks). Hence, the developed model needs to be carefully evaluated and appropriately tuned before potential usage in sheep farms applying a different production type.

Addressing these constraints is crucial for unlocking the full potential of machine learning methodologies in the detection of subclinical mastitis in ewes.

## 5. Conclusions

A prediction model for the prevalence of subclinical mastitis in sheep flocks has been developed using field data, and it showed superior performance. The model may function as a valuable tool for supporting decisions made by clinicians in formulating control schemes of mastitis in dairy sheep. The findings of this study indicate that machine learning algorithms can be usefully employed in predicting the level of subclinical mastitis in dairy sheep farms. Machine learning can take into account routinely available data (e.g., information about practices related to health management and climatological records) in order to provide predictions regarding the level of a financially significant disease of sheep. The binary prediction of the infection prevalence can help clinicians, particularly in intricate scenarios where forecast errors are more likely. A combination of unsupervised and supervised learning methodologies was applied in this study. The K-means and Support vector machines were the specific tools found to provide the best measures of quality in the study. The identification of variables with a major impact on the prediction of subclinical mastitis supports the application of relevant adjustments in health management in sheep flocks to facilitate control of subclinical mastitis.

The findings will facilitate setting up appropriate measures and making interventions in dairy sheep farms. Future research could explore further areas, for example, fine-tuning model parameters and incorporating additional data sources to improve the output of the model. In all, machine learning can advance sheep farming by addressing challenges, improving decision-making processes, and enhancing veterinary clinical work and professional outputs.

## Figures and Tables

**Table 1:** Variables used for the construction of the computational model.

**1. Target Value**
Level of prevalence of subclinical mastitis in farm (%) (binary)
**2. Independent Variables**
Number of ewes available in the flock (numeric)
Management system applied in the farm (categorical)
Breed of ewes (categorical)
Month of lactation period (numeric)
Application of reproductive control (categorical)
Vaccination against staphylococcal mastitis (categorical)
Milking status of the ewes in the farm (categorical)
Application of teat dipping (categorical)
Application of measures for mastitis control at the end of the lactation period (categorical)
Administration of antibiotics at the end of the lactation period (categorical)
Minimum temperature of coldest month at farm location (numeric)
Annual precipitation at farm location (numeric)
Wind speed at farm location (numeric)
Altitude of farm location (numeric)
Distance of farm from other sheep farms (numeric)
Land use at farm location (categorical)
Microhabitat at farm location (categorical)

**Table 2:** Supervised learning tools used, hyperparameters employed, and numbers of models produced during assessment for classification of records from sheep farms based on level of prevalence of subclinical mastitis.

Supervised Learning Tool	Hyperparameters	No. of Different Models Produced
Decision trees	maximum depth, minimum number of split samples	1
k-NN	distance metric, K, weight function	10
Neural networks	activation function, hidden layers, learning rate, solver	60
Support vector machines	kernel, regularization parameter	12

**Table 3:** Measures of the center of the data for the measures of quality in each of the four models selected after evaluation within each tool used during assessment for the classification of records from sheep farms based on the level of prevalence of subclinical mastitis.

Supervised Learning Tool	Details of Models Employed	Measures of Quality of Models Employed		
		Accuracy ^1^	Precision	Recall
Decision trees	- maximum depth: 2, - minimum number of split samples: 1	0.878 ± 0.015 0.909 (0.159) 0.909	0.927 ± 0.017 1.000 (0.143) 1.000	0.874 ± 0.017 0.917 (0.217) 1.000
k-NN	- distance metric: *p* = 2, - K = 1, - weight function: uniform approach	0.653 ± 0.019 0.636 (0.182) 0.727	0.742 ± 0.024 0.777 (0.226) 1.000	0.703 ± 0.024 0.690 (0.175) 0.667
Neural networks	- activation function: ‘*logistic*’, - hidden layers: 300, - learning rate: 0.001, - solver: ‘*adam*’	0.844 ± 0.014 0.818 (0.159) 0.818	0.842 ± 0.017 0.857 (0.222) 1.000	0.940 ± 0.013 1.000 (0.125) 1.000
Support vector machines	- kernel: ‘*linear*’, - regularization parameter C = 3	0.947 ± 0.010 1.000 (0.091) 1.000	0.960 ± 0.011 1.000 (0.111) 1.000	0.961 ± 0.010 1.000 (0.083) 1.000
	*p* value	<0.0001	<0.0001	<0.0001

^1^ From top to bottom within each cell: mean ± standard error of the mean, median (interquartile difference), mode.

**Table 4:** Comparison of results of categorization of farms (*n*) by using Support vector machines against the prevalence of subclinical mastitis in a farm.

		Categorization of Farms by Means of the Computational Model		
		Allocation into ‘High’ Prevalence Category	Allocation into ‘Low’ Prevalence Category	Total
Prevalence of subclinical mastitis in farm	≥20.0%	118	19	137
	<20.0%	1	235	236
	Total	119	254	373
	≥25.0%	117	12	129
	<25.0%	2	242	244
	Total	119	254	373
	≥30.0%	80	0	80
	<30.0%	39	254	293
	Total	119	254	373

**Table 5:** The 10 independent variables with the greatest importance in the prediction of the level (i.e., low/high) of prevalence of subclinical mastitis for both low (<25.0%) or high (≥25.0%) level of prevalence of subclinical mastitis, ordered by their impact in that prediction.

Independent Variables
Breed of ewes
Vaccination against staphylococcal mastitis
Management system applied in farm
Administration of antibiotics at the end of the lactation period
Application of measures for mastitis control at the end of the lactation period
Application of reproductive control
Altitude of farm location
Wind speed at farm location
Month of lactation period
Annual precipitation at farm location

## Data Availability

Detailed results associated with this study are presented in the Supplementary Materials.

## Supplementary Materials

The following supporting information can be downloaded at: https://www.mdpi.com/article/10.3390/ani14162295/s1, Table S1: Proportion of ewes among total number of ewes on farms in 111 sheep flocks during a countrywide investigation into subclinical mastitis in Greece; Table S2: Steps taken during the procedure for the development of the computational model to predict prevalence of subclinical mastitis in dairy sheep farms; Table S3: Description of components of 543,948,800 assessments performed during the evaluation for construction of computational model by means of supervised learning; Table S4: Summary of results of measures of quality of applying four supervised learning tools for the classification of 113 records from 111 sheep farms in two categories (according to the level of prevalence of subclinical mastitis found in the farms); for each tool and outcome mean value found overall, considering assessment of all hyperparameters and all different evaluations performed by resampling, shuffling, and *k*-fold methods (*k* = 5); Table S5: Median (interquartile range) prevalence of subclinical mastitis among two clusters of 113 records from 111 sheep farms developed through the application of unsupervised learning tools; Table S6: Differences in independent variables between dairy sheep farm records (*n* = 113) clustered in accord with level of subclinical mastitis; Table S7: Measures of quality in the model created by using the decision trees tool during assessment for classification of sheep farms based on prevalence of subclinical mastitis; Table S8: Measures of quality in the models created by using the k-NN tool during assessment for classification of sheep farms based on prevalence of subclinical mastitis; Table S9: Measures of quality in the models created by using the neural networks tool during assessment for classification of sheep farms based on prevalence of subclinical mastitis; Table S10: Measures of quality in the models created by using the Support vector machines tool during assessment for classification of sheep farms based on prevalence of subclinical mastitis.

## References

[1] Giadinis N.D., Arsenos G., Tsakos P., Psychas V., Dovas C.I., Papadopoulos E., Karatzias H., Fthenakis G.C. (2012). ‘Milk-drop syndrome of ewes’: Investigation of the causes in dairy sheep in Greece. Small Rumin. Res..

[2] European Food Safety Authority (2014). Scientific opinion on the welfare risks related to the farming of sheep for wool, meat and milk production. EFSA J..

[3] Zufferey R., Minnig A., Thomann B., Zwygart S., Keil N., Schüpbach G., Miserez R., Zanolari P., Stucki D. (2021). Animal-based indicators for on-farm welfare assessment in sheep. Animals.

[4] Russell S.J., Norvig P. (2010). Artificial Intelligence a Modern Approach.

[5] Arrieta A.B., Díaz-Rodríguez N., Del Ser J., Bennetot A., Tabik S., Barbado A., Garcia S., Gil-Lopez S., Molina D., Benjamins R. (2020). Explainable artificial intelligence (XAI): Concepts, taxonomies, opportunities and challenges toward responsible AI. Inf. Fusion.

[6] Sarker I.H. (2022). AI-based modeling: Techniques, applications and research issues towards automation, intelligent and smart systems. SN Comp. Sci..

[7] Ahsan M.M., Luna S.A., Siddique Z. (2022). Machine-learning-based disease diagnosis: A comprehensive review. Healthcare.

[8] Ahsan M.M., Siddique Z. (2022). Machine learning-based heart disease diagnosis: A systematic literature review. Artif. Intellig. Med..

[9] Bourganou M.V., Kiouvrekis Y., Chatzopoulos D.C., Zikas S., Katsafadou A.I., Liagka D.V., Vasileiou N.G.C., Fthenakis G.C., Lianou D.T. (2024). Assessment of published papers on the use of machine learning in diagnosis and treatment of mastitis. Information.

[10] Fadul-Pacheco L., Delgado H., Cabrera V.E. (2021). Exploring machine learning algorithms for early prediction of clinical mastitis. Int. Dairy J..

[11] Maciel-Guerra A., Esener N., Giebel K., Lea D., Green M.J., Bradley A.J., Dottorini T. (2021). Prediction of *Streptococcus uberis* clinical mastitis treatment success in dairy herds by means of mass spectrometry and machine-learning. Sci. Rep..

[12] Ebrahimie E., Ebrahimi F., Ebrahimi M., Tomlinson S., Petrovski K.R. (2018). Hierarchical pattern recognition in milking parameters predicts mastitis prevalence. Comp. Electron. Agric..

[13] Pakrashi A., Ryan C., Gueret C., Berry D.P., Corcoran M., Keane M.T., Mac Namee B. (2023). Early detection of subclinical mastitis in lactating dairy cows using cow-level features. J. Dairy Sci..

[14] Parker Gaddis K.L., Cole J.B., Clay J.S., Maltecca C. (2016). Benchmarking dairy herd health status using routinely recorded herd summary data. J. Dairy Sci..

[15] Post C., Rietz C., Büscher W., Müller U. (2020). Using sensor data to detect lameness and mastitis treatment events in dairy cows: A comparison of classification models. Sensors.

[16] Esener N., Maciel-Guerra A., Giebel K., Lea D., Green M.J., Bradley A.J., Dottorini T. (2021). Mass spectrometry and machine learning for the accurate diagnosis of benzylpenicillin and multidrug resistance of *Staphylococcus aureus* in bovine mastitis. PLoS Comput. Biol..

[17] Vasileiou N.G.C., Cripps P.J., Ioannidi K.S., Chatzopoulos D.C., Gougoulis D.A., Sarrou S., Orfanou D.C., Politis A.P., Calvo Gonzalez-Valerio T., Argyros S. (2018). Extensive countrywide field investigation of subclinical mastitis in sheep in Greece. J. Dairy Sci..

[18] Fragkou I.A., Boscos C.M., Fthenakis G.C. (2014). Diagnosis of clinical or subclinical mastitis in ewes. Small Rumin. Res..

[19] Kaskous S., Farschtschi S., Pfaffl M.W. (2023). Physiological aspects of milk somatic cell count in small ruminants—A review. Dairy.

[20] Lianou D.T., Chatziprodromidou I.P., Vasileiou N.G.C., Michael C.K., Mavrogianni V.S., Politis A.P., Kordalis N.G., Billinis C., Giannakopoulos A., Papadopoulos E. (2020). A detailed questionnaire for the evaluation of health management in dairy sheep and goats. Animals.

[21] Pedregosa F., Varoquaux G., Gramfort A., Michel V., Thirion B., Grisel O., Blondel M., Prettenhofer P., Weiss R., Dubourg V. (2011). Scikit-learn: Machine learning in Python. J. Mach. Learn. Res..

[22] McCulloch W.S., Pitts W.H. (1943). A logical calculus of the ideas immanent in nervous activity. Bull. Mathem. Biophys..

[23] Berner J., Grohs P., Kutyniok G., Petersen P., Grohs P., Kutyniok G. (2022). The modern mathematics of deep learning. Mathematical Aspects of Deep Learning.

[24] Michael C.K., Lianou D.T., Vasileiou N.G.C., Mavrogianni V.S., Petinaki E., Fthenakis G.C. (2023). Longitudinal study of subclinical mastitis in sheep in Greece: An investigation into incidence risk, associations with milk quality and risk factors of the infection. Animals.

[25] Lianou D.T., Michael C.K., Vasileiou N.G.C., Petinaki E., Cripps P.J., Tsilipounidaki K., Katsafadou A.I., Politis A.P., Kordalis N.G., Ioannidi K.S. (2021). Extensive countrywide field investigation of somatic cell counts and total bacterial counts in bulk-tank raw milk in sheep flocks in Greece. Foods.

[26] Fthenakis G.C. (2023). Correspondence of somatic cell counts in bulk-tank milk to prevalence of subclinical mastitis in sheep flocks. Animals.

[27] Lundberg S.M., Lee S.I. A unified approach to interpreting model predictions. Proceedings of the 31st International Conference on Neural Information Processing Systems (NIPS’17).

[28] Ebrahimie E., Ebrahimi F., Ebrahimi M., Tomlinson S., Petrovski K.R. (2018). A large-scale study of indicators of sub-clinical mastitis in dairy cattle by attribute weighting analysis of milk composition features: Highlighting the predictive power of lactose and electrical conductivity. J. Dairy Sci..

[29] Lianou D.T., Michael C.K., Fthenakis G.C. (2023). Data on mapping 444 dairy small ruminant farms during a countrywide investigation performed in Greece. Animals.

[30] Gelasakis A.I., Mavrogianni V.S., Petridis I.G., Vasileiou N.G.C., Fthenakis G.C. (2015). Mastitis in sheep—The last 10 years and the future of research. Vet. Microbiol..

[31] Jones J.E.T., Watkins G.H., Martin W.B., Aitken I.D. (2000). Mastitis and contagious agalactia. Diseases of Sheep.

[32] Arteche-Villasol N., Fernández M., Gutiérrez-Expósito D., Pérez V. (2022). Pathology of the mammary gland in sheep and goats. J. Comp. Pathol..

[33] Conington J., Cao G., Stott A., Bünger L. (2008). Breeding for resistance to mastitis in United Kingdom sheep, a review and economic appraisal. Vet. Rec..

[34] Oget C., Tosser-Klopp G., Rupp R. (2019). Genetic and genomic studies in ovine mastitis. Small Rumin. Res..

[35] Tassi R., Schiavo M., Filipe J., Todd H., Ewing D., Ballingal K.T. (2021). Intramammary immunisation provides short term protection against *Mannheimia haemolytica* mastitis in sheep. Front. Vet. Sci..

[36] Vasileiou N.G.C., Lianou D.T., Michael C.K., Fthenakis G.C., Mavrogianni V.S. (2022). Vaccination against bacterial mastitis in sheep. Vaccines.

[37] Ruegg P.L. (2011). Mastitis in small ruminants. Am. Assoc. Bov. Pr. Conf. Proc..

[38] Petridis I.G., Fthenakis G.C. (2014). Administration of antibiotics to ewes at the beginning of the dry-period. J. Dairy Res..

[39] Petridis I.G., Fthenakis G.C. (2019). Mammary involution and relevant udder health management in sheep. Small Rumin. Res..

[40] Fthenakis G.C. (1994). Prevalence and aetiology of subclinical mastitis in ewes of southern Greece. Small Rumin. Res..

[41] Bergonier D., de Cremoux R., Rupp R., Lagriffoul G., Berthelot X. (2003). Mastitis of dairy small ruminants. Vet. Res..

[42] Leyshon W.J. (1929). An examination of a number of cases of ovine mastitis. Vet. J..

[43] Arcaro J.R.P., Matarazzo S.V., Pozzi C.R., Arcaro J.I., de Toledo L., Costa E.O., de Miranda M.S. (2013). Effects of environmental modification on mastitis occurrence and hormonal changes in Holstein cows. Pesq. Vet. Brasil..

[44] El-Tarabany M.S., El-Tarabany A.A., Atta M.A. (2017). Physiological and lactation responses of Egyptian dairy Baladi goats to natural thermal stress under subtropical environmental conditions. Int. J. Biometeorol..

[45] Lacetera N., Scalia N., Bernabucci U., Ronchi B., Pirazzi D., Nardone A. (2005). Lymphocyte functions in overconditioned cows around parturition. J. Dairy Sci..

[46] Lecchi C., Rota N., Vitali A., Ceciliani F., Lacetera N. (2016). In vitro assessment of the effects of temperature on phagocytosis, reactive oxygen species production and apoptosis in bovine polymorphonuclear cells. Vet. Immunol. Immunopathol..

[47] Vasileiou N.G.C., Giannakopoulos A., Cripps P.J., Ioannidi K.S., Chatzopoulos D.C., Gougoulis D.A., Billinis C., Mavrogianni V.S., Petinaki E., Fthenakis G.C. (2019). Study of potential environmental factors predisposing ewes to subclinical mastitis in Greece. Comp. Immunol. Microbiol. Inf. Dis..

[48] Bergonier D., Berthelot X. (2003). New advances in epizootiology and control of ewe mastitis. Liv. Prod. Sci..

[49] Kuhn M., Johnson K. (2013). Applied Predictive Modeling.

[50] Ranganathan S., Gribskov M., Nakai K., Schönbach C. (2019). Encyclopedia of Bioinformatics and Computational Biology.

[51] Sullivan R. (2012). Introduction to Data Mining for the Life Sciences.

