# Supplementary material for: The Use of Machine Learning to Predict Prevalence of Subclinical Mastitis in Dairy Sheep Farms
Source: Animals (Basel). 2024 Aug 6;14(16):2295. doi: 10.3390/ani14162295 (PMC11350869; doi:10.3390/ani14162295)
Supplement: Supplementary file 1 [file animals-14-02295-s001.zip › animals-3110453-supplementary.pdf]

The Use of Machine Learning to Predict Prevalence of Subclinical Mastitis in Dairy Sheep Farms

Yiannis Kiouvrekis, Natalia G.C. Vasileiou, Eleni I. Katsarou, Daphne T. Lianou, Charalambia K. Michael, Sotiris Zikas, Angeliki I. Katsafadou, Maria V. Bourganou, Dimitra V. Liagka, Dimitris C. Chatzopoulos, George C. Fthenakis

Table S1. Proportion of ewes among total number of ewes on farms in 111 sheep flocks during a countrywide investigation into subclinical mastitis in Greece.

Farm reference	Total number of ewes on farms (i.e., including primiparae ewes)	Proportion of ewes sampled among total number of ewes on farms
F1	60	33.3%
F2	100	13.0%
F3	70	28.6%
F4	240	8.3%
F5	350	5.7%
F6	200	10.0%
F7	180	11.1%
F8	180	11.1%
F9	170	11.8%
F10	470	4.3%
F11	250	8.0%
F12	310	6.5%
F13	500	4.0%
F14	150	13.3%
F15	240	7.9%
F16	380	5.3%
F17	580	3.4%
F18	800	2.5%
F19	260	7.7%
F20	150	13.3%
F21	600	3.3%
F22	310	6.5%
F23	690	2.9%
F24	430	4.7%
F25	200	10.0%
F26	550	3.6%
F27	170	11.8%
F28	200	10.0%

F29	200	10.0%
F30	280	7.1%
F31	380	5.3%
F32	300	6.7%
F33	150	13.3%
F34	260	7.7%
F35	350	5.7%
F36	150	13.3%
F37	120	16.7%
F38	540	3.7%
F39	100	20.0%
F40	250	8.0%
F41	450	4.4%
F42	250	8.0%
F43	175	10.9%
F44	370	5.4%
F45	320	5.9%
F46	60	33.3%
F47	50	40.0%
F48	270	7.4%
F49	60	33.3%
F50	240	8.3%
F51	40	50.0%
F52	250	8.0%
F53	350	5.7%
F54	600	3.3%
F55	600	3.3%
F56	300	6.7%
F57	130	11.5%
F58	500	4.0%
F59	160	12.5%
F60	170	11.2%
F61	200	10.0%
F62	130	15.4%
F63	130	15.4%
F64	320	6.3%
F65	450	4.4%
F66	300	6.7%
F67	300	6.7%
F68	580	3.4%
F69	380	5.3%
F70	300	6.3%
F71	400	5.0%

F72	320	6.3%
F73	250	8.0%
F74	1000	2.0%
F75	600	3.3%
F76	250	7.2%
F77	500	4.0%
F78	600	3.3%
F79	250	8.0%
F80	120	16.7%
F81	170	11.8%
F82	480	4.2%
F83	1500	1.3%
F84	750	2.7%
F85	160	12.5%
F86	390	5.1%
F87	460	4.3%
F88	370	5.4%
F89	850	2.4%
F90	100	20.0%
F91	400	5.0%
F92	90	22.2%
F93	230	8.3%
F94	250	8.0%
F95	100	19.0%
F96	200	10.0%
F97	700	2.9%
F98	250	8.0%
F99	350	5.7%
F100	400	5.0%
F101	160	12.5%
F102	180	11.1%
F103	600	3.3%
F104	250	8.0%
F105	400	5.0%
F106	800	2.5%
F107	150	13.3%
F108	125	16.0%
F109	120	16.7%
F110	280	7.1%
F111	60	30.8%
Overall	35,925	6.1%<br>median: 7.7% (interquartile range: 6.8%)

Table S2. Steps taken during the procedure for the development of the computational model to predict prevalence of subclinical mastitis in dairy sheep farms.

definition of the problem, (ii) establishment of the desired outcomes, (iii) preparation of the data, (iv) feature scaling, (v) splitting of the data and evaluation of the model and (vi) tuning of hyperparameter

Steps	Specific task within each step
Definition of the problem	This step involved the clear definition of the problem, which the model aimed to solve, in this case, this being a classification task.
Establishment of the desired outcomes	The desired outcomes of the model were specified, in this case, the model aimed to predict the classification of each of 373 records into either class '0' or '1,' which corresponded to the level of prevalence rate of subclinical mastitis in the respective flocks.
Preparation of the data	In order to ensure high-quality data for analysis, a comprehensive data preprocessing strategy was implemented, to identify outliers and inconsistencies within the dataset. Statistical methods (e.g., z-scores and IQR analysis) were used to identify and handle outliers, thus preventing skewing of the results. Additionally, data curation procedures were performed, in order to systematically address inconsistencies, which guaranteed data consistency during the analysis.
Feature scaling	Standardization (also termed Z-score scaling) was employed to preprocess our data. This involved subtracting the mean of each feature from the data points and then dividing by the standard deviation. The result was transformed data with a mean of 0 and a standard deviation of 1. This method was selected for two key reasons: first, standardization improved the performance of machine learning algorithms that relied on distance calculations, for example as K-nearest neighbors and Support vector machines, because it ensured that all features contribute equally to the distance metric preventing features with larger scales from dominating the calculations and second, standardization accelerated the convergence of gradient descent optimization algorithms.
Splitting of the data and evaluation of the model	In order to identify the most effective interpolation method for the study dataset, we implemented a two-pronged approach, utilizing both random shuffling and 5-fold cross-validation; this approach aimed to achieve a robust and unbiased evaluation. First, the entire dataset was randomly shuffled; this mitigated any potential biases that might have arisen through the original order of the data points. Second, a 5-fold cross-validation process was employed, during which the shuffled data was split into five equal folds. In each of ten iterations (one for each fold as the validation set), four folds were combined for training, while the remaining fold served as the validation set. By combining these techniques, a total of 50 unique training-validation set pairs was generated, for a comprehensive evaluation of different interpolation methods.

Tuning of hyperparameters	Hyperparameter tuning for different machine learning models, e.g., K-nearest neighbours, Support vector machines, Neural networks, Decision trees, involved the selection of optimal values for various model-specific parameters to enhance the performance of the models. Each model includes distinct hyperparameters that significantly influence their behaviour and outcomes.<br>For K-nearest neighbours, hyperparameter tuning primarily involved determining the optimal number of neighbours (k) and the distance metric (e.g., Euclidean, Manhattan).<br>In Support vector machines, crucial hyperparameters included the regularization parameter (C), the kernel type (e.g., linear, polynomial, RBF) and the kernel-specific parameters (e.g., degree for polynomial, gamma for RBF).<br>For Neural networks, the tuning process was more complex, due to the larger number of hyperparameters. Key parameters referred to the learning rate, the number of hidden layers and neurons per layer, the activation functions (e.g., ReLU, sigmoid), the batch size, and the number of training epochs.<br>Decision trees involved tuning hyperparameters, such as the maximum depth of the tree, the minimum samples required to split a node, the minimum samples required at a leaf node and the criterion for splitting.

Table S3. Description of components of 543,948,800 assessments performed during the evaluation for construction of computational model by means of supervised learning.

The number of 543,948,800 assessments derived as below.
(1) In total, (1+10+60+12 =) 83 different models were produced during the assessment for classification of records from sheep farms based on level of prevalence of subclinical mastitis (Table 2).
(2) For each model, 50 different datasets were used, to evaluate the accuracy performance and thus 83 (models) × 50 (datasets per model) = 4150 different calculations were made.
(3) This number of calculations referred to only one combination for the 17 variables used in the construction of the model
(4) In total, there were 2 ^ 17 = 131,072 possible combinations for these 17 variables.
(5) The calculations for all models and for all datasets were made across all the 131,072 possible combinations of the 17 variables and, hence, 4150 × 131,072 = 543,948,800 assessments performed in total.

Table S4. Summary of results of measures of quality of applying four supervised learning tools for the classification of 113 records from 111 sheep farms in two categories (according to the level of prevalence of subclinical mastitis found in the farms); for each tool and outcome mean value found overall, considering assessment of all hyperparameters and all different evaluations performed by resampling, shuffling and *k*-fold methods (*k* = 5) [1].

	Measures of quality		
Supervised learning tool	Mean accuracy	Mean accuracy	Mean accuracy
Decision trees	50.4%	45.6%	53.3%
k-NN	48.6%	49.7%	53.5%
Neural networks	48.7%	43.4%	51.4%
Support vector machines	50.3%	51.4%	52.9%

[1] In total, 543,948,800 assessments were made during this evaluation.

Table S5. Median (interquartile range) prevalence of subclinical mastitis among two clusters of 113 records from 111 sheep farms, developed through the application of unsupervised learning tools.

Unsupervised learning tool	Cluster 1	Cluster 2	*p*-value
Affinity propagation	20.0% (20.0%)	28.4% (16.0%)	0.040
Birch_threshold3	25.0% (20.0%)	28.4% (20.5%)	0.91
Birch_threshold4	25.0% (20.0%)	30.0% (22.0%)	0.93
Hierarchical clustering (average, complete, single, ward)	25.0% (20.5%)	20.0% (0.0%)	0.68
K-means	20.0% (15.8%)	30.0% (16.0%)	0.002
Spectral clustering	25.0% (15.0%)	20.5% (24.6%)	0.28
Spectral clustering rbf	25.0% (20.0%)	17.5% (17.5%)	0.58

Table S6. Differences in independent variables between dairy sheep farm records (n = 113) clustered in accord with level of subclinical mastitis in there.

Farms clustered with 'low prevalence'							Farms clustered with 'high prevalence'							*p*-value
Number of ewes in flock (median (interquartile range))														
235 (208)							300 (270)							0.007
Breed of sheep in flock (no.)														
Local	Chios	Karagou-niko	Assaf	Lacaune	Friesian	Friesarta	Local	Chios	Karagou-niko	Assaf	Lacaune	Friesian	Friesarta	
26	8	1	1	6	0	0	11	18	4	2	33	1	2	0.0001
Management system applied in farm (no.)														
Intensive	semi-Intensive	semi-Extensive	Extensive				Intensive	semi-Intensive	semi-Extensive	Extensive				
0	15	21	6				26	43	2	0				< 0.0001
Months after lambing (median (interquartile range))														
1.5 (1.8)							3 (4.5)							0.0007
Application of reproductive control (no.)														
Yes	No						Yes	No						
6	36						24	47						0.023
Application of anti-staphylococcal mastitis vaccination (no.)														
Yes	No						Yes	No						
10	32						19	52						0.73
Milking situation of ewes in the flock (no.)														
Milking	Milking & lamb suckling	Lamb suckling					Milking	Milking & lamb suckling	Lamb suckling					
15	18	9					55	12	4					< 0.0001
Application of post-milking teat dipping (no.)														
Yes	No						Yes	No						
2	40						11	60						0.08

Application of measures at the end of the lactation period (no.)														
Yes			No				Yes			No				
7			35				32			39				0.008
Administration of antibiotics at the start of the dry-period (no.)														
Yes			No				Yes			No				
4			38				11			60				0.37
Minimum temperature of coldest month at farm location during the year prior to the visit (median (interquartile range))														
6.1 (3.3) ^{o}C							1.3 (2.7) ^{o}C							< 0.0001
Annual precipitation at farm location during the year prior to the visit (median (interquartile range))														
662 (210) mm							625 (211) mm							0.24
Annual wind speed at farm location during the year prior to the visit (median (interquartile range))														
4.71 (2.26) m s^{-1}							3.57 (0.96) m s^{-1}							< 0.0001
Altitude above sea level (median (interquartile range))														
98 (150) m							152 (165) m							0.006
Distance from other farms (median (interquartile range))														
325 (196) m							285 (191) m							0.44
Land use at farm location (no.) [1]														
1 [a]	2	3	4	5	6	7	1	2	3	4	5	6	7	
0	5	5	1	1	1	4	1	3	15	1	0	5	5	
8 [a]	9	10	11	12	13	14	8	9	10	11	12	13	14	
3	4	7	3	7	0	1	13	1	16	7	2	1	1	0.07
Microhabitat at farm location (no.) [2]														
1 [a]	2	3	4	5	6	7	1	2	3	4	5	6	7	
0	29	1	0	4	0	7	2	45	2	1	9	3	9	0.59

[1] 1 agroforestry areas, 2 beaches, dunes, sands, 3 complex cultivation patterns, 4 discontinuous urban fabric, 5 fruit trees and berry plantations, 6 land principally occupied by agriculture, with significant areas of natural vegetation, 7 natural grasslands, 8 non-irrigated arable land, 9 olive groves, 10 permanently irrigated land, 11 sclerophyllous vegetation, 12 sea and ocean, 13 transitional woodland-shrub, 14 vineyards.

[2] 1 agroforestry, 2 cultivations, 3 human development, 4 oak forest, 5 pastures, 6 riparian forest, 7 shrubland.

Table S7. Measures of quality in the model created by using the Decision trees tool during assessment for classification of sheep farms based on prevalence of subclinical mastitis.

Set	Accuracy	Precision	Recall
['A', 'B', 'C', 'D', 'E', 'G', 'J', 'K', 'L', 'M', 'T', 'V', 'X', 'Y', 'Z', 'AD', 'AE']	0.6364	0.6667	0.6667
['A', 'B', 'C', 'D', 'E', 'G', 'J', 'K', 'L', 'M', 'T', 'V', 'X', 'Y', 'Z', 'AD', 'AE']	0.8182	0.6667	1.0000
['A', 'B', 'C', 'D', 'E', 'G', 'J', 'K', 'L', 'M', 'T', 'V', 'X', 'Y', 'Z', 'AD', 'AE']	0.6364	0.7143	0.7143
['A', 'B', 'C', 'D', 'E', 'G', 'J', 'K', 'L', 'M', 'T', 'V', 'X', 'Y', 'Z', 'AD', 'AE']	0.8182	0.8000	0.8000
['A', 'B', 'C', 'D', 'E', 'G', 'J', 'K', 'L', 'M', 'T', 'V', 'X', 'Y', 'Z', 'AD', 'AE']	0.8182	0.7500	1.0000
['A', 'B', 'C', 'D', 'E', 'G', 'J', 'K', 'L', 'M', 'T', 'V', 'X', 'Y', 'Z', 'AD', 'AE']	0.7273	0.8333	0.7143
['A', 'B', 'C', 'D', 'E', 'G', 'J', 'K', 'L', 'M', 'T', 'V', 'X', 'Y', 'Z', 'AD', 'AE']	0.7273	0.8333	0.7143
['A', 'B', 'C', 'D', 'E', 'G', 'J', 'K', 'L', 'M', 'T', 'V', 'X', 'Y', 'Z', 'AD', 'AE']	0.6364	0.8333	0.6250
['A', 'B', 'C', 'D', 'E', 'G', 'J', 'K', 'L', 'M', 'T', 'V', 'X', 'Y', 'Z', 'AD', 'AE']	0.9091	0.8000	1.0000
['A', 'B', 'C', 'D', 'E', 'G', 'J', 'K', 'L', 'M', 'T', 'V', 'X', 'Y', 'Z', 'AD', 'AE']	0.7273	0.8571	0.7500
['A', 'B', 'C', 'D', 'E', 'G', 'J', 'K', 'L', 'M', 'T', 'V', 'X', 'Y', 'Z', 'AD', 'AE']	0.8182	0.8571	0.8571
['A', 'B', 'C', 'D', 'E', 'G', 'J', 'K', 'L', 'M', 'T', 'V', 'X', 'Y', 'Z', 'AD', 'AE']	0.9091	1.0000	0.7500
['A', 'B', 'C', 'D', 'E', 'G', 'J', 'K', 'L', 'M', 'T', 'V', 'X', 'Y', 'Z', 'AD', 'AE']	0.6364	0.8571	0.6667

['A', 'B', 'C', 'D', 'E', 'G', 'J', 'K', 'L', 'M', 'T', 'V', 'X', 'Y', 'Z', 'AD', 'AE']	0.8182	1.0000	0.6667
['A', 'B', 'C', 'D', 'E', 'G', 'J', 'K', 'L', 'M', 'T', 'V', 'X', 'Y', 'Z', 'AD', 'AE']	0.8182	0.8750	0.8750
['A', 'B', 'C', 'D', 'E', 'G', 'J', 'K', 'L', 'M', 'T', 'V', 'X', 'Y', 'Z', 'AD', 'AE']	0.9091	0.8571	1.0000
['A', 'B', 'C', 'D', 'E', 'G', 'J', 'K', 'L', 'M', 'T', 'V', 'X', 'Y', 'Z', 'AD', 'AE']	0.9091	0.8571	1.0000
['A', 'B', 'C', 'D', 'E', 'G', 'J', 'K', 'L', 'M', 'T', 'V', 'X', 'Y', 'Z', 'AD', 'AE']	0.7273	0.8750	0.7778
['A', 'B', 'C', 'D', 'E', 'G', 'J', 'K', 'L', 'M', 'T', 'V', 'X', 'Y', 'Z', 'AD', 'AE']	0.7273	0.8750	0.7778
['A', 'B', 'C', 'D', 'E', 'G', 'J', 'K', 'L', 'M', 'T', 'V', 'X', 'Y', 'Z', 'AD', 'AE']	0.9091	0.8750	1.0000
['A', 'B', 'C', 'D', 'E', 'G', 'J', 'K', 'L', 'M', 'T', 'V', 'X', 'Y', 'Z', 'AD', 'AE']	0.9091	0.8889	1.0000
['A', 'B', 'C', 'D', 'E', 'G', 'J', 'K', 'L', 'M', 'T', 'V', 'X', 'Y', 'Z', 'AD', 'AE']	0.9091	0.8889	1.0000
['A', 'B', 'C', 'D', 'E', 'G', 'J', 'K', 'L', 'M', 'T', 'V', 'X', 'Y', 'Z', 'AD', 'AE']	0.9091	0.8889	1.0000
['A', 'B', 'C', 'D', 'E', 'G', 'J', 'K', 'L', 'M', 'T', 'V', 'X', 'Y', 'Z', 'AD', 'AE']	0.9091	1.0000	0.8333
['A', 'B', 'C', 'D', 'E', 'G', 'J', 'K', 'L', 'M', 'T', 'V', 'X', 'Y', 'Z', 'AD', 'AE']	0.9091	1.0000	0.8333
['A', 'B', 'C', 'D', 'E', 'G', 'J', 'K', 'L', 'M', 'T', 'V', 'X', 'Y', 'Z', 'AD', 'AE']	0.9091	1.0000	0.8333
['A', 'B', 'C', 'D', 'E', 'G', 'J', 'K', 'L', 'M', 'T', 'V', 'X', 'Y', 'Z', 'AD', 'AE']	0.9091	1.0000	0.8333

['A', 'B', 'C', 'D', 'E', 'G', 'J', 'K', 'L', 'M', 'T', 'V', 'X', 'Y', 'Z', 'AD', 'AE']	0.8182	1.0000	0.7500
['A', 'B', 'C', 'D', 'E', 'G', 'J', 'K', 'L', 'M', 'T', 'V', 'X', 'Y', 'Z', 'AD', 'AE']	0.9091	1.0000	0.8571
['A', 'B', 'C', 'D', 'E', 'G', 'J', 'K', 'L', 'M', 'T', 'V', 'X', 'Y', 'Z', 'AD', 'AE']	0.9091	1.0000	0.8571
['A', 'B', 'C', 'D', 'E', 'G', 'J', 'K', 'L', 'M', 'T', 'V', 'X', 'Y', 'Z', 'AD', 'AE']	0.9091	1.0000	0.8571
['A', 'B', 'C', 'D', 'E', 'G', 'J', 'K', 'L', 'M', 'T', 'V', 'X', 'Y', 'Z', 'AD', 'AE']	0.8182	1.0000	0.7778
['A', 'B', 'C', 'D', 'E', 'G', 'J', 'K', 'L', 'M', 'T', 'V', 'X', 'Y', 'Z', 'AD', 'AE']	0.9091	1.0000	0.8750
['A', 'B', 'C', 'D', 'E', 'G', 'J', 'K', 'L', 'M', 'T', 'V', 'X', 'Y', 'Z', 'AD', 'AE']	0.9091	1.0000	0.8750
['A', 'B', 'C', 'D', 'E', 'G', 'J', 'K', 'L', 'M', 'T', 'V', 'X', 'Y', 'Z', 'AD', 'AE']	0.9091	1.0000	0.8750
['A', 'B', 'C', 'D', 'E', 'G', 'J', 'K', 'L', 'M', 'T', 'V', 'X', 'Y', 'Z', 'AD', 'AE']	0.9091	1.0000	0.8750
['A', 'B', 'C', 'D', 'E', 'G', 'J', 'K', 'L', 'M', 'T', 'V', 'X', 'Y', 'Z', 'AD', 'AE']	0.9091	1.0000	0.8889
['A', 'B', 'C', 'D', 'E', 'G', 'J', 'K', 'L', 'M', 'T', 'V', 'X', 'Y', 'Z', 'AD', 'AE']	1.0000	1.0000	1.0000
['A', 'B', 'C', 'D', 'E', 'G', 'J', 'K', 'L', 'M', 'T', 'V', 'X', 'Y', 'Z', 'AD', 'AE']	1.0000	1.0000	1.0000
['A', 'B', 'C', 'D', 'E', 'G', 'J', 'K', 'L', 'M', 'T', 'V', 'X', 'Y', 'Z', 'AD', 'AE']	1.0000	1.0000	1.0000
['A', 'B', 'C', 'D', 'E', 'G', 'J', 'K', 'L', 'M', 'T', 'V', 'X', 'Y', 'Z', 'AD', 'AE']	1.0000	1.0000	1.0000

['A', 'B', 'C', 'D', 'E', 'G', 'J', 'K', 'L', 'M', 'T', 'V', 'X', 'Y', 'Z', 'AD', 'AE']	1.0000	1.0000	1.0000
['A', 'B', 'C', 'D', 'E', 'G', 'J', 'K', 'L', 'M', 'T', 'V', 'X', 'Y', 'Z', 'AD', 'AE']	1.0000	1.0000	1.0000
['A', 'B', 'C', 'D', 'E', 'G', 'J', 'K', 'L', 'M', 'T', 'V', 'X', 'Y', 'Z', 'AD', 'AE']	1.0000	1.0000	1.0000
['A', 'B', 'C', 'D', 'E', 'G', 'J', 'K', 'L', 'M', 'T', 'V', 'X', 'Y', 'Z', 'AD', 'AE']	1.0000	1.0000	1.0000
['A', 'B', 'C', 'D', 'E', 'G', 'J', 'K', 'L', 'M', 'T', 'V', 'X', 'Y', 'Z', 'AD', 'AE']	1.0000	1.0000	1.0000
['A', 'B', 'C', 'D', 'E', 'G', 'J', 'K', 'L', 'M', 'T', 'V', 'X', 'Y', 'Z', 'AD', 'AE']	1.0000	1.0000	1.0000
['A', 'B', 'C', 'D', 'E', 'G', 'J', 'K', 'L', 'M', 'T', 'V', 'X', 'Y', 'Z', 'AD', 'AE']	1.0000	1.0000	1.0000
['A', 'B', 'C', 'D', 'E', 'G', 'J', 'K', 'L', 'M', 'T', 'V', 'X', 'Y', 'Z', 'AD', 'AE']	1.0000	1.0000	1.0000
['A', 'B', 'C', 'D', 'E', 'G', 'J', 'K', 'L', 'M', 'T', 'V', 'X', 'Y', 'Z', 'AD', 'AE']	1.0000	1.0000	1.0000

Table S8. Measures of quality in the models created by using the k-NN tool during assessment for classification of sheep farms based on prevalence of subclinical mastitis.

Neighbours	Accuracy	Precision	Recall
1	0.727272727	0.777777778	0.875
1	0.545454545	0.714285714	0.625
1	0.636363636	0.833333333	0.625
1	0.727272727	1	0.625
1	0.818181818	1	0.75
1	0.727272727	0.857142857	0.75
1	0.727272727	0.6	0.75
1	0.545454545	0.625	0.714285714
1	0.727272727	0.857142857	0.75
1	0.818181818	0.875	0.875
1	0.727272727	0.714285714	0.833333333
1	0.636363636	0.666666667	0.666666667
1	0.727272727	0.666666667	0.8
1	0.636363636	0.625	0.833333333
1	0.454545455	0.8	0.444444444
1	0.545454545	0.4	0.5
1	0.909090909	1	0.875
1	0.545454545	0.6	0.5
1	0.636363636	0.5	1
1	0.727272727	0.714285714	0.833333333
1	1	1	1
1	0.363636364	0.25	0.2
1	0.636363636	1	0.555555556
1	0.636363636	0.666666667	0.666666667
1	0.727272727	0.7	1
1	0.818181818	1	0.666666667
1	0.636363636	0.833333333	0.625
1	0.545454545	0.571428571	0.666666667
1	0.454545455	0.625	0.625
1	0.636363636	0.714285714	0.714285714
1	0.727272727	1	0.666666667
1	0.636363636	0.777777778	0.777777778
1	0.636363636	0.857142857	0.666666667
1	0.727272727	0.857142857	0.75
1	0.727272727	0.833333333	0.714285714
1	0.909090909	0.857142857	1
1	0.454545455	0.555555556	0.714285714
1	0.454545455	0.8	0.444444444
1	0.727272727	0.8	0.666666667

1	0.545454545	0.8	0.5
1	0.727272727	0.8	0.666666667
1	0.454545455	0.5	0.333333333
1	0.727272727	0.666666667	0.8
1	0.727272727	0.8	0.666666667
1	0.727272727	0.777777778	0.875
1	0.636363636	0.833333333	0.625
1	0.454545455	0.571428571	0.571428571
1	0.545454545	0.625	0.714285714
1	0.545454545	0.444444444	1
1	0.545454545	0.75	0.666666667
2	0.545454545	1	0.375
2	0.727272727	1	0.625
2	0.636363636	0.833333333	0.625
2	0.363636364	0.6	0.375
2	0.545454545	1	0.375
2	0.545454545	0.8	0.5
2	0.727272727	0.8	0.666666667
2	0.545454545	0.8	0.5
2	0.636363636	0.5	0.25
2	0.454545455	0.6	0.428571429
2	0.454545455	0.75	0.375
2	0.545454545	0.571428571	0.666666667
2	0.363636364	1	0.222222222
2	0.636363636	0.5	0.5
2	0.545454545	0.666666667	0.333333333
2	0.636363636	0.666666667	0.4
2	0.636363636	0.5	0.75
2	0.727272727	1	0.571428571
2	0.636363636	0.75	0.5
2	0.454545455	0.333333333	0.2
2	0.545454545	1	0.444444444
2	0.545454545	1	0.375
2	0.454545455	0.5	0.166666667
2	0.636363636	0.666666667	0.666666667
2	0.272727273	0.5	0.375
2	0.545454545	0.75	0.428571429
2	0.545454545	0.6	0.5
2	0.636363636	0.8	0.571428571
2	0.636363636	1	0.333333333
2	0.454545455	0.75	0.375
2	0.727272727	0.857142857	0.75
2	0.636363636	1	0.428571429

2	0.909090909	1	0.833333333
2	0.454545455	0.6	0.428571429
2	0.545454545	1	0.444444444
2	0.454545455	0.8	0.444444444
2	0.454545455	0.8	0.444444444
2	0.363636364	0.666666667	0.25
2	0.636363636	1	0.333333333
2	0.454545455	0.5	0.166666667
2	0.545454545	0.5	0.2
2	0.272727273	0.666666667	0.222222222
2	0.545454545	0.666666667	0.333333333
2	0.454545455	0.6	0.428571429
2	0.727272727	0.571428571	1
2	0.545454545	1	0.444444444
2	0.545454545	0.666666667	0.333333333
2	0.454545455	0.75	0.375
2	0.454545455	0.75	0.375
2	0.363636364	0.5	0.285714286
3	0.727272727	0.727272727	1
3	0.727272727	0.857142857	0.75
3	0.454545455	0.625	0.625
3	0.636363636	0.75	0.75
3	0.636363636	0.7	0.875
3	0.636363636	0.666666667	0.666666667
3	0.636363636	0.75	0.75
3	0.454545455	0.666666667	0.5
3	0.545454545	0.625	0.714285714
3	0.545454545	0.4	0.5
3	0.636363636	0.7	0.875
3	0.363636364	0.333333333	0.75
3	0.818181818	0.888888889	0.888888889
3	0.545454545	0.555555556	0.833333333
3	0.454545455	0.444444444	0.8
3	0.363636364	0.444444444	0.666666667
3	0.636363636	0.857142857	0.666666667
3	0.454545455	0.4	0.4
3	0.818181818	0.857142857	0.857142857
3	0.636363636	0.666666667	0.666666667
3	0.545454545	0.444444444	1
3	0.454545455	0.5	0.666666667
3	0.636363636	1	0.5
3	0.636363636	0.714285714	0.714285714
3	0.545454545	0.666666667	0.75

3	0.545454545	0.555555556	0.833333333
3	0.727272727	0.777777778	0.875
3	0.636363636	0.666666667	0.666666667
3	0.727272727	0.75	0.857142857
3	0.636363636	0.666666667	0.666666667
3	0.454545455	0.555555556	0.714285714
3	0.909090909	0.857142857	1
3	0.727272727	0.75	0.857142857
3	0.909090909	0.888888889	1
3	0.818181818	0.888888889	0.888888889
3	0.727272727	0.8	0.888888889
3	0.727272727	1	0.666666667
3	0.363636364	0.333333333	0.4
3	0.545454545	0.555555556	0.833333333
3	0.818181818	0.833333333	0.833333333
3	0.545454545	0.714285714	0.625
3	0.636363636	0.666666667	0.666666667
3	0.818181818	0.888888889	0.888888889
3	0.636363636	0.777777778	0.777777778
3	0.636363636	0.5	1
3	0.363636364	0.5	0.428571429
3	0.363636364	0.5	0.428571429
3	0.545454545	0.714285714	0.625
3	0.636363636	0.7	0.875
3	0.727272727	0.714285714	0.833333333
4	0.454545455	0.666666667	0.5
4	0.636363636	0.75	0.5
4	0.454545455	0.625	0.625
4	0.545454545	0.333333333	0.25
4	0.545454545	0.625	0.714285714
4	0.363636364	0.6	0.375
4	0.818181818	1	0.75
4	0.727272727	0.777777778	0.875
4	0.545454545	0.714285714	0.625
4	0.727272727	0.777777778	0.875
4	0.454545455	0.666666667	0.5
4	0.636363636	0.5	1
4	0.818181818	1	0.714285714
4	0.545454545	0.6	0.5
4	0.363636364	0.25	0.2
4	0.545454545	0.833333333	0.555555556
4	0.636363636	1	0.5
4	0.272727273	0.333333333	0.333333333

4	0.727272727	1	0.666666667
4	0.636363636	0.666666667	0.666666667
4	0.363636364	0.333333333	0.75
4	0.363636364	0.428571429	0.5
4	0.454545455	0.428571429	0.6
4	0.727272727	0.857142857	0.75
4	0.727272727	0.833333333	0.714285714
4	0.909090909	1	0.833333333
4	0.363636364	0.5	0.571428571
4	0.636363636	1	0.555555556
4	0.727272727	0.8	0.888888889
4	0.636363636	1	0.555555556
4	0.636363636	0.625	0.833333333
4	0.454545455	0.666666667	0.5
4	0.636363636	0.8	0.571428571
4	0.545454545	0.6	0.5
4	0.545454545	0.666666667	0.333333333
4	0.545454545	0.666666667	0.571428571
4	0.636363636	0.75	0.75
4	0.363636364	0.5	0.428571429
4	0.727272727	0.571428571	1
4	0.363636364	0.666666667	0.444444444
4	0.909090909	1	0.833333333
4	0.545454545	0.714285714	0.625
4	0.545454545	0.8	0.5
4	0.272727273	0.4	0.285714286
4	0.545454545	0.8	0.5
4	0.636363636	0.75	0.5
4	0.454545455	0.4	0.4
4	0.545454545	0.6	0.5
4	0.636363636	0.857142857	0.666666667
4	0.545454545	0.6	0.5
5	0.818181818	0.833333333	0.833333333
5	0.636363636	0.75	0.75
5	0.545454545	0.666666667	0.75
5	0.545454545	0.625	0.714285714
5	0.272727273	0.166666667	0.25
5	0.727272727	0.727272727	1
5	0.636363636	0.7	0.875
5	0.818181818	0.875	0.875
5	0.545454545	0.666666667	0.75
5	0.727272727	0.727272727	1
5	0.636363636	0.75	0.75

5	0.545454545	0.75	0.666666667
5	0.181818182	0.166666667	0.2
5	0.818181818	0.857142857	0.857142857
5	0.545454545	0.6	0.5
5	0.545454545	0.444444444	1
5	0.454545455	0.5	0.666666667
5	0.545454545	0.8	0.5
5	0.454545455	0.4	1
5	0.636363636	0.625	0.833333333
5	0.909090909	1	0.888888889
5	0.454545455	0.444444444	0.8
5	0.363636364	0.444444444	0.666666667
5	0.363636364	0.5	0.571428571
5	0.909090909	0.857142857	1
5	0.727272727	0.75	0.857142857
5	0.818181818	0.8	1
5	0.727272727	0.875	0.777777778
5	0.818181818	0.818181818	1
5	0.727272727	1	0.666666667
5	0.636363636	0.714285714	0.714285714
5	0.454545455	0.625	0.625
5	0.545454545	0.555555556	0.833333333
5	0.727272727	0.777777778	0.875
5	0.545454545	0.625	0.714285714
5	0.363636364	0.4	0.333333333
5	0.636363636	0.625	0.833333333
5	0.454545455	0.714285714	0.555555556
5	0.454545455	0.4	1
5	0.545454545	0.625	0.714285714
5	0.545454545	0.625	0.714285714
5	0.454545455	0.666666667	0.5
5	0.727272727	0.777777778	0.875
5	0.727272727	0.714285714	0.833333333
5	0.454545455	0.5	0.666666667
5	0.454545455	0.428571429	0.6
5	0.454545455	0.5	0.666666667
5	0.545454545	0.666666667	0.75
5	0.454545455	0.5	0.666666667
5	0.727272727	0.8	0.888888889
6	0.454545455	0.625	0.625
6	0.636363636	0.8	0.571428571
6	0.363636364	0.2	0.25
6	0.636363636	0.7	0.875

6	0.727272727	0.8	0.666666667
6	0.636363636	0.75	0.75
6	0.454545455	0.666666667	0.5
6	0.545454545	0.666666667	0.75
6	0.545454545	0.714285714	0.625
6	0.636363636	0.75	0.75
6	0.818181818	1	0.75
6	0.363636364	0.428571429	0.5
6	0.454545455	0.75	0.375
6	0.545454545	0.75	0.666666667
6	0.454545455	0.333333333	0.2
6	0.545454545	0.6	0.5
6	0.727272727	0.833333333	0.714285714
6	0.545454545	0.428571429	0.75
6	0.636363636	0.571428571	0.8
6	0.272727273	0.375	0.5
6	0.545454545	0.444444444	1
6	0.636363636	0.625	0.833333333
6	0.545454545	1	0.444444444
6	0.636363636	0.857142857	0.666666667
6	0.636363636	0.777777778	0.777777778
6	0.636363636	1	0.555555556
6	0.454545455	0.571428571	0.571428571
6	0.818181818	1	0.666666667
6	0.727272727	0.833333333	0.714285714
6	0.636363636	0.75	0.75
6	0.545454545	0.714285714	0.625
6	0.636363636	0.714285714	0.714285714
6	0.454545455	0.5	0.333333333
6	0.545454545	0.571428571	0.666666667
6	0.636363636	0.8	0.571428571
6	0.272727273	0.5	0.375
6	0.727272727	0.714285714	0.833333333
6	0.363636364	0.5	0.428571429
6	0.454545455	0.75	0.375
6	0.636363636	0.833333333	0.625
6	0.727272727	0.714285714	0.833333333
6	0.363636364	0.75	0.333333333
6	0.454545455	0.4	1
6	0.454545455	0.571428571	0.571428571
6	0.363636364	0.428571429	0.5
6	0.636363636	0.857142857	0.666666667
6	0.545454545	0.6	0.5

6	0.545454545	0.5	0.6
6	0.363636364	0.428571429	0.5
6	0.363636364	0.571428571	0.5
7	0.727272727	0.727272727	1
7	0.363636364	0.285714286	0.5
7	0.727272727	0.75	0.857142857
7	0.636363636	0.7	0.875
7	0.636363636	0.75	0.75
7	0.727272727	0.8	0.666666667
7	0.727272727	0.777777778	0.875
7	0.545454545	0.666666667	0.75
7	0.363636364	0.571428571	0.5
7	0.818181818	0.875	0.875
7	0.727272727	0.777777778	0.875
7	0.545454545	0.8	0.5
7	0.454545455	0.5	0.666666667
7	0.454545455	0.375	0.75
7	0.636363636	0.666666667	0.666666667
7	0.909090909	0.875	1
7	0.363636364	0.333333333	0.4
7	0.818181818	0.818181818	1
7	0.454545455	0.5	0.833333333
7	0.636363636	0.555555556	1
7	0.727272727	1	0.666666667
7	0.636363636	0.625	0.833333333
7	0.454545455	0.4	1
7	0.545454545	0.833333333	0.555555556
7	0.727272727	0.8	0.888888889
7	0.636363636	0.777777778	0.777777778
7	0.636363636	0.75	0.75
7	0.909090909	0.875	1
7	0.818181818	1	0.666666667
7	0.454545455	0.555555556	0.714285714
7	0.454545455	0.5	0.666666667
7	0.545454545	0.571428571	0.666666667
7	0.727272727	0.75	0.857142857
7	0.727272727	0.777777778	0.875
7	0.636363636	0.625	0.833333333
7	0.363636364	0.571428571	0.5
7	0.909090909	0.875	1
7	0.727272727	0.666666667	1
7	0.727272727	0.857142857	0.75
7	0.545454545	0.714285714	0.625

7	0.454545455	0.555555556	0.714285714
7	0.545454545	0.625	0.714285714
7	0.454545455	0.4	1
7	0.727272727	0.875	0.777777778
7	0.727272727	0.875	0.777777778
7	0.454545455	0.5	0.833333333
7	0.545454545	0.666666667	0.75
7	0.363636364	0.444444444	0.666666667
7	0.454545455	0.428571429	0.6
7	0.545454545	0.571428571	0.666666667
8	0.636363636	0.857142857	0.666666667
8	0.454545455	0.5	0.666666667
8	0.545454545	0.714285714	0.625
8	0.454545455	0.5	0.666666667
8	0.545454545	0.5	0.6
8	0.454545455	0.5	0.333333333
8	0.545454545	0.571428571	0.666666667
8	0.727272727	0.857142857	0.75
8	0.545454545	0.8	0.5
8	0.272727273	0.428571429	0.428571429
8	0.545454545	0.666666667	0.571428571
8	0.545454545	0.444444444	1
8	0.545454545	0.833333333	0.555555556
8	0.636363636	0.666666667	0.666666667
8	0.454545455	0.5	0.5
8	0.545454545	0.666666667	0.571428571
8	0.727272727	0.777777778	0.875
8	0.545454545	0.571428571	0.666666667
8	0.272727273	0.5	0.375
8	0.636363636	0.8	0.571428571
8	0.454545455	0.8	0.444444444
8	0.727272727	0.8	0.888888889
8	0.636363636	0.857142857	0.666666667
8	0.636363636	0.75	0.75
8	0.909090909	0.875	1
8	0.818181818	1	0.666666667
8	0.181818182	0.333333333	0.285714286
8	0.454545455	0.5	0.666666667
8	0.545454545	0.5	0.8
8	0.545454545	1	0.444444444
8	0.727272727	0.714285714	0.833333333
8	0.545454545	0.444444444	1
8	0.636363636	1	0.5

8	0.363636364	0.428571429	0.5
8	0.545454545	0.428571429	0.75
8	0.909090909	0.875	1
8	0.545454545	0.6	0.5
8	0.454545455	0.4	0.4
8	0.727272727	0.875	0.777777778
8	0.636363636	0.75	0.75
8	0.545454545	0.666666667	0.75
8	0.363636364	0.571428571	0.5
8	0.727272727	0.857142857	0.75
8	0.636363636	0.75	0.75
8	0.636363636	0.7	0.875
8	0.454545455	0.333333333	0.5
8	0.818181818	0.857142857	0.857142857
8	0.545454545	0.714285714	0.625
8	0.454545455	0.666666667	0.5
8	0.727272727	0.8	0.666666667
9	0.454545455	0.5	0.833333333
9	0.636363636	0.777777778	0.777777778
9	0.454545455	0.5	0.666666667
9	0.454545455	0.428571429	0.6
9	0.363636364	0.444444444	0.666666667
9	0.818181818	0.8	1
9	0.454545455	0.555555556	0.714285714
9	0.636363636	0.75	0.75
9	0.636363636	0.75	0.75
9	0.636363636	0.625	0.833333333
9	0.727272727	0.8	0.888888889
9	0.454545455	0.4	1
9	0.636363636	0.714285714	0.714285714
9	0.727272727	0.777777778	0.875
9	0.545454545	0.625	0.714285714
9	0.636363636	0.625	0.833333333
9	0.545454545	0.571428571	0.666666667
9	0.727272727	0.75	0.857142857
9	0.636363636	0.7	0.875
9	0.545454545	0.555555556	0.833333333
9	0.636363636	0.777777778	0.777777778
9	0.727272727	0.8	0.888888889
9	0.636363636	0.857142857	0.666666667
9	0.454545455	0.555555556	0.714285714
9	0.909090909	1	0.833333333
9	0.818181818	0.777777778	1

9	0.545454545	0.666666667	0.75
9	0.545454545	0.5	1
9	0.454545455	0.5	0.833333333
9	0.545454545	0.444444444	1
9	0.636363636	0.625	0.833333333
9	0.636363636	0.857142857	0.666666667
9	0.454545455	0.5	0.666666667
9	0.636363636	0.833333333	0.625
9	0.818181818	0.818181818	1
9	0.363636364	0.333333333	0.4
9	0.909090909	0.875	1
9	0.545454545	0.571428571	0.666666667
9	0.545454545	0.428571429	0.75
9	0.454545455	0.625	0.625
9	0.636363636	0.7	0.875
9	0.727272727	0.777777778	0.875
9	0.727272727	0.777777778	0.875
9	0.727272727	0.777777778	0.875
9	0.545454545	0.666666667	0.75
9	0.818181818	0.857142857	0.857142857
9	0.454545455	0.375	0.75
9	0.636363636	0.7	0.875
9	0.818181818	0.833333333	0.833333333
9	0.636363636	0.75	0.75
10	0.545454545	0.75	0.666666667
10	0.727272727	1	0.666666667
10	0.545454545	0.666666667	0.75
10	0.727272727	0.75	0.857142857
10	0.727272727	0.777777778	0.875
10	0.545454545	0.428571429	0.75
10	0.363636364	0.571428571	0.5
10	0.636363636	0.75	0.75
10	0.727272727	0.777777778	0.875
10	0.727272727	0.8	0.888888889
10	0.636363636	0.625	0.833333333
10	0.545454545	0.8	0.5
10	0.636363636	0.666666667	0.666666667
10	0.545454545	0.666666667	0.75
10	0.545454545	0.75	0.666666667
10	0.818181818	1	0.666666667
10	0.545454545	0.666666667	0.75
10	0.454545455	0.5	0.5
10	0.545454545	0.666666667	0.75

10	0.545454545	0.571428571	0.666666667
10	0.636363636	0.75	0.75
10	0.636363636	0.833333333	0.625
10	0.636363636	1	0.555555556
10	0.818181818	0.857142857	0.857142857
10	0.727272727	0.8	0.666666667
10	0.363636364	0.444444444	0.666666667
10	0.454545455	0.5	0.833333333
10	0.545454545	0.444444444	1
10	0.454545455	0.333333333	0.5
10	0.636363636	0.625	0.833333333
10	0.545454545	0.444444444	1
10	0.727272727	0.857142857	0.75
10	0.636363636	0.75	0.75
10	0.727272727	0.833333333	0.714285714
10	0.363636364	0.444444444	0.666666667
10	0.818181818	0.8	1
10	0.363636364	0.5	0.428571429
10	0.636363636	0.777777778	0.777777778
10	0.545454545	0.5	1
10	0.454545455	0.428571429	0.6
10	0.545454545	0.625	0.714285714
10	0.818181818	0.857142857	0.857142857
10	0.181818182	0.333333333	0.285714286
10	0.636363636	0.857142857	0.666666667
10	0.454545455	0.4	0.4
10	0.454545455	0.5	0.666666667
10	0.545454545	0.8	0.5
10	0.636363636	0.666666667	0.666666667
10	0.545454545	0.571428571	0.666666667
10	0.636363636	0.714285714	0.714285714

Table S9. Measures of quality in the models created by using the Neural networks tool during assessment for classification of sheep farms based on prevalence of subclinical mastitis.

Hidden layer size	Activation function	Solver	Accuracy	Precision	Recall
10	*identity*	*adam*	0.545454545	0.545454545	1
10	*identity*	*adam*	0.363636364	0	0
10	*identity*	*adam*	0.909090909	1	0.857142857
10	*identity*	*adam*	0.727272727	0.777777778	0.875
10	*identity*	*adam*	0.272727273	0	0
10	*identity*	*adam*	0.818181818	0.833333333	0.833333333
10	*identity*	*adam*	0.272727273	0	0
10	*identity*	*adam*	0.545454545	0.625	0.714285714
10	*identity*	*adam*	0.272727273	0	0
10	*identity*	*adam*	0.454545455	0	0
10	*identity*	*adam*	0.363636364	0.4	0.8
10	*identity*	*adam*	0.545454545	0.571428571	0.666666667
10	*identity*	*adam*	0.636363636	0.75	0.75
10	*identity*	*adam*	0.272727273	0.3	0.75
10	*identity*	*adam*	0.727272727	0.727272727	1
10	*identity*	*adam*	0.545454545	0.714285714	0.625
10	*identity*	*adam*	0.909090909	0.888888889	1
10	*identity*	*adam*	0.818181818	0.833333333	0.833333333
10	*identity*	*adam*	0.454545455	0	0
10	*identity*	*adam*	0.636363636	0.7	0.875
10	*identity*	*adam*	0.545454545	0.666666667	0.75
10	*identity*	*adam*	0.727272727	0.8	0.666666667
10	*identity*	*adam*	0.545454545	0.545454545	1
10	*identity*	*adam*	0.636363636	0.857142857	0.666666667
10	*identity*	*adam*	0.363636364	0	0
10	*identity*	*adam*	0.545454545	0.545454545	1
10	*identity*	*adam*	0.727272727	0.714285714	0.833333333
10	*identity*	*adam*	0.818181818	0.818181818	1
10	*identity*	*adam*	0.181818182	0	0
10	*identity*	*adam*	0.636363636	0.75	0.75
10	*identity*	*adam*	0.454545455	0.5	0.666666667
10	*identity*	*adam*	1	1	1
10	*identity*	*adam*	0.545454545	0	0
10	*identity*	*adam*	0.636363636	0.636363636	1
10	*identity*	*adam*	0.818181818	1	0.714285714
10	*identity*	*adam*	0.181818182	0	0
10	*identity*	*adam*	0.545454545	0	0
10	*identity*	*adam*	0.727272727	0.777777778	0.875

10	*identity*	*adam*	0.363636364	0.5	0.428571429
10	*identity*	*adam*	0.363636364	0.363636364	1
10	*identity*	*adam*	0.545454545	0.714285714	0.625
10	*identity*	*adam*	0.545454545	0.714285714	0.625
10	*identity*	*adam*	0.636363636	0.5	0.75
10	*identity*	*adam*	0.818181818	1	0.777777778
10	*identity*	*adam*	0.818181818	0.888888889	0.888888889
10	*identity*	*adam*	0.818181818	0.818181818	1
10	*identity*	*adam*	0.272727273	0	0
10	*identity*	*adam*	0.636363636	0.666666667	0.666666667
10	*identity*	*adam*	0.818181818	1	0.777777778
10	*identity*	*adam*	0.363636364	0.428571429	0.5
10	*identity*	*lbfgs*	1	1	1
10	*identity*	*lbfgs*	0.818181818	0.818181818	1
10	*identity*	*lbfgs*	0.363636364	0.363636364	1
10	*identity*	*lbfgs*	0.636363636	0.636363636	1
10	*identity*	*lbfgs*	0.818181818	0.875	0.875
10	*identity*	*lbfgs*	0.363636364	0.363636364	1
10	*identity*	*lbfgs*	0.454545455	0.5	0.833333333
10	*identity*	*lbfgs*	0.545454545	0.545454545	1
10	*identity*	*lbfgs*	0.454545455	0.444444444	0.8
10	*identity*	*lbfgs*	0.545454545	0.545454545	1
10	*identity*	*lbfgs*	0.727272727	0.875	0.777777778
10	*identity*	*lbfgs*	1	1	1
10	*identity*	*lbfgs*	0.636363636	0.636363636	1
10	*identity*	*lbfgs*	0.636363636	0.857142857	0.666666667
10	*identity*	*lbfgs*	0.727272727	0.727272727	1
10	*identity*	*lbfgs*	0.545454545	0.545454545	1
10	*identity*	*lbfgs*	0.272727273	0.375	0.5
10	*identity*	*lbfgs*	0.636363636	0.777777778	0.777777778
10	*identity*	*lbfgs*	0.454545455	0.454545455	1
10	*identity*	*lbfgs*	0.909090909	1	0.888888889
10	*identity*	*lbfgs*	0.818181818	0.818181818	1
10	*identity*	*lbfgs*	0.727272727	0.727272727	1
10	*identity*	*lbfgs*	0.454545455	0.5	0.666666667
10	*identity*	*lbfgs*	0.636363636	0.636363636	1
10	*identity*	*lbfgs*	0.909090909	0.875	1
10	*identity*	*lbfgs*	0.636363636	0.636363636	1
10	*identity*	*lbfgs*	0.727272727	0.777777778	0.875
10	*identity*	*lbfgs*	1	1	1
10	*identity*	*lbfgs*	0.909090909	0.888888889	1
10	*identity*	*lbfgs*	0.818181818	0.833333333	0.833333333
10	*identity*	*lbfgs*	0.545454545	0.545454545	1

10	*identity*	*lbfgs*	0.363636364	0.363636364	1
10	*identity*	*lbfgs*	0.909090909	1	0.888888889
10	*identity*	*lbfgs*	0.727272727	0.727272727	1
10	*identity*	*lbfgs*	0.545454545	0.545454545	1
10	*identity*	*lbfgs*	0.454545455	0.454545455	1
10	*identity*	*lbfgs*	1	1	1
10	*identity*	*lbfgs*	0.727272727	0.727272727	1
10	*identity*	*lbfgs*	0.545454545	0.545454545	1
10	*identity*	*lbfgs*	0.636363636	0.636363636	1
10	*identity*	*lbfgs*	0.909090909	0.8	1
10	*identity*	*lbfgs*	0.454545455	0.666666667	0.5
10	*identity*	*lbfgs*	0.909090909	1	0.875
10	*identity*	*lbfgs*	0.727272727	0.727272727	1
10	*identity*	*lbfgs*	1	1	1
10	*identity*	*lbfgs*	0.727272727	0.777777778	0.875
10	*identity*	*lbfgs*	0.545454545	0.545454545	1
10	*identity*	*lbfgs*	0.909090909	0.875	1
10	*identity*	*lbfgs*	0.909090909	0.888888889	1
10	*identity*	*lbfgs*	0.727272727	0.727272727	1
10	*identity*	*sgd*	0.454545455	0.8	0.444444444
10	*identity*	*sgd*	0.727272727	0.666666667	1
10	*identity*	*sgd*	0.545454545	0.545454545	1
10	*identity*	*sgd*	0.363636364	0.5	0.571428571
10	*identity*	*sgd*	0.454545455	0.555555556	0.714285714
10	*identity*	*sgd*	0.636363636	0.857142857	0.666666667
10	*identity*	*sgd*	0.818181818	0.875	0.875
10	*identity*	*sgd*	0.454545455	1	0.333333333
10	*identity*	*sgd*	0.636363636	0.714285714	0.714285714
10	*identity*	*sgd*	0.727272727	0.666666667	0.8
10	*identity*	*sgd*	0.272727273	0	0
10	*identity*	*sgd*	0.636363636	0.75	0.75
10	*identity*	*sgd*	0.545454545	0.428571429	0.75
10	*identity*	*sgd*	0.727272727	0.714285714	0.833333333
10	*identity*	*sgd*	0.727272727	0.75	0.857142857
10	*identity*	*sgd*	0.272727273	0.5	0.125
10	*identity*	*sgd*	0.909090909	0.888888889	1
10	*identity*	*sgd*	0.636363636	0.75	0.75
10	*identity*	*sgd*	0.272727273	1	0.111111111
10	*identity*	*sgd*	0.727272727	0.777777778	0.875
10	*identity*	*sgd*	0.727272727	0.8	0.666666667
10	*identity*	*sgd*	0.636363636	0.75	0.75
10	*identity*	*sgd*	0.272727273	0.4	0.285714286
10	*identity*	*sgd*	0.363636364	0.571428571	0.5

10	*identity*	*sgd*	0.636363636	1	0.2
10	*identity*	*sgd*	0.636363636	0.714285714	0.714285714
10	*identity*	*sgd*	0.636363636	0.75	0.5
10	*identity*	*sgd*	0.454545455	0.5	0.5
10	*identity*	*sgd*	0.545454545	0.6	0.5
10	*identity*	*sgd*	0.272727273	0	0
10	*identity*	*sgd*	0.818181818	0.818181818	1
10	*identity*	*sgd*	0.727272727	0.714285714	0.833333333
10	*identity*	*sgd*	0.363636364	0.4	0.333333333
10	*identity*	*sgd*	0.454545455	0.666666667	0.5
10	*identity*	*sgd*	0.727272727	0.8	0.888888889
10	*identity*	*sgd*	0.636363636	0.857142857	0.666666667
10	*identity*	*sgd*	0.727272727	0.75	0.857142857
10	*identity*	*sgd*	0.818181818	0.857142857	0.857142857
10	*identity*	*sgd*	0.909090909	1	0.833333333
10	*identity*	*sgd*	0.545454545	0.8	0.5
10	*identity*	*sgd*	0.454545455	0.5	0.666666667
10	*identity*	*sgd*	0.909090909	1	0.875
10	*identity*	*sgd*	0.636363636	0.5	0.25
10	*identity*	*sgd*	0.636363636	0.75	0.75
10	*identity*	*sgd*	0.818181818	0.75	0.75
10	*identity*	*sgd*	0.636363636	0.7	0.875
10	*identity*	*sgd*	0.545454545	0.4	0.5
10	*identity*	*sgd*	0.454545455	0.454545455	1
10	*identity*	*sgd*	0.909090909	1	0.875
10	*identity*	*sgd*	0.545454545	1	0.375
10	*logistic*	*adam*	0.818181818	0.8	1
10	*logistic*	*adam*	0.545454545	0.545454545	1
10	*logistic*	*adam*	0.545454545	0.545454545	1
10	*logistic*	*adam*	0.636363636	0.7	0.875
10	*logistic*	*adam*	0.545454545	0.545454545	1
10	*logistic*	*adam*	0.636363636	0.666666667	0.857142857
10	*logistic*	*adam*	0.454545455	0.5	0.833333333
10	*logistic*	*adam*	0.818181818	0.818181818	1
10	*logistic*	*adam*	0.727272727	0.727272727	1
10	*logistic*	*adam*	0.363636364	0.363636364	1
10	*logistic*	*adam*	0.727272727	0.727272727	1
10	*logistic*	*adam*	0.545454545	0.545454545	1
10	*logistic*	*adam*	0.636363636	0.7	0.875
10	*logistic*	*adam*	0.454545455	0.625	0.625
10	*logistic*	*adam*	0.727272727	0.777777778	0.875
10	*logistic*	*adam*	0.545454545	1	0.285714286
10	*logistic*	*adam*	0.727272727	0.8	0.666666667

10	*logistic*	*adam*	0.545454545	0.545454545	1
10	*logistic*	*adam*	0.727272727	0.777777778	0.875
10	*logistic*	*adam*	0.727272727	0.666666667	1
10	*logistic*	*adam*	0.454545455	0.454545455	1
10	*logistic*	*adam*	0.636363636	0.636363636	1
10	*logistic*	*adam*	0.545454545	0.75	0.428571429
10	*logistic*	*adam*	0.454545455	0.5	0.833333333
10	*logistic*	*adam*	0.272727273	0	0
10	*logistic*	*adam*	0.727272727	0.727272727	1
10	*logistic*	*adam*	0.545454545	0.555555556	0.833333333
10	*logistic*	*adam*	0.818181818	0.818181818	1
10	*logistic*	*adam*	0.636363636	0.7	0.875
10	*logistic*	*adam*	0.454545455	0.5	0.666666667
10	*logistic*	*adam*	0.818181818	0.818181818	1
10	*logistic*	*adam*	0.454545455	0.375	0.75
10	*logistic*	*adam*	0.727272727	0.727272727	1
10	*logistic*	*adam*	0.727272727	0.727272727	1
10	*logistic*	*adam*	0.545454545	0.6	0.857142857
10	*logistic*	*adam*	0.363636364	0.363636364	1
10	*logistic*	*adam*	0.636363636	0.777777778	0.777777778
10	*logistic*	*adam*	0.818181818	0.818181818	1
10	*logistic*	*adam*	0.636363636	0.636363636	1
10	*logistic*	*adam*	0.727272727	0.7	1
10	*logistic*	*adam*	0.727272727	0.727272727	1
10	*logistic*	*adam*	0.636363636	0.636363636	1
10	*logistic*	*adam*	0.636363636	0.555555556	1
10	*logistic*	*adam*	0.818181818	0.818181818	1
10	*logistic*	*adam*	0.545454545	0.545454545	1
10	*logistic*	*adam*	0.454545455	0.454545455	1
10	*logistic*	*adam*	0.636363636	0.6	1
10	*logistic*	*adam*	0.545454545	0.444444444	1
10	*logistic*	*adam*	0.818181818	0.818181818	1
10	*logistic*	*adam*	0.636363636	0.7	0.875
10	*logistic*	*lbfgs*	0.727272727	0.571428571	1
10	*logistic*	*lbfgs*	0.636363636	0.636363636	1
10	*logistic*	*lbfgs*	0.363636364	0.444444444	0.666666667
10	*logistic*	*lbfgs*	0.818181818	1	0.777777778
10	*logistic*	*lbfgs*	0.818181818	0.75	1
10	*logistic*	*lbfgs*	0.363636364	0.363636364	1
10	*logistic*	*lbfgs*	0.636363636	0.833333333	0.625
10	*logistic*	*lbfgs*	0.727272727	0.625	1
10	*logistic*	*lbfgs*	0.545454545	0.545454545	1
10	*logistic*	*lbfgs*	0.545454545	0.545454545	1

10	*logistic*	*lbfgs*	0.545454545	0.625	0.714285714
10	*logistic*	*lbfgs*	0.636363636	0.857142857	0.666666667
10	*logistic*	*lbfgs*	0.545454545	0.545454545	1
10	*logistic*	*lbfgs*	0.636363636	0.6	1
10	*logistic*	*lbfgs*	0.818181818	1	0.75
10	*logistic*	*lbfgs*	0.818181818	0.818181818	1
10	*logistic*	*lbfgs*	0.636363636	0.777777778	0.777777778
10	*logistic*	*lbfgs*	0.545454545	0.545454545	1
10	*logistic*	*lbfgs*	0.727272727	0.727272727	1
10	*logistic*	*lbfgs*	0.818181818	0.888888889	0.888888889
10	*logistic*	*lbfgs*	0.818181818	0.818181818	1
10	*logistic*	*lbfgs*	0.727272727	0.666666667	0.8
10	*logistic*	*lbfgs*	0.818181818	0.857142857	0.857142857
10	*logistic*	*lbfgs*	0.545454545	0.625	0.714285714
10	*logistic*	*lbfgs*	0.818181818	0.75	1
10	*logistic*	*lbfgs*	0.818181818	0.8	1
10	*logistic*	*lbfgs*	0.636363636	0.666666667	0.857142857
10	*logistic*	*lbfgs*	0.545454545	0.545454545	1
10	*logistic*	*lbfgs*	0.545454545	0.4	0.5
10	*logistic*	*lbfgs*	0.545454545	0.545454545	1
10	*logistic*	*lbfgs*	0.727272727	0.727272727	1
10	*logistic*	*lbfgs*	0.454545455	0.625	0.625
10	*logistic*	*lbfgs*	0.636363636	0.666666667	0.666666667
10	*logistic*	*lbfgs*	0.454545455	0.454545455	1
10	*logistic*	*lbfgs*	0.727272727	0.727272727	1
10	*logistic*	*lbfgs*	0.909090909	0.9	1
10	*logistic*	*lbfgs*	0.727272727	0.777777778	0.875
10	*logistic*	*lbfgs*	0.818181818	0.8	1
10	*logistic*	*lbfgs*	0.636363636	0.636363636	1
10	*logistic*	*lbfgs*	0.727272727	0.8	0.666666667
10	*logistic*	*lbfgs*	0.818181818	0.875	0.875
10	*logistic*	*lbfgs*	0.727272727	0.777777778	0.875
10	*logistic*	*lbfgs*	0.363636364	0.333333333	0.75
10	*logistic*	*lbfgs*	0.727272727	0.727272727	1
10	*logistic*	*lbfgs*	0.727272727	0.75	0.857142857
10	*logistic*	*lbfgs*	0.636363636	0.75	0.75
10	*logistic*	*lbfgs*	0.818181818	0.875	0.875
10	*logistic*	*lbfgs*	0.636363636	0.7	0.875
10	*logistic*	*lbfgs*	0.545454545	0.545454545	1
10	*logistic*	*lbfgs*	0.727272727	0.7	1
10	*logistic*	*sgd*	0.545454545	0.545454545	1
10	*logistic*	*sgd*	0.636363636	0.636363636	1
10	*logistic*	*sgd*	0.818181818	0.8	1

10	*logistic*	*sgd*	0.727272727	0.727272727	1
10	*logistic*	*sgd*	0.727272727	0.727272727	1
10	*logistic*	*sgd*	0.818181818	0.818181818	1
10	*logistic*	*sgd*	0.818181818	0.818181818	1
10	*logistic*	*sgd*	0.636363636	0.636363636	1
10	*logistic*	*sgd*	0.454545455	0.454545455	1
10	*logistic*	*sgd*	0.545454545	0.545454545	1
10	*logistic*	*sgd*	0.727272727	0.727272727	1
10	*logistic*	*sgd*	0.363636364	0.363636364	1
10	*logistic*	*sgd*	0.454545455	0.454545455	1
10	*logistic*	*sgd*	0.636363636	0.636363636	1
10	*logistic*	*sgd*	0.545454545	0.545454545	1
10	*logistic*	*sgd*	0.545454545	0.545454545	1
10	*logistic*	*sgd*	0.545454545	0.545454545	1
10	*logistic*	*sgd*	0.545454545	0.545454545	1
10	*logistic*	*sgd*	0.818181818	0.8	1
10	*logistic*	*sgd*	0.545454545	0.545454545	1
10	*logistic*	*sgd*	0.636363636	0.7	0.875
10	*logistic*	*sgd*	0.636363636	0.636363636	1
10	*logistic*	*sgd*	0.727272727	0.727272727	1
10	*logistic*	*sgd*	0.818181818	0.818181818	1
10	*logistic*	*sgd*	0.545454545	0.545454545	1
10	*logistic*	*sgd*	0.545454545	0.545454545	1
10	*logistic*	*sgd*	0.636363636	0.636363636	1
10	*logistic*	*sgd*	0.636363636	0.636363636	1
10	*logistic*	*sgd*	0.818181818	0.818181818	1
10	*logistic*	*sgd*	0.727272727	0.727272727	1
10	*logistic*	*sgd*	0.363636364	0.363636364	1
10	*logistic*	*sgd*	0.727272727	0.727272727	1
10	*logistic*	*sgd*	0.636363636	0.6	1
10	*logistic*	*sgd*	0.727272727	0.727272727	1
10	*logistic*	*sgd*	0.818181818	0.8	1
10	*logistic*	*sgd*	0.818181818	0.8	1
10	*logistic*	*sgd*	0.363636364	0.363636364	1
10	*logistic*	*sgd*	0.727272727	0.727272727	1
10	*logistic*	*sgd*	0.272727273	0.3	0.75
10	*logistic*	*sgd*	0.454545455	0.454545455	1
10	*logistic*	*sgd*	0.454545455	0.5	0.833333333
10	*logistic*	*sgd*	0.727272727	0.727272727	1
10	*logistic*	*sgd*	0.818181818	0.818181818	1
10	*logistic*	*sgd*	0.545454545	0.545454545	1
10	*logistic*	*sgd*	0.636363636	0.636363636	1
10	*logistic*	*sgd*	0.545454545	0.6	0.857142857

10	*logistic*	*sgd*	0.545454545	0.545454545	1
10	*logistic*	*sgd*	0.727272727	0.727272727	1
10	*logistic*	*sgd*	0.818181818	0.818181818	1
10	*logistic*	*sgd*	0.818181818	0.818181818	1
10	*relu*	*adam*	0.727272727	0.727272727	1
10	*relu*	*adam*	0.545454545	0.545454545	1
10	*relu*	*adam*	0.727272727	0.777777778	0.875
10	*relu*	*adam*	0.818181818	1	0.666666667
10	*relu*	*adam*	0.545454545	0.5	0.8
10	*relu*	*adam*	0.545454545	0.666666667	0.75
10	*relu*	*adam*	0.727272727	0.727272727	1
10	*relu*	*adam*	0.545454545	0.714285714	0.625
10	*relu*	*adam*	0.363636364	0	0
10	*relu*	*adam*	0.727272727	0.833333333	0.714285714
10	*relu*	*adam*	0.636363636	0.666666667	0.666666667
10	*relu*	*adam*	0.454545455	0.714285714	0.555555556
10	*relu*	*adam*	0.636363636	0.636363636	1
10	*relu*	*adam*	0.545454545	0.545454545	1
10	*relu*	*adam*	0.454545455	0.454545455	1
10	*relu*	*adam*	0.909090909	0.888888889	1
10	*relu*	*adam*	0.545454545	0	0
10	*relu*	*adam*	0.545454545	0.666666667	0.75
10	*relu*	*adam*	0.545454545	0.714285714	0.625
10	*relu*	*adam*	0.727272727	0.714285714	0.833333333
10	*relu*	*adam*	0.545454545	0.545454545	1
10	*relu*	*adam*	0.818181818	1	0.75
10	*relu*	*adam*	0.636363636	0.625	0.833333333
10	*relu*	*adam*	0.727272727	0.857142857	0.75
10	*relu*	*adam*	0.363636364	0.444444444	0.666666667
10	*relu*	*adam*	0.636363636	0.636363636	1
10	*relu*	*adam*	0.818181818	1	0.75
10	*relu*	*adam*	0.636363636	0.636363636	1
10	*relu*	*adam*	0.727272727	0.8	0.888888889
10	*relu*	*adam*	0.454545455	0.5	0.5
10	*relu*	*adam*	0.363636364	0.428571429	0.5
10	*relu*	*adam*	0.545454545	0.6	0.857142857
10	*relu*	*adam*	0.818181818	0.818181818	1
10	*relu*	*adam*	0.818181818	0.666666667	1
10	*relu*	*adam*	0.545454545	0.545454545	1
10	*relu*	*adam*	0.818181818	0.818181818	1
10	*relu*	*adam*	0.727272727	0.727272727	1
10	*relu*	*adam*	0.545454545	0.75	0.666666667
10	*relu*	*adam*	0.909090909	0.888888889	1

10	*relu*	*adam*	0.272727273	0	0
10	*relu*	*adam*	0.363636364	0	0
10	*relu*	*adam*	0.454545455	0.8	0.444444444
10	*relu*	*adam*	0.818181818	0.777777778	1
10	*relu*	*adam*	0.272727273	0	0
10	*relu*	*adam*	0.636363636	0.777777778	0.777777778
10	*relu*	*adam*	0.545454545	0.545454545	1
10	*relu*	*adam*	0.181818182	0.222222222	0.5
10	*relu*	*adam*	0.545454545	0.4	0.5
10	*relu*	*adam*	0.727272727	0.571428571	1
10	*relu*	*adam*	0.818181818	1	0.666666667
10	*relu*	*lbfgs*	0.727272727	0.727272727	1
10	*relu*	*lbfgs*	0.818181818	0.875	0.875
10	*relu*	*lbfgs*	0.727272727	0.777777778	0.875
10	*relu*	*lbfgs*	0.636363636	0.666666667	0.857142857
10	*relu*	*lbfgs*	0.636363636	0.636363636	1
10	*relu*	*lbfgs*	0.727272727	0.714285714	0.833333333
10	*relu*	*lbfgs*	0.909090909	0.875	1
10	*relu*	*lbfgs*	0.454545455	0.454545455	1
10	*relu*	*lbfgs*	0.636363636	0.636363636	1
10	*relu*	*lbfgs*	0.636363636	0.6	1
10	*relu*	*lbfgs*	0.818181818	0.818181818	1
10	*relu*	*lbfgs*	0.727272727	0.727272727	1
10	*relu*	*lbfgs*	0.636363636	0.636363636	1
10	*relu*	*lbfgs*	0.545454545	0.571428571	0.666666667
10	*relu*	*lbfgs*	0.909090909	0.888888889	1
10	*relu*	*lbfgs*	0.545454545	0.545454545	1
10	*relu*	*lbfgs*	0.727272727	0.727272727	1
10	*relu*	*lbfgs*	0.636363636	0.636363636	1
10	*relu*	*lbfgs*	0.545454545	0.545454545	1
10	*relu*	*lbfgs*	0.636363636	0.777777778	0.777777778
10	*relu*	*lbfgs*	0.545454545	0.545454545	1
10	*relu*	*lbfgs*	0.636363636	0.6	1
10	*relu*	*lbfgs*	0.818181818	0.818181818	1
10	*relu*	*lbfgs*	0.909090909	0.9	1
10	*relu*	*lbfgs*	0.545454545	0.545454545	1
10	*relu*	*lbfgs*	0.545454545	0.545454545	1
10	*relu*	*lbfgs*	0.818181818	0.818181818	1
10	*relu*	*lbfgs*	0.545454545	0.666666667	0.75
10	*relu*	*lbfgs*	0.363636364	0.333333333	0.75
10	*relu*	*lbfgs*	0.636363636	0.777777778	0.777777778
10	*relu*	*lbfgs*	0.727272727	0.727272727	1
10	*relu*	*lbfgs*	0.818181818	0.857142857	0.857142857

10	*relu*	*lbfgs*	0.727272727	0.727272727	1
10	*relu*	*lbfgs*	0.545454545	0.571428571	0.666666667
10	*relu*	*lbfgs*	0.363636364	0.333333333	0.75
10	*relu*	*lbfgs*	0.909090909	1	0.875
10	*relu*	*lbfgs*	0.636363636	0.777777778	0.777777778
10	*relu*	*lbfgs*	0.909090909	0.888888889	1
10	*relu*	*lbfgs*	0.727272727	0.714285714	0.833333333
10	*relu*	*lbfgs*	0.727272727	0.727272727	1
10	*relu*	*lbfgs*	1	1	1
10	*relu*	*lbfgs*	0.727272727	0.857142857	0.75
10	*relu*	*lbfgs*	0.636363636	0.625	0.833333333
10	*relu*	*lbfgs*	0.454545455	0.454545455	1
10	*relu*	*lbfgs*	0.818181818	0.875	0.875
10	*relu*	*lbfgs*	0.727272727	0.727272727	1
10	*relu*	*lbfgs*	0.909090909	0.8	1
10	*relu*	*lbfgs*	0.454545455	0.454545455	1
10	*relu*	*lbfgs*	0.545454545	0.6	0.857142857
10	*relu*	*lbfgs*	0.636363636	0.6	1
	relu	*sgd*	0.272727273	0	0
10	*relu*	*sgd*	0.454545455	0	0
10	*relu*	*sgd*	0.818181818	0.818181818	1
10	*relu*	*sgd*	0.727272727	0.727272727	1
10	*relu*	*sgd*	0.727272727	0.727272727	1
10	*relu*	*sgd*	0.272727273	1	0.111111111
10	*relu*	*sgd*	0.818181818	0.818181818	1
10	*relu*	*sgd*	0.727272727	0.727272727	1
10	*relu*	*sgd*	0.454545455	0	0
10	*relu*	*sgd*	0.454545455	0	0
10	*relu*	*sgd*	0.545454545	0.545454545	1
10	*relu*	*sgd*	0.545454545	0.545454545	1
10	*relu*	*sgd*	0.727272727	0.727272727	1
10	*relu*	*sgd*	0.454545455	0	0
10	*relu*	*sgd*	0.727272727	0.727272727	1
10	*relu*	*sgd*	0.363636364	0	0
10	*relu*	*sgd*	0.545454545	0.545454545	1
10	*relu*	*sgd*	0.272727273	0	0
10	*relu*	*sgd*	0.636363636	0.636363636	1
10	*relu*	*sgd*	0.363636364	0	0
10	*relu*	*sgd*	0.272727273	0	0
10	*relu*	*sgd*	0.363636364	0	0
10	*relu*	*sgd*	0.727272727	0.8	0.888888889
10	*relu*	*sgd*	0.454545455	0.454545455	1
10	*relu*	*sgd*	0.727272727	0.727272727	1

10	*relu*	*sgd*	0.454545455	0	0
10	*relu*	*sgd*	0.363636364	0.363636364	1
10	*relu*	*sgd*	0.272727273	0	0
10	*relu*	*sgd*	0.727272727	0.727272727	1
10	*relu*	*sgd*	0.454545455	0.454545455	1
10	*relu*	*sgd*	0.454545455	0	0
10	*relu*	*sgd*	0.545454545	0.545454545	1
10	*relu*	*sgd*	0.545454545	1	0.166666667
10	*relu*	*sgd*	0.636363636	0.777777778	0.777777778
10	*relu*	*sgd*	0.363636364	0	0
10	*relu*	*sgd*	0.818181818	0.8	1
10	*relu*	*sgd*	0.636363636	0	0
10	*relu*	*sgd*	0.545454545	0.545454545	1
10	*relu*	*sgd*	0.272727273	0	0
10	*relu*	*sgd*	0.272727273	0	0
10	*relu*	*sgd*	0.636363636	0	0
10	*relu*	*sgd*	0.181818182	0	0
10	*relu*	*sgd*	0.272727273	0	0
10	*relu*	*sgd*	0.636363636	0.7	0.875
10	*relu*	*sgd*	0.363636364	0.363636364	1
10	*relu*	*sgd*	0.636363636	0.636363636	1
10	*relu*	*sgd*	0.454545455	0.454545455	1
10	*relu*	*sgd*	0.454545455	0	0
10	*relu*	*sgd*	0.272727273	0	0
10	*relu*	*sgd*	0.818181818	0.818181818	1
10	*tanh*	*adam*	0.454545455	0.625	0.625
10	*tanh*	*adam*	0.363636364	0.5	0.142857143
10	*tanh*	*adam*	0.727272727	0.875	0.777777778
10	*tanh*	*adam*	0.545454545	0.625	0.714285714
10	*tanh*	*adam*	0.818181818	0.8	1
10	*tanh*	*adam*	0.818181818	0.818181818	1
10	*tanh*	*adam*	0.818181818	0.8	1
10	*tanh*	*adam*	0.454545455	0.4	1
10	*tanh*	*adam*	0.363636364	0.363636364	1
10	*tanh*	*adam*	0.636363636	0.625	0.833333333
10	*tanh*	*adam*	0.181818182	0.222222222	0.5
10	*tanh*	*adam*	0.727272727	0.8	0.888888889
10	*tanh*	*adam*	0.454545455	0.5	0.833333333
10	*tanh*	*adam*	0.545454545	0.833333333	0.555555556
10	*tanh*	*adam*	0.454545455	0.5	0.5
10	*tanh*	*adam*	0.454545455	0.5	0.833333333
10	*tanh*	*adam*	0.636363636	0.636363636	1
10	*tanh*	*adam*	0.545454545	0.545454545	1

10	*tanh*	*adam*	0.727272727	0.777777778	0.875
10	*tanh*	*adam*	0.454545455	1	0.142857143
10	*tanh*	*adam*	0.727272727	0.727272727	1
10	*tanh*	*adam*	0.272727273	0.5	0.25
10	*tanh*	*adam*	0.454545455	0.375	0.75
10	*tanh*	*adam*	0.545454545	0.6	0.5
10	*tanh*	*adam*	0.818181818	0.888888889	0.888888889
10	*tanh*	*adam*	0.818181818	0.777777778	1
10	*tanh*	*adam*	0.545454545	0.75	0.666666667
10	*tanh*	*adam*	0.727272727	0.727272727	1
10	*tanh*	*adam*	0.454545455	0.444444444	0.8
10	*tanh*	*adam*	0.454545455	0.444444444	0.8
10	*tanh*	*adam*	0.636363636	0.636363636	1
10	*tanh*	*adam*	0.545454545	0.833333333	0.555555556
10	*tanh*	*adam*	0.545454545	0.545454545	1
10	*tanh*	*adam*	0.363636364	0.571428571	0.5
10	*tanh*	*adam*	0.636363636	0.6	1
10	*tanh*	*adam*	0.545454545	0.666666667	0.333333333
10	*tanh*	*adam*	0.545454545	0.545454545	1
10	*tanh*	*adam*	0.727272727	0.777777778	0.875
10	*tanh*	*adam*	0.909090909	0.888888889	1
10	*tanh*	*adam*	0.545454545	0.8	0.5
10	*tanh*	*adam*	0.545454545	0.571428571	0.666666667
10	*tanh*	*adam*	0.454545455	0.454545455	1
10	*tanh*	*adam*	0.727272727	0.727272727	1
10	*tanh*	*adam*	0.454545455	0.625	0.625
10	*tanh*	*adam*	0.909090909	0.857142857	1
10	*tanh*	*adam*	0.818181818	0.777777778	1
10	*tanh*	*adam*	0.727272727	0.666666667	1
10	*tanh*	*adam*	0.818181818	0.8	1
10	*tanh*	*adam*	0.636363636	0.636363636	1
10	*tanh*	*adam*	0.545454545	0.8	0.5
10	*tanh*	*lbfgs*	0.636363636	0.636363636	1
10	*tanh*	*lbfgs*	0.636363636	0.625	0.833333333
10	*tanh*	*lbfgs*	0.727272727	0.75	0.857142857
10	*tanh*	*lbfgs*	0.727272727	0.727272727	1
10	*tanh*	*lbfgs*	0.909090909	0.888888889	1
10	*tanh*	*lbfgs*	0.363636364	0.571428571	0.5
10	*tanh*	*lbfgs*	0.636363636	0.636363636	1
10	*tanh*	*lbfgs*	0.727272727	0.727272727	1
10	*tanh*	*lbfgs*	0.636363636	0.636363636	1
10	*tanh*	*lbfgs*	0.636363636	0.666666667	0.666666667
10	*tanh*	*lbfgs*	0.454545455	0.454545455	1

10	*tanh*	*lbfgs*	0.636363636	0.636363636	1
10	*tanh*	*lbfgs*	0.636363636	0.6	1
10	*tanh*	*lbfgs*	0.909090909	1	0.888888889
10	*tanh*	*lbfgs*	0.727272727	0.714285714	0.833333333
10	*tanh*	*lbfgs*	0.727272727	0.8	0.888888889
10	*tanh*	*lbfgs*	0.727272727	0.8	0.666666667
10	*tanh*	*lbfgs*	0.727272727	0.727272727	1
10	*tanh*	*lbfgs*	0.636363636	0.625	0.833333333
10	*tanh*	*lbfgs*	0.727272727	0.777777778	0.875
10	*tanh*	*lbfgs*	0.727272727	0.7	1
10	*tanh*	*lbfgs*	0.818181818	0.75	1
10	*tanh*	*lbfgs*	0.818181818	0.818181818	1
10	*tanh*	*lbfgs*	0.636363636	0.833333333	0.625
10	*tanh*	*lbfgs*	0.818181818	0.75	1
10	*tanh*	*lbfgs*	0.727272727	0.8	0.888888889
10	*tanh*	*lbfgs*	0.818181818	1	0.777777778
10	*tanh*	*lbfgs*	0.545454545	0.545454545	1
10	*tanh*	*lbfgs*	0.727272727	0.857142857	0.75
10	*tanh*	*lbfgs*	0.636363636	0.636363636	1
10	*tanh*	*lbfgs*	0.363636364	0.363636364	1
10	*tanh*	*lbfgs*	0.818181818	0.818181818	1
10	*tanh*	*lbfgs*	0.727272727	0.727272727	1
10	*tanh*	*lbfgs*	0.818181818	0.818181818	1
10	*tanh*	*lbfgs*	0.727272727	0.777777778	0.875
10	*tanh*	*lbfgs*	0.727272727	0.777777778	0.875
10	*tanh*	*lbfgs*	0.727272727	0.666666667	1
10	*tanh*	*lbfgs*	0.363636364	0.363636364	1
10	*tanh*	*lbfgs*	0.636363636	0.7	0.875
10	*tanh*	*lbfgs*	0.727272727	0.8	0.666666667
10	*tanh*	*lbfgs*	0.454545455	0.454545455	1
10	*tanh*	*lbfgs*	0.545454545	0.555555556	0.833333333
10	*tanh*	*lbfgs*	0.727272727	0.727272727	1
10	*tanh*	*lbfgs*	0.545454545	0.444444444	1
10	*tanh*	*lbfgs*	0.454545455	0.454545455	1
10	*tanh*	*lbfgs*	0.636363636	0.636363636	1
10	*tanh*	*lbfgs*	0.636363636	0.666666667	0.666666667
10	*tanh*	*lbfgs*	0.727272727	0.727272727	1
10	*tanh*	*lbfgs*	0.727272727	0.727272727	1
10	*tanh*	*lbfgs*	0.636363636	0.5	0.75
10	*tanh*	*sgd*	0.363636364	0.4	0.8
10	*tanh*	*sgd*	0.363636364	0.333333333	0.75
10	*tanh*	*sgd*	0.636363636	0.666666667	0.857142857
10	*tanh*	*sgd*	0.363636364	0.666666667	0.444444444

10	*tanh*	*sgd*	0.545454545	0.666666667	0.75
10	*tanh*	*sgd*	0.545454545	0.571428571	0.666666667
10	*tanh*	*sgd*	0.545454545	0.625	0.714285714
10	*tanh*	*sgd*	0.727272727	0.8	0.888888889
10	*tanh*	*sgd*	0.363636364	0.363636364	1
10	*tanh*	*sgd*	0.727272727	0.7	1
10	*tanh*	*sgd*	0.727272727	0.727272727	1
10	*tanh*	*sgd*	0.818181818	0.8	1
10	*tanh*	*sgd*	0.363636364	0.5	0.571428571
10	*tanh*	*sgd*	0.818181818	0.818181818	1
10	*tanh*	*sgd*	0.636363636	0.7	0.875
10	*tanh*	*sgd*	0.545454545	0.545454545	1
10	*tanh*	*sgd*	0.636363636	0.666666667	0.666666667
10	*tanh*	*sgd*	0.272727273	0.3	0.75
10	*tanh*	*sgd*	0.545454545	0.666666667	0.75
10	*tanh*	*sgd*	0.636363636	0.5	1
10	*tanh*	*sgd*	0.818181818	0.8	1
10	*tanh*	*sgd*	0.636363636	0.6	1
10	*tanh*	*sgd*	0.727272727	0.714285714	0.833333333
10	*tanh*	*sgd*	0.727272727	0.75	0.6
10	*tanh*	*sgd*	0.545454545	0.75	0.666666667
10	*tanh*	*sgd*	0.636363636	0.636363636	1
10	*tanh*	*sgd*	0.636363636	0.7	0.875
10	*tanh*	*sgd*	0.636363636	0.7	0.875
10	*tanh*	*sgd*	0.545454545	0.545454545	1
10	*tanh*	*sgd*	0.454545455	0.454545455	1
10	*tanh*	*sgd*	0.545454545	0.555555556	0.833333333
10	*tanh*	*sgd*	0.545454545	0.666666667	0.75
10	*tanh*	*sgd*	0.636363636	0.636363636	1
10	*tanh*	*sgd*	0.727272727	0.7	1
10	*tanh*	*sgd*	0.636363636	0.7	0.875
10	*tanh*	*sgd*	0.454545455	0.555555556	0.714285714
10	*tanh*	*sgd*	0.454545455	0.5	0.833333333
10	*tanh*	*sgd*	0.363636364	0.428571429	0.5
10	*tanh*	*sgd*	0.727272727	0.8	0.888888889
10	*tanh*	*sgd*	0.818181818	0.8	1
10	*tanh*	*sgd*	0.545454545	0.545454545	1
10	*tanh*	*sgd*	0.818181818	0.8	1
10	*tanh*	*sgd*	0.727272727	0.666666667	1
10	*tanh*	*sgd*	0.636363636	0.625	0.833333333
10	*tanh*	*sgd*	0.545454545	0.75	0.666666667
10	*tanh*	*sgd*	0.545454545	0.545454545	1
10	*tanh*	*sgd*	0.727272727	0.727272727	1

10	*tanh*	*sgd*	0.909090909	0.888888889	1
10	*tanh*	*sgd*	0.545454545	0.833333333	0.555555556
10	*tanh*	*sgd*	0.818181818	0.875	0.875
20	*identity*	*adam*	0.727272727	0.8	0.666666667
20	*identity*	*adam*	0.727272727	0.727272727	1
20	*identity*	*adam*	0.545454545	0.6	0.5
20	*identity*	*adam*	0.454545455	0.454545455	1
20	*identity*	*adam*	0.909090909	1	0.875
20	*identity*	*adam*	0.545454545	0.571428571	0.666666667
20	*identity*	*adam*	0.545454545	0.714285714	0.625
20	*identity*	*adam*	0.636363636	0.714285714	0.714285714
20	*identity*	*adam*	0.727272727	0.727272727	1
20	*identity*	*adam*	0.545454545	0.714285714	0.625
20	*identity*	*adam*	0.727272727	0.75	0.857142857
20	*identity*	*adam*	0.545454545	0.6	0.857142857
20	*identity*	*adam*	0.636363636	0.625	0.833333333
20	*identity*	*adam*	0.272727273	0.375	0.5
20	*identity*	*adam*	0.454545455	0.5	0.666666667
20	*identity*	*adam*	0.727272727	0.8	0.888888889
20	*identity*	*adam*	0.909090909	1	0.857142857
20	*identity*	*adam*	0.454545455	0.625	0.625
20	*identity*	*adam*	0.454545455	0.5	0.833333333
20	*identity*	*adam*	0.545454545	0.6	0.5
20	*identity*	*adam*	0.272727273	0	0
20	*identity*	*adam*	1	1	1
20	*identity*	*adam*	0.909090909	0.888888889	1
20	*identity*	*adam*	0.272727273	0.5	0.375
20	*identity*	*adam*	0.272727273	0	0
20	*identity*	*adam*	0.636363636	0	0
20	*identity*	*adam*	0.545454545	0.8	0.5
20	*identity*	*adam*	0.636363636	0.571428571	0.8
20	*identity*	*adam*	0.727272727	1	0.571428571
20	*identity*	*adam*	0.636363636	0.636363636	1
20	*identity*	*adam*	0.909090909	1	0.857142857
20	*identity*	*adam*	0.181818182	0.222222222	0.5
20	*identity*	*adam*	0.454545455	0	0
20	*identity*	*adam*	0.727272727	0.625	1
20	*identity*	*adam*	0.454545455	1	0.333333333
20	*identity*	*adam*	0.636363636	0.75	0.75
20	*identity*	*adam*	0.545454545	0.833333333	0.555555556
20	*identity*	*adam*	0.818181818	0.833333333	0.833333333
20	*identity*	*adam*	0.727272727	0.714285714	0.833333333
20	*identity*	*adam*	0.909090909	1	0.888888889

20	*identity*	*adam*	0.727272727	0.666666667	1
20	*identity*	*adam*	1	1	1
20	*identity*	*adam*	0.818181818	0.818181818	1
20	*identity*	*adam*	0.636363636	0.777777778	0.777777778
20	*identity*	*adam*	0.727272727	0.8	0.888888889
20	*identity*	*adam*	0.272727273	0	0
20	*identity*	*adam*	0.727272727	0.777777778	0.875
20	*identity*	*adam*	0.454545455	0.4	1
20	*identity*	*adam*	0.727272727	0.833333333	0.714285714
20	*identity*	*adam*	0.636363636	0.5	1
20	*identity*	*lbfgs*	1	1	1
20	*identity*	*lbfgs*	0.727272727	0.6	0.75
20	*identity*	*lbfgs*	0.727272727	0.727272727	1
20	*identity*	*lbfgs*	0.454545455	0.555555556	0.714285714
20	*identity*	*lbfgs*	1	1	1
20	*identity*	*lbfgs*	0.818181818	1	0.777777778
20	*identity*	*lbfgs*	0.545454545	0.545454545	1
20	*identity*	*lbfgs*	0.909090909	1	0.857142857
20	*identity*	*lbfgs*	0.545454545	0.545454545	1
20	*identity*	*lbfgs*	0.727272727	0.8	0.888888889
20	*identity*	*lbfgs*	0.727272727	0.666666667	1
20	*identity*	*lbfgs*	0.454545455	0.454545455	1
20	*identity*	*lbfgs*	0.909090909	1	0.833333333
20	*identity*	*lbfgs*	0.818181818	0.818181818	1
20	*identity*	*lbfgs*	1	1	1
20	*identity*	*lbfgs*	0.545454545	0.545454545	1
20	*identity*	*lbfgs*	1	1	1
20	*identity*	*lbfgs*	1	1	1
20	*identity*	*lbfgs*	0.909090909	0.875	1
20	*identity*	*lbfgs*	0.909090909	0.875	1
20	*identity*	*lbfgs*	0.545454545	0.545454545	1
20	*identity*	*lbfgs*	0.727272727	0.727272727	1
20	*identity*	*lbfgs*	0.818181818	0.818181818	1
20	*identity*	*lbfgs*	1	1	1
20	*identity*	*lbfgs*	1	1	1
20	*identity*	*lbfgs*	0.909090909	0.8	1
20	*identity*	*lbfgs*	1	1	1
20	*identity*	*lbfgs*	0.818181818	0.833333333	0.833333333
20	*identity*	*lbfgs*	0.818181818	0.8	1
20	*identity*	*lbfgs*	0.545454545	0.714285714	0.625
20	*identity*	*lbfgs*	0.818181818	0.875	0.875
20	*identity*	*lbfgs*	0.909090909	1	0.857142857
20	*identity*	*lbfgs*	0.454545455	0.666666667	0.5

20	*identity*	*lbfgs*	0.727272727	0.727272727	1
20	*identity*	*lbfgs*	0.454545455	0.454545455	1
20	*identity*	*lbfgs*	0.909090909	1	0.833333333
20	*identity*	*lbfgs*	0.909090909	0.888888889	1
20	*identity*	*lbfgs*	0.909090909	1	0.888888889
20	*identity*	*lbfgs*	0.909090909	1	0.875
20	*identity*	*lbfgs*	0.818181818	1	0.75
20	*identity*	*lbfgs*	0.363636364	0.363636364	1
20	*identity*	*lbfgs*	0.818181818	1	0.714285714
20	*identity*	*lbfgs*	0.545454545	0.545454545	1
20	*identity*	*lbfgs*	1	1	1
20	*identity*	*lbfgs*	0.727272727	0.727272727	1
20	*identity*	*lbfgs*	1	1	1
20	*identity*	*lbfgs*	1	1	1
20	*identity*	*lbfgs*	0.727272727	0.727272727	1
20	*identity*	*lbfgs*	0.636363636	0.636363636	1
20	*identity*	*lbfgs*	0.727272727	0.727272727	1
20	*identity*	*sgd*	0.727272727	0.857142857	0.75
20	*identity*	*sgd*	0.454545455	0.5	0.833333333
20	*identity*	*sgd*	0.818181818	0.875	0.875
20	*identity*	*sgd*	0.363636364	0.5	0.571428571
20	*identity*	*sgd*	0.727272727	1	0.625
20	*identity*	*sgd*	0.727272727	1	0.4
20	*identity*	*sgd*	0.727272727	0.833333333	0.714285714
20	*identity*	*sgd*	0.636363636	0.75	0.5
20	*identity*	*sgd*	0.545454545	0.545454545	1
20	*identity*	*sgd*	0.363636364	0.4	0.333333333
20	*identity*	*sgd*	0.545454545	0.571428571	0.666666667
20	*identity*	*sgd*	0.636363636	0.857142857	0.666666667
20	*identity*	*sgd*	0.454545455	0.6	0.428571429
20	*identity*	*sgd*	0.727272727	0.625	1
20	*identity*	*sgd*	0.636363636	0.625	0.833333333
20	*identity*	*sgd*	0.454545455	0.666666667	0.5
20	*identity*	*sgd*	0.636363636	0.5	0.75
20	*identity*	*sgd*	0.545454545	0.545454545	1
20	*identity*	*sgd*	0.727272727	0.75	0.857142857
20	*identity*	*sgd*	0.818181818	0.8	1
20	*identity*	*sgd*	0.636363636	0.75	0.75
20	*identity*	*sgd*	0.727272727	0.777777778	0.875
20	*identity*	*sgd*	0.727272727	1	0.666666667
20	*identity*	*sgd*	0.363636364	1	0.222222222
20	*identity*	*sgd*	0.727272727	1	0.625
20	*identity*	*sgd*	0.727272727	0.8	0.888888889

20	*identity*	*sgd*	0.636363636	1	0.333333333
20	*identity*	*sgd*	0.545454545	0.545454545	1
20	*identity*	*sgd*	0.636363636	0.714285714	0.714285714
20	*identity*	*sgd*	0.545454545	0.666666667	0.571428571
20	*identity*	*sgd*	0.363636364	0.285714286	0.5
20	*identity*	*sgd*	0.363636364	0.6	0.375
20	*identity*	*sgd*	0.636363636	0.5	0.75
20	*identity*	*sgd*	0.454545455	0.454545455	1
20	*identity*	*sgd*	0.909090909	1	0.875
20	*identity*	*sgd*	0.818181818	1	0.75
20	*identity*	*sgd*	0.545454545	0.555555556	0.833333333
20	*identity*	*sgd*	0.727272727	0.777777778	0.875
20	*identity*	*sgd*	0.545454545	0.444444444	1
20	*identity*	*sgd*	0.727272727	0.777777778	0.875
20	*identity*	*sgd*	0.636363636	0.777777778	0.777777778
20	*identity*	*sgd*	0.272727273	0.6	0.333333333
20	*identity*	*sgd*	0.727272727	0.833333333	0.714285714
20	*identity*	*sgd*	0.181818182	0.333333333	0.285714286
20	*identity*	*sgd*	0.545454545	0.555555556	0.833333333
20	*identity*	*sgd*	0.454545455	1	0.25
20	*identity*	*sgd*	0.636363636	0.777777778	0.777777778
20	*identity*	*sgd*	0.818181818	0.833333333	0.833333333
20	*identity*	*sgd*	0.545454545	0.555555556	0.833333333
20	*identity*	*sgd*	0.727272727	0.857142857	0.75
20	*logistic*	*adam*	0.636363636	0.7	0.875
20	*logistic*	*adam*	0.545454545	0.545454545	1
20	*logistic*	*adam*	0.818181818	0.8	1
20	*logistic*	*adam*	0.363636364	0.363636364	1
20	*logistic*	*adam*	0.909090909	0.888888889	1
20	*logistic*	*adam*	0.727272727	0.7	1
20	*logistic*	*adam*	0.636363636	0.6	1
20	*logistic*	*adam*	0.818181818	0.888888889	0.888888889
20	*logistic*	*adam*	0.545454545	0.545454545	1
20	*logistic*	*adam*	0.636363636	0.6	1
20	*logistic*	*adam*	0.727272727	0.727272727	1
20	*logistic*	*adam*	0.818181818	0.8	1
20	*logistic*	*adam*	0.636363636	0.6	1
20	*logistic*	*adam*	0.727272727	0.727272727	1
20	*logistic*	*adam*	0.727272727	0.727272727	1
20	*logistic*	*adam*	0.545454545	0.571428571	0.666666667
20	*logistic*	*adam*	0.545454545	0.6	0.857142857
20	*logistic*	*adam*	0.636363636	0.666666667	0.857142857
20	*logistic*	*adam*	0.636363636	0.6	1

20	*logistic*	*adam*	0.454545455	0.454545455	1
20	*logistic*	*adam*	0.454545455	0.5	0.833333333
20	*logistic*	*adam*	0.727272727	0.727272727	1
20	*logistic*	*adam*	0.818181818	0.875	0.875
20	*logistic*	*adam*	0.636363636	0.636363636	1
20	*logistic*	*adam*	0.636363636	0.6	1
20	*logistic*	*adam*	0.636363636	0.7	0.875
20	*logistic*	*adam*	0.818181818	0.818181818	1
20	*logistic*	*adam*	0.818181818	0.818181818	1
20	*logistic*	*adam*	0.636363636	0.636363636	1
20	*logistic*	*adam*	0.363636364	0.363636364	1
20	*logistic*	*adam*	0.545454545	0.444444444	1
20	*logistic*	*adam*	0.727272727	0.727272727	1
20	*logistic*	*adam*	0.727272727	0.727272727	1
20	*logistic*	*adam*	1	1	1
20	*logistic*	*adam*	0.545454545	0.545454545	1
20	*logistic*	*adam*	0.545454545	0.666666667	0.75
20	*logistic*	*adam*	0.727272727	0.8	0.888888889
20	*logistic*	*adam*	0.545454545	0.545454545	1
20	*logistic*	*adam*	0.909090909	0.9	1
20	*logistic*	*adam*	0.727272727	0.727272727	1
20	*logistic*	*adam*	0.545454545	0.5	1
20	*logistic*	*adam*	0.363636364	0.363636364	1
20	*logistic*	*adam*	0.727272727	0.666666667	1
20	*logistic*	*adam*	0.545454545	0.545454545	1
20	*logistic*	*adam*	0.818181818	0.888888889	0.888888889
20	*logistic*	*adam*	0.545454545	0.5	0.8
20	*logistic*	*adam*	0.363636364	0.5	0.428571429
20	*logistic*	*adam*	0.727272727	0.727272727	1
20	*logistic*	*adam*	0.636363636	0.636363636	1
20	*logistic*	*adam*	0.727272727	0.7	1
20	*logistic*	*lbfgs*	0.454545455	0.666666667	0.5
20	*logistic*	*lbfgs*	0.454545455	0.4	1
20	*logistic*	*lbfgs*	0.818181818	0.833333333	0.833333333
20	*logistic*	*lbfgs*	0.818181818	0.888888889	0.888888889
20	*logistic*	*lbfgs*	0.909090909	0.857142857	1
20	*logistic*	*lbfgs*	0.818181818	0.857142857	0.857142857
20	*logistic*	*lbfgs*	0.727272727	0.6	0.75
20	*logistic*	*lbfgs*	0.454545455	0.5	0.666666667
20	*logistic*	*lbfgs*	0.545454545	1	0.444444444
20	*logistic*	*lbfgs*	0.727272727	0.7	1
20	*logistic*	*lbfgs*	0.545454545	0.6	0.5
20	*logistic*	*lbfgs*	0.636363636	0.75	0.5

20	*logistic*	*lbfgs*	0.545454545	0.5	1
20	*logistic*	*lbfgs*	0.636363636	0.6	1
20	*logistic*	*lbfgs*	0.818181818	1	0.777777778
20	*logistic*	*lbfgs*	0.818181818	0.818181818	1
20	*logistic*	*lbfgs*	0.636363636	0.75	0.75
20	*logistic*	*lbfgs*	0.545454545	0.555555556	0.833333333
20	*logistic*	*lbfgs*	0.636363636	0.666666667	0.666666667
20	*logistic*	*lbfgs*	0.818181818	0.777777778	1
20	*logistic*	*lbfgs*	0.727272727	0.7	1
20	*logistic*	*lbfgs*	0.363636364	0.375	0.6
20	*logistic*	*lbfgs*	0.818181818	0.818181818	1
20	*logistic*	*lbfgs*	0.545454545	0.75	0.666666667
20	*logistic*	*lbfgs*	0.727272727	0.727272727	1
20	*logistic*	*lbfgs*	0.727272727	0.727272727	1
20	*logistic*	*lbfgs*	0.727272727	0.777777778	0.875
20	*logistic*	*lbfgs*	0.545454545	0.571428571	0.666666667
20	*logistic*	*lbfgs*	0.636363636	0.5	0.75
20	*logistic*	*lbfgs*	0.636363636	0.625	0.833333333
20	*logistic*	*lbfgs*	0.818181818	0.857142857	0.857142857
20	*logistic*	*lbfgs*	0.454545455	0.625	0.625
20	*logistic*	*lbfgs*	0.454545455	0.625	0.625
20	*logistic*	*lbfgs*	0.818181818	0.8	1
20	*logistic*	*lbfgs*	0.818181818	0.818181818	1
20	*logistic*	*lbfgs*	0.818181818	1	0.75
20	*logistic*	*lbfgs*	0.727272727	0.75	0.6
20	*logistic*	*lbfgs*	0.727272727	0.714285714	0.833333333
20	*logistic*	*lbfgs*	0.545454545	0.444444444	1
20	*logistic*	*lbfgs*	0.909090909	0.888888889	1
20	*logistic*	*lbfgs*	0.727272727	0.777777778	0.875
20	*logistic*	*lbfgs*	0.818181818	0.833333333	0.833333333
20	*logistic*	*lbfgs*	0.727272727	0.7	1
20	*logistic*	*lbfgs*	0.454545455	0.6	0.428571429
20	*logistic*	*lbfgs*	0.727272727	0.714285714	0.833333333
20	*logistic*	*lbfgs*	0.454545455	0.666666667	0.5
20	*logistic*	*lbfgs*	0.545454545	0.8	0.5
20	*logistic*	*lbfgs*	0.636363636	0.833333333	0.625
20	*logistic*	*lbfgs*	0.545454545	0.666666667	0.571428571
20	*logistic*	*lbfgs*	0.727272727	0.777777778	0.875
20	*logistic*	*sgd*	0.818181818	0.818181818	1
20	*logistic*	*sgd*	0.727272727	0.727272727	1
20	*logistic*	*sgd*	0.818181818	0.818181818	1
20	*logistic*	*sgd*	0.545454545	0.545454545	1
20	*logistic*	*sgd*	0.545454545	0.545454545	1

20	*logistic*	*sgd*	0.818181818	0.857142857	0.857142857
20	*logistic*	*sgd*	0.636363636	0.636363636	1
20	*logistic*	*sgd*	0.454545455	0.454545455	1
20	*logistic*	*sgd*	0.636363636	0.636363636	1
20	*logistic*	*sgd*	0.545454545	0.545454545	1
20	*logistic*	*sgd*	0.545454545	0.545454545	1
20	*logistic*	*sgd*	0.545454545	0.545454545	1
20	*logistic*	*sgd*	0.636363636	0.6	1
20	*logistic*	*sgd*	0.727272727	0.727272727	1
20	*logistic*	*sgd*	0.545454545	0.545454545	1
20	*logistic*	*sgd*	0.727272727	0.727272727	1
20	*logistic*	*sgd*	0.636363636	0.636363636	1
20	*logistic*	*sgd*	0.818181818	0.8	1
20	*logistic*	*sgd*	0.545454545	0.545454545	1
20	*logistic*	*sgd*	0.636363636	0.636363636	1
20	*logistic*	*sgd*	0.727272727	0.727272727	1
20	*logistic*	*sgd*	0.727272727	0.727272727	1
20	*logistic*	*sgd*	0.636363636	0.7	0.875
20	*logistic*	*sgd*	0.727272727	0.8	0.888888889
20	*logistic*	*sgd*	0.818181818	0.818181818	1
20	*logistic*	*sgd*	0.545454545	0.6	0.857142857
20	*logistic*	*sgd*	0.454545455	0.454545455	1
20	*logistic*	*sgd*	0.545454545	0.545454545	1
20	*logistic*	*sgd*	0.727272727	0.727272727	1
20	*logistic*	*sgd*	0.363636364	0.363636364	1
20	*logistic*	*sgd*	0.636363636	0.636363636	1
20	*logistic*	*sgd*	0.636363636	0.636363636	1
20	*logistic*	*sgd*	0.545454545	0.545454545	1
20	*logistic*	*sgd*	0.727272727	0.727272727	1
20	*logistic*	*sgd*	0.818181818	0.818181818	1
20	*logistic*	*sgd*	0.818181818	0.818181818	1
20	*logistic*	*sgd*	0.545454545	0.545454545	1
20	*logistic*	*sgd*	0.727272727	0.727272727	1
20	*logistic*	*sgd*	0.818181818	0.818181818	1
20	*logistic*	*sgd*	0.545454545	0.545454545	1
20	*logistic*	*sgd*	0.727272727	0.727272727	1
20	*logistic*	*sgd*	0.727272727	0.727272727	1
20	*logistic*	*sgd*	0.363636364	0.363636364	1
20	*logistic*	*sgd*	0.727272727	0.727272727	1
20	*logistic*	*sgd*	0.363636364	0.363636364	1
20	*logistic*	*sgd*	0.454545455	0.454545455	1
20	*logistic*	*sgd*	0.363636364	0.363636364	1
20	*logistic*	*sgd*	0.727272727	0.727272727	1

20	*logistic*	*sgd*	0.545454545	0.545454545	1
20	*logistic*	*sgd*	0.727272727	0.727272727	1
20	*relu*	*adam*	0.545454545	0.545454545	1
20	*relu*	*adam*	0.909090909	1	0.888888889
20	*relu*	*adam*	0.636363636	0.714285714	0.714285714
20	*relu*	*adam*	0.636363636	0.6	0.6
20	*relu*	*adam*	0.272727273	0	0
20	*relu*	*adam*	0.636363636	0.6	0.6
20	*relu*	*adam*	0.454545455	0.5	0.666666667
20	*relu*	*adam*	0.363636364	0.4	0.333333333
20	*relu*	*adam*	0.727272727	0.727272727	1
20	*relu*	*adam*	0.727272727	0.857142857	0.75
20	*relu*	*adam*	0.545454545	0.545454545	1
20	*relu*	*adam*	0.363636364	0.571428571	0.5
20	*relu*	*adam*	0.454545455	0	0
20	*relu*	*adam*	0.909090909	0.888888889	1
20	*relu*	*adam*	0.727272727	1	0.625
20	*relu*	*adam*	0.818181818	0.875	0.875
20	*relu*	*adam*	0.363636364	0.375	0.6
20	*relu*	*adam*	0.545454545	0.571428571	0.666666667
20	*relu*	*adam*	0.909090909	1	0.857142857
20	*relu*	*adam*	0.727272727	0.727272727	1
20	*relu*	*adam*	0.818181818	0.8	1
20	*relu*	*adam*	0.545454545	0.6	0.5
20	*relu*	*adam*	0.363636364	0	0
20	*relu*	*adam*	0.727272727	0.727272727	1
20	*relu*	*adam*	0.545454545	0.714285714	0.625
20	*relu*	*adam*	0.818181818	0.818181818	1
20	*relu*	*adam*	0.818181818	0.875	0.875
20	*relu*	*adam*	0.818181818	0.857142857	0.857142857
20	*relu*	*adam*	0.818181818	0.777777778	1
20	*relu*	*adam*	0.181818182	0	0
20	*relu*	*adam*	0.545454545	0.571428571	0.666666667
20	*relu*	*adam*	0.818181818	0.818181818	1
20	*relu*	*adam*	0.636363636	0.5	1
20	*relu*	*adam*	0.454545455	0.5	0.833333333
20	*relu*	*adam*	0.545454545	0.428571429	0.75
20	*relu*	*adam*	0.454545455	0.333333333	0.5
20	*relu*	*adam*	0.454545455	0.666666667	0.285714286
20	*relu*	*adam*	0.727272727	0.777777778	0.875
20	*relu*	*adam*	0.818181818	0.833333333	0.833333333
20	*relu*	*adam*	0.818181818	1	0.75
20	*relu*	*adam*	0.727272727	0.8	0.666666667

20	*relu*	*adam*	1	1	1
20	*relu*	*adam*	0.727272727	1	0.666666667
20	*relu*	*adam*	0.818181818	1	0.714285714
20	*relu*	*adam*	0.909090909	0.9	1
20	*relu*	*adam*	0.909090909	1	0.857142857
20	*relu*	*adam*	0.818181818	0.875	0.875
20	*relu*	*adam*	0.636363636	1	0.555555556
20	*relu*	*adam*	0.363636364	0.363636364	1
20	*relu*	*adam*	0.363636364	0.4	0.333333333
20	*relu*	*lbfgs*	0.909090909	0.8	1
20	*relu*	*lbfgs*	0.909090909	0.857142857	1
20	*relu*	*lbfgs*	0.818181818	0.875	0.875
20	*relu*	*lbfgs*	1	1	1
20	*relu*	*lbfgs*	0.818181818	0.818181818	1
20	*relu*	*lbfgs*	1	1	1
20	*relu*	*lbfgs*	1	1	1
20	*relu*	*lbfgs*	0.363636364	0.363636364	1
20	*relu*	*lbfgs*	0.454545455	0.6	0.428571429
20	*relu*	*lbfgs*	0.727272727	0.727272727	1
20	*relu*	*lbfgs*	0.454545455	0.625	0.625
20	*relu*	*lbfgs*	0.636363636	0.5	1
20	*relu*	*lbfgs*	0.363636364	0.571428571	0.5
20	*relu*	*lbfgs*	0.545454545	0.545454545	1
20	*relu*	*lbfgs*	0.454545455	0.6	0.428571429
20	*relu*	*lbfgs*	0.454545455	0.454545455	1
20	*relu*	*lbfgs*	0.818181818	0.875	0.875
20	*relu*	*lbfgs*	0.727272727	0.571428571	1
20	*relu*	*lbfgs*	0.636363636	0.625	0.833333333
20	*relu*	*lbfgs*	1	1	1
20	*relu*	*lbfgs*	0.818181818	0.833333333	0.833333333
20	*relu*	*lbfgs*	0.727272727	0.727272727	1
20	*relu*	*lbfgs*	1	1	1
20	*relu*	*lbfgs*	0.545454545	0.545454545	1
20	*relu*	*lbfgs*	0.818181818	0.777777778	1
20	*relu*	*lbfgs*	0.727272727	0.625	1
20	*relu*	*lbfgs*	0.909090909	0.857142857	1
20	*relu*	*lbfgs*	0.909090909	1	0.857142857
20	*relu*	*lbfgs*	0.636363636	0.7	0.875
20	*relu*	*lbfgs*	0.636363636	0.666666667	0.857142857
20	*relu*	*lbfgs*	1	1	1
20	*relu*	*lbfgs*	0.727272727	0.727272727	1
20	*relu*	*lbfgs*	0.727272727	0.727272727	1
20	*relu*	*lbfgs*	0.636363636	0.636363636	1

20	*relu*	*lbfgs*	0.545454545	0.545454545	1
20	*relu*	*lbfgs*	0.636363636	0.636363636	1
20	*relu*	*lbfgs*	0.545454545	0.545454545	1
20	*relu*	*lbfgs*	0.818181818	0.818181818	1
20	*relu*	*lbfgs*	0.818181818	1	0.777777778
20	*relu*	*lbfgs*	0.909090909	1	0.833333333
20	*relu*	*lbfgs*	0.909090909	1	0.875
20	*relu*	*lbfgs*	0.818181818	0.818181818	1
20	*relu*	*lbfgs*	1	1	1
20	*relu*	*lbfgs*	0.818181818	0.857142857	0.857142857
20	*relu*	*lbfgs*	0.545454545	0.545454545	1
20	*relu*	*lbfgs*	0.909090909	0.888888889	1
20	*relu*	*lbfgs*	0.727272727	0.727272727	1
20	*relu*	*lbfgs*	0.818181818	0.833333333	0.833333333
20	*relu*	*lbfgs*	0.818181818	0.888888889	0.888888889
20	*relu*	*lbfgs*	0.909090909	0.857142857	1
20	*relu*	*sgd*	0.454545455	0.5	0.833333333
20	*relu*	*sgd*	0.545454545	0.545454545	1
20	*relu*	*sgd*	0.545454545	0.8	0.5
20	*relu*	*sgd*	0.545454545	0.545454545	1
20	*relu*	*sgd*	0.818181818	0.818181818	1
20	*relu*	*sgd*	0.545454545	0.8	0.5
20	*relu*	*sgd*	0.545454545	0.545454545	1
20	*relu*	*sgd*	0.363636364	0.428571429	0.5
20	*relu*	*sgd*	0.636363636	0.7	0.875
20	*relu*	*sgd*	0.727272727	0.727272727	1
20	*relu*	*sgd*	0.181818182	0	0
20	*relu*	*sgd*	0.727272727	0.727272727	1
20	*relu*	*sgd*	0.545454545	0.545454545	1
20	*relu*	*sgd*	0.818181818	0.818181818	1
20	*relu*	*sgd*	0.454545455	0.454545455	1
20	*relu*	*sgd*	0.727272727	0.727272727	1
20	*relu*	*sgd*	0.545454545	0.555555556	0.833333333
20	*relu*	*sgd*	0.727272727	0.727272727	1
20	*relu*	*sgd*	0.454545455	0.666666667	0.285714286
20	*relu*	*sgd*	0.818181818	0.818181818	1
20	*relu*	*sgd*	0.636363636	0.636363636	1
20	*relu*	*sgd*	0.727272727	0.727272727	1
20	*relu*	*sgd*	0.636363636	0.636363636	1
20	*relu*	*sgd*	0.727272727	0.727272727	1
20	*relu*	*sgd*	0.545454545	0.6	0.857142857
20	*relu*	*sgd*	0.454545455	0	0
20	*relu*	*sgd*	0.363636364	0.363636364	1

20	*relu*	*sgd*	0.545454545	0.555555556	0.833333333
20	*relu*	*sgd*	0.727272727	1	0.5
20	*relu*	*sgd*	0.272727273	1	0.111111111
20	*relu*	*sgd*	0.636363636	0.636363636	1
20	*relu*	*sgd*	0.272727273	0	0
20	*relu*	*sgd*	0.454545455	0.454545455	1
20	*relu*	*sgd*	0.454545455	0	0
20	*relu*	*sgd*	0.545454545	0.545454545	1
20	*relu*	*sgd*	0.727272727	0.727272727	1
20	*relu*	*sgd*	0.363636364	0.363636364	1
20	*relu*	*sgd*	0.727272727	0.727272727	1
20	*relu*	*sgd*	0.636363636	0.6	1
20	*relu*	*sgd*	0.727272727	0.727272727	1
20	*relu*	*sgd*	0.818181818	0.818181818	1
20	*relu*	*sgd*	0.545454545	0.6	0.857142857
20	*relu*	*sgd*	0.636363636	0.5	0.25
20	*relu*	*sgd*	0.545454545	0	0
20	*relu*	*sgd*	0.272727273	0.5	0.125
20	*relu*	*sgd*	0.727272727	0.727272727	1
20	*relu*	*sgd*	0.363636364	0	0
20	*relu*	*sgd*	0.636363636	0.636363636	1
20	*relu*	*sgd*	0.636363636	0	0
20	*relu*	*sgd*	0.818181818	0.818181818	1
20	*tanh*	*adam*	0.545454545	0.6	0.857142857
20	*tanh*	*adam*	0.545454545	0.555555556	0.833333333
20	*tanh*	*adam*	0.545454545	0.545454545	1
20	*tanh*	*adam*	0.545454545	0.833333333	0.555555556
20	*tanh*	*adam*	0.818181818	0.875	0.875
20	*tanh*	*adam*	0.727272727	0.727272727	1
20	*tanh*	*adam*	0.545454545	0.625	0.714285714
20	*tanh*	*adam*	0.636363636	0.625	0.833333333
20	*tanh*	*adam*	0.727272727	0.8	0.888888889
20	*tanh*	*adam*	0.636363636	0.5	1
20	*tanh*	*adam*	0.636363636	0.6	1
20	*tanh*	*adam*	0.545454545	0.714285714	0.625
20	*tanh*	*adam*	0.454545455	0.714285714	0.555555556
20	*tanh*	*adam*	0.545454545	0.6	0.857142857
20	*tanh*	*adam*	0.545454545	0.6	0.857142857
20	*tanh*	*adam*	0.818181818	0.777777778	1
20	*tanh*	*adam*	0.727272727	0.8	0.888888889
20	*tanh*	*adam*	0.636363636	0.7	0.875
20	*tanh*	*adam*	0.454545455	0.625	0.625
20	*tanh*	*adam*	0.818181818	0.818181818	1

20	*tanh*	*adam*	0.545454545	0.714285714	0.625
20	*tanh*	*adam*	0.454545455	0.4	1
20	*tanh*	*adam*	0.272727273	0.25	0.5
20	*tanh*	*adam*	0.727272727	0.714285714	0.833333333
20	*tanh*	*adam*	0.454545455	0.4	1
20	*tanh*	*adam*	0.454545455	0.5	0.833333333
20	*tanh*	*adam*	0.818181818	0.818181818	1
20	*tanh*	*adam*	0.636363636	0.555555556	1
20	*tanh*	*adam*	0.545454545	0.545454545	1
20	*tanh*	*adam*	0.727272727	0.727272727	1
20	*tanh*	*adam*	0.727272727	0.777777778	0.875
20	*tanh*	*adam*	0.272727273	0.375	0.5
20	*tanh*	*adam*	0.636363636	0.75	0.75
20	*tanh*	*adam*	0.545454545	0.571428571	0.666666667
20	*tanh*	*adam*	0.272727273	0.428571429	0.428571429
20	*tanh*	*adam*	1	1	1
20	*tanh*	*adam*	0.818181818	0.777777778	1
20	*tanh*	*adam*	0.545454545	0.666666667	0.75
20	*tanh*	*adam*	0.727272727	0.625	1
20	*tanh*	*adam*	0.363636364	0.4	0.8
20	*tanh*	*adam*	0.818181818	0.8	1
20	*tanh*	*adam*	0.545454545	0.545454545	1
20	*tanh*	*adam*	0.454545455	0.555555556	0.714285714
20	*tanh*	*adam*	0.636363636	0.857142857	0.666666667
20	*tanh*	*adam*	0.454545455	0.5	0.666666667
20	*tanh*	*adam*	0.727272727	0.777777778	0.875
20	*tanh*	*adam*	0.636363636	0.7	0.875
20	*tanh*	*adam*	0.727272727	0.727272727	1
20	*tanh*	*adam*	0.636363636	0.6	1
20	*tanh*	*adam*	0.636363636	0.625	0.833333333
20	*tanh*	*lbfgs*	0.636363636	0.7	0.875
20	*tanh*	*lbfgs*	0.727272727	0.8	0.888888889
20	*tanh*	*lbfgs*	0.545454545	0.714285714	0.625
20	*tanh*	*lbfgs*	0.545454545	0.444444444	1
20	*tanh*	*lbfgs*	0.636363636	0.666666667	0.666666667
20	*tanh*	*lbfgs*	0.454545455	0.75	0.375
20	*tanh*	*lbfgs*	0.363636364	0.5	0.285714286
20	*tanh*	*lbfgs*	0.636363636	0.75	0.75
20	*tanh*	*lbfgs*	0.636363636	1	0.555555556
20	*tanh*	*lbfgs*	0.363636364	0.333333333	0.75
20	*tanh*	*lbfgs*	0.545454545	0.6	0.5
20	*tanh*	*lbfgs*	0.636363636	0.714285714	0.714285714
20	*tanh*	*lbfgs*	0.363636364	0.333333333	0.4

20	*tanh*	*lbfgs*	0.727272727	0.727272727	1
20	*tanh*	*lbfgs*	0.545454545	0.428571429	0.75
20	*tanh*	*lbfgs*	0.909090909	1	0.875
20	*tanh*	*lbfgs*	0.636363636	0.666666667	0.4
20	*tanh*	*lbfgs*	0.727272727	0.714285714	0.833333333
20	*tanh*	*lbfgs*	0.181818182	0.4	0.25
20	*tanh*	*lbfgs*	0.545454545	0.666666667	0.75
20	*tanh*	*lbfgs*	0.545454545	0.444444444	1
20	*tanh*	*lbfgs*	0.272727273	0.375	0.5
20	*tanh*	*lbfgs*	0.636363636	0.6	1
20	*tanh*	*lbfgs*	0.818181818	0.857142857	0.857142857
20	*tanh*	*lbfgs*	0.636363636	0.7	0.875
20	*tanh*	*lbfgs*	0.545454545	0.833333333	0.555555556
20	*tanh*	*lbfgs*	0.818181818	0.75	1
20	*tanh*	*lbfgs*	0.727272727	0.7	1
20	*tanh*	*lbfgs*	0.636363636	0.555555556	1
20	*tanh*	*lbfgs*	0.636363636	0.636363636	1
20	*tanh*	*lbfgs*	0.909090909	1	0.833333333
20	*tanh*	*lbfgs*	0.818181818	0.777777778	1
20	*tanh*	*lbfgs*	0.454545455	0.6	0.428571429
20	*tanh*	*lbfgs*	0.727272727	0.777777778	0.875
20	*tanh*	*lbfgs*	0.727272727	0.727272727	1
20	*tanh*	*lbfgs*	0.818181818	0.8	1
20	*tanh*	*lbfgs*	0.727272727	0.777777778	0.875
20	*tanh*	*lbfgs*	0.818181818	0.818181818	1
20	*tanh*	*lbfgs*	0.545454545	0.6	0.5
20	*tanh*	*lbfgs*	0.272727273	0.375	0.5
20	*tanh*	*lbfgs*	0.818181818	0.818181818	1
20	*tanh*	*lbfgs*	0.636363636	0.777777778	0.777777778
20	*tanh*	*lbfgs*	0.909090909	0.857142857	1
20	*tanh*	*lbfgs*	0.454545455	0.5	0.166666667
20	*tanh*	*lbfgs*	0.727272727	0.8	0.888888889
20	*tanh*	*lbfgs*	0.545454545	0.545454545	1
20	*tanh*	*lbfgs*	0.727272727	0.833333333	0.714285714
20	*tanh*	*lbfgs*	0.545454545	0.555555556	0.833333333
20	*tanh*	*lbfgs*	0.818181818	0.8	1
20	*tanh*	*lbfgs*	0.636363636	0.7	0.875
20	*tanh*	*sgd*	0.454545455	0.625	0.625
20	*tanh*	*sgd*	0.545454545	0.666666667	0.75
20	*tanh*	*sgd*	0.727272727	0.7	1
20	*tanh*	*sgd*	0.636363636	0.777777778	0.777777778
20	*tanh*	*sgd*	0.363636364	0.363636364	1
20	*tanh*	*sgd*	0.727272727	0.75	0.857142857

20	*tanh*	*sgd*	0.636363636	0.777777778	0.777777778
20	*tanh*	*sgd*	0.727272727	0.777777778	0.875
20	*tanh*	*sgd*	0.454545455	0.5	0.5
20	*tanh*	*sgd*	0.454545455	0.454545455	1
20	*tanh*	*sgd*	0.636363636	0.666666667	0.857142857
20	*tanh*	*sgd*	0.363636364	0.363636364	1
20	*tanh*	*sgd*	0.545454545	0.571428571	0.666666667
20	*tanh*	*sgd*	0.636363636	0.6	1
20	*tanh*	*sgd*	0.454545455	0.444444444	0.8
20	*tanh*	*sgd*	0.727272727	0.727272727	1
20	*tanh*	*sgd*	0.909090909	0.888888889	1
20	*tanh*	*sgd*	0.363636364	0.363636364	1
20	*tanh*	*sgd*	0.727272727	0.666666667	1
20	*tanh*	*sgd*	0.454545455	0.4	1
20	*tanh*	*sgd*	0.545454545	0.6	0.857142857
20	*tanh*	*sgd*	0.909090909	0.9	1
20	*tanh*	*sgd*	0.818181818	0.8	1
20	*tanh*	*sgd*	0.545454545	0.545454545	1
20	*tanh*	*sgd*	0.545454545	0.6	0.857142857
20	*tanh*	*sgd*	0.454545455	0.555555556	0.714285714
20	*tanh*	*sgd*	0.727272727	0.727272727	1
20	*tanh*	*sgd*	0.363636364	0.444444444	0.666666667
20	*tanh*	*sgd*	0.727272727	0.727272727	1
20	*tanh*	*sgd*	0.545454545	0.625	0.714285714
20	*tanh*	*sgd*	0.727272727	0.727272727	1
20	*tanh*	*sgd*	0.454545455	0.5	0.833333333
20	*tanh*	*sgd*	0.454545455	0.454545455	1
20	*tanh*	*sgd*	0.727272727	0.8	0.888888889
20	*tanh*	*sgd*	0.727272727	0.7	1
20	*tanh*	*sgd*	0.727272727	0.777777778	0.875
20	*tanh*	*sgd*	0.727272727	0.777777778	0.875
20	*tanh*	*sgd*	0.727272727	0.8	0.888888889
20	*tanh*	*sgd*	0.727272727	0.777777778	0.875
20	*tanh*	*sgd*	0.727272727	0.875	0.777777778
20	*tanh*	*sgd*	0.545454545	0.545454545	1
20	*tanh*	*sgd*	0.909090909	0.888888889	1
20	*tanh*	*sgd*	0.454545455	0.5	0.833333333
20	*tanh*	*sgd*	0.636363636	0.7	0.875
20	*tanh*	*sgd*	0.363636364	0.428571429	0.5
20	*tanh*	*sgd*	0.727272727	0.714285714	0.833333333
20	*tanh*	*sgd*	0.545454545	0.545454545	1
20	*tanh*	*sgd*	0.545454545	0.555555556	0.833333333
20	*tanh*	*sgd*	0.636363636	0.777777778	0.777777778

20	*tanh*	*sgd*	0.727272727	0.857142857	0.75
50	*identity*	*adam*	0.818181818	0.777777778	1
50	*identity*	*adam*	0.727272727	0.833333333	0.714285714
50	*identity*	*adam*	1	1	1
50	*identity*	*adam*	0.636363636	0.636363636	1
50	*identity*	*adam*	0.909090909	0.888888889	1
50	*identity*	*adam*	0.909090909	0.857142857	1
50	*identity*	*adam*	0.636363636	0.857142857	0.666666667
50	*identity*	*adam*	0.909090909	1	0.875
50	*identity*	*adam*	0.727272727	0.875	0.777777778
50	*identity*	*adam*	0.818181818	0.714285714	1
50	*identity*	*adam*	1	1	1
50	*identity*	*adam*	0.636363636	0.75	0.5
50	*identity*	*adam*	0.545454545	0.714285714	0.625
50	*identity*	*adam*	0.636363636	0.857142857	0.666666667
50	*identity*	*adam*	0.636363636	0.6	1
50	*identity*	*adam*	0.181818182	0	0
50	*identity*	*adam*	0.545454545	0.666666667	0.333333333
50	*identity*	*adam*	0.545454545	0.4	0.5
50	*identity*	*adam*	0.636363636	0.636363636	1
50	*identity*	*adam*	0.909090909	0.8	1
50	*identity*	*adam*	1	1	1
50	*identity*	*adam*	1	1	1
50	*identity*	*adam*	0.272727273	0.666666667	0.222222222
50	*identity*	*adam*	1	1	1
50	*identity*	*adam*	0.727272727	0.7	1
50	*identity*	*adam*	0.454545455	0.666666667	0.5
50	*identity*	*adam*	1	1	1
50	*identity*	*adam*	0.454545455	0.666666667	0.5
50	*identity*	*adam*	0.363636364	0.4	0.8
50	*identity*	*adam*	0.454545455	0.5	0.833333333
50	*identity*	*adam*	0.818181818	0.8	1
50	*identity*	*adam*	0.909090909	0.857142857	1
50	*identity*	*adam*	0.909090909	1	0.833333333
50	*identity*	*adam*	0.636363636	0.8	0.571428571
50	*identity*	*adam*	0.818181818	0.777777778	1
50	*identity*	*adam*	0.272727273	0	0
50	*identity*	*adam*	1	1	1
50	*identity*	*adam*	0.636363636	0.7	0.875
50	*identity*	*adam*	0.727272727	0.714285714	0.833333333
50	*identity*	*adam*	1	1	1
50	*identity*	*adam*	0.909090909	0.888888889	1
50	*identity*	*adam*	0.636363636	0.636363636	1

50	*identity*	*adam*	0.545454545	0.833333333	0.555555556
50	*identity*	*adam*	0.909090909	1	0.833333333
50	*identity*	*adam*	0.454545455	0	0
50	*identity*	*adam*	0.909090909	1	0.75
50	*identity*	*adam*	0.909090909	1	0.875
50	*identity*	*adam*	0.818181818	0.8	1
50	*identity*	*adam*	0.454545455	0.5	0.166666667
50	*identity*	*adam*	0.545454545	0.714285714	0.625
50	*identity*	*lbfgs*	0.909090909	0.833333333	1
50	*identity*	*lbfgs*	0.909090909	1	0.833333333
50	*identity*	*lbfgs*	0.909090909	1	0.857142857
50	*identity*	*lbfgs*	1	1	1
50	*identity*	*lbfgs*	0.909090909	1	0.888888889
50	*identity*	*lbfgs*	0.545454545	0.545454545	1
50	*identity*	*lbfgs*	0.909090909	0.875	1
50	*identity*	*lbfgs*	1	1	1
50	*identity*	*lbfgs*	0.909090909	1	0.888888889
50	*identity*	*lbfgs*	1	1	1
50	*identity*	*lbfgs*	1	1	1
50	*identity*	*lbfgs*	0.363636364	0.363636364	1
50	*identity*	*lbfgs*	0.545454545	0.714285714	0.625
50	*identity*	*lbfgs*	0.818181818	0.875	0.875
50	*identity*	*lbfgs*	0.818181818	0.818181818	1
50	*identity*	*lbfgs*	0.818181818	0.818181818	1
50	*identity*	*lbfgs*	0.454545455	0.454545455	1
50	*identity*	*lbfgs*	1	1	1
50	*identity*	*lbfgs*	0.636363636	0.636363636	1
50	*identity*	*lbfgs*	0.545454545	0.545454545	1
50	*identity*	*lbfgs*	0.545454545	0.545454545	1
50	*identity*	*lbfgs*	0.727272727	0.727272727	1
50	*identity*	*lbfgs*	0.909090909	0.9	1
50	*identity*	*lbfgs*	0.818181818	0.818181818	1
50	*identity*	*lbfgs*	1	1	1
50	*identity*	*lbfgs*	0.454545455	0.454545455	1
50	*identity*	*lbfgs*	0.545454545	0.545454545	1
50	*identity*	*lbfgs*	0.909090909	0.888888889	1
50	*identity*	*lbfgs*	0.636363636	0.777777778	0.777777778
50	*identity*	*lbfgs*	0.909090909	0.888888889	1
50	*identity*	*lbfgs*	0.454545455	0.625	0.625
50	*identity*	*lbfgs*	0.818181818	0.8	1
50	*identity*	*lbfgs*	0.909090909	1	0.857142857
50	*identity*	*lbfgs*	0.909090909	0.8	1
50	*identity*	*lbfgs*	1	1	1

50	*identity*	*lbfgs*	0.909090909	0.857142857	1
50	*identity*	*lbfgs*	0.727272727	0.727272727	1
50	*identity*	*lbfgs*	1	1	1
50	*identity*	*lbfgs*	0.727272727	0.727272727	1
50	*identity*	*lbfgs*	1	1	1
50	*identity*	*lbfgs*	1	1	1
50	*identity*	*lbfgs*	0.727272727	0.727272727	1
50	*identity*	*lbfgs*	0.727272727	0.727272727	1
50	*identity*	*lbfgs*	0.636363636	0.636363636	1
50	*identity*	*lbfgs*	1	1	1
50	*identity*	*lbfgs*	0.545454545	0.6	0.857142857
50	*identity*	*lbfgs*	0.545454545	0.545454545	1
50	*identity*	*lbfgs*	0.909090909	1	0.875
50	*identity*	*lbfgs*	1	1	1
50	*identity*	*lbfgs*	0.363636364	0.363636364	1
50	*identity*	*sgd*	0.454545455	0.8	0.444444444
50	*identity*	*sgd*	0.545454545	0.666666667	0.75
50	*identity*	*sgd*	0.545454545	0.833333333	0.555555556
50	*identity*	*sgd*	0.636363636	0.666666667	0.666666667
50	*identity*	*sgd*	0.818181818	0.833333333	0.833333333
50	*identity*	*sgd*	0.636363636	1	0.428571429
50	*identity*	*sgd*	0.636363636	0.636363636	1
50	*identity*	*sgd*	0.727272727	0.75	0.857142857
50	*identity*	*sgd*	0.363636364	0.444444444	0.666666667
50	*identity*	*sgd*	0.636363636	0.571428571	0.8
50	*identity*	*sgd*	0.363636364	0.333333333	0.166666667
50	*identity*	*sgd*	0.636363636	0.75	0.5
50	*identity*	*sgd*	0.545454545	0.571428571	0.666666667
50	*identity*	*sgd*	0.454545455	0.666666667	0.5
50	*identity*	*sgd*	0.545454545	0.571428571	0.666666667
50	*identity*	*sgd*	0.818181818	1	0.714285714
50	*identity*	*sgd*	0.727272727	0.727272727	1
50	*identity*	*sgd*	0.636363636	0.833333333	0.625
50	*identity*	*sgd*	0.545454545	0.714285714	0.625
50	*identity*	*sgd*	0.545454545	0.571428571	0.666666667
50	*identity*	*sgd*	0.454545455	0.6	0.428571429
50	*identity*	*sgd*	0.636363636	0.833333333	0.625
50	*identity*	*sgd*	0.818181818	0.818181818	1
50	*identity*	*sgd*	0.363636364	0.6	0.375
50	*identity*	*sgd*	0.545454545	0.666666667	0.571428571
50	*identity*	*sgd*	0.636363636	0.571428571	0.8
50	*identity*	*sgd*	0.545454545	0.833333333	0.555555556
50	*identity*	*sgd*	0.545454545	0.428571429	0.75

50	*identity*	*sgd*	0.818181818	1	0.666666667
50	*identity*	*sgd*	0.545454545	0.714285714	0.625
50	*identity*	*sgd*	0.727272727	0.75	0.857142857
50	*identity*	*sgd*	0.363636364	0.5	0.428571429
50	*identity*	*sgd*	0.818181818	0.833333333	0.833333333
50	*identity*	*sgd*	0.454545455	0.666666667	0.5
50	*identity*	*sgd*	0.454545455	0.8	0.444444444
50	*identity*	*sgd*	0.272727273	1	0.111111111
50	*identity*	*sgd*	0.727272727	0.714285714	0.833333333
50	*identity*	*sgd*	0.454545455	0.666666667	0.5
50	*identity*	*sgd*	0.818181818	0.818181818	1
50	*identity*	*sgd*	0.636363636	0.75	0.5
50	*identity*	*sgd*	0.818181818	0.8	1
50	*identity*	*sgd*	0.363636364	0.666666667	0.25
50	*identity*	*sgd*	0.818181818	0.75	0.75
50	*identity*	*sgd*	0.727272727	0.727272727	1
50	*identity*	*sgd*	0.454545455	0.375	0.75
50	*identity*	*sgd*	0.636363636	0.571428571	0.8
50	*identity*	*sgd*	0.909090909	0.8	1
50	*identity*	*sgd*	0.636363636	0.833333333	0.625
50	*identity*	*sgd*	0.545454545	0.571428571	0.666666667
50	*identity*	*sgd*	0.454545455	0.625	0.625
50	*logistic*	*adam*	0.727272727	0.777777778	0.875
50	*logistic*	*adam*	0.818181818	0.8	1
50	*logistic*	*adam*	0.636363636	0.5	0.75
50	*logistic*	*adam*	0.454545455	0.4	1
50	*logistic*	*adam*	0.818181818	0.777777778	1
50	*logistic*	*adam*	0.818181818	0.818181818	1
50	*logistic*	*adam*	0.818181818	0.888888889	0.888888889
50	*logistic*	*adam*	0.545454545	0.545454545	1
50	*logistic*	*adam*	0.636363636	0.7	0.875
50	*logistic*	*adam*	0.727272727	0.8	0.888888889
50	*logistic*	*adam*	0.636363636	0.6	1
50	*logistic*	*adam*	0.727272727	1	0.666666667
50	*logistic*	*adam*	0.818181818	0.888888889	0.888888889
50	*logistic*	*adam*	0.727272727	0.714285714	0.833333333
50	*logistic*	*adam*	0.818181818	0.666666667	1
50	*logistic*	*adam*	0.727272727	0.714285714	0.833333333
50	*logistic*	*adam*	0.636363636	0.555555556	1
50	*logistic*	*adam*	0.727272727	0.727272727	1
50	*logistic*	*adam*	0.727272727	0.875	0.777777778
50	*logistic*	*adam*	0.636363636	0.666666667	0.857142857
50	*logistic*	*adam*	0.727272727	0.7	1

50	*logistic*	*adam*	0.909090909	0.888888889	1
50	*logistic*	*adam*	0.727272727	0.666666667	0.8
50	*logistic*	*adam*	0.727272727	0.75	0.857142857
50	*logistic*	*adam*	0.818181818	0.8	1
50	*logistic*	*adam*	0.545454545	0.444444444	1
50	*logistic*	*adam*	1	1	1
50	*logistic*	*adam*	0.818181818	0.8	1
50	*logistic*	*adam*	0.545454545	0.666666667	0.75
50	*logistic*	*adam*	0.545454545	0.571428571	0.666666667
50	*logistic*	*adam*	0.818181818	0.8	1
50	*logistic*	*adam*	0.636363636	0.7	0.875
50	*logistic*	*adam*	0.636363636	0.625	0.833333333
50	*logistic*	*adam*	0.636363636	0.6	1
50	*logistic*	*adam*	0.818181818	1	0.777777778
50	*logistic*	*adam*	0.727272727	0.666666667	1
50	*logistic*	*adam*	0.818181818	0.777777778	1
50	*logistic*	*adam*	0.636363636	0.666666667	0.857142857
50	*logistic*	*adam*	0.727272727	0.7	1
50	*logistic*	*adam*	0.818181818	0.75	1
50	*logistic*	*adam*	0.818181818	0.875	0.875
50	*logistic*	*adam*	0.636363636	0.7	0.875
50	*logistic*	*adam*	0.727272727	0.777777778	0.875
50	*logistic*	*adam*	0.909090909	0.857142857	1
50	*logistic*	*adam*	0.727272727	0.7	1
50	*logistic*	*adam*	0.727272727	0.777777778	0.875
50	*logistic*	*adam*	0.818181818	0.8	1
50	*logistic*	*adam*	0.818181818	0.833333333	0.833333333
50	*logistic*	*adam*	0.727272727	0.666666667	0.8
50	*logistic*	*adam*	0.727272727	0.666666667	1
50	*logistic*	*lbfgs*	0.909090909	0.857142857	1
50	*logistic*	*lbfgs*	0.545454545	0.5	0.6
50	*logistic*	*lbfgs*	0.454545455	0.5	0.833333333
50	*logistic*	*lbfgs*	0.636363636	0.777777778	0.777777778
50	*logistic*	*lbfgs*	0.545454545	0.625	0.714285714
50	*logistic*	*lbfgs*	0.454545455	0.5	0.333333333
50	*logistic*	*lbfgs*	0.545454545	1	0.444444444
50	*logistic*	*lbfgs*	0.909090909	0.857142857	1
50	*logistic*	*lbfgs*	0.727272727	0.7	1
50	*logistic*	*lbfgs*	0.727272727	0.571428571	1
50	*logistic*	*lbfgs*	0.636363636	0.75	0.75
50	*logistic*	*lbfgs*	0.545454545	0.444444444	1
50	*logistic*	*lbfgs*	0.545454545	0.6	0.5
50	*logistic*	*lbfgs*	0.363636364	0	0

50	*logistic*	*lbfgs*	0.545454545	0.75	0.666666667
50	*logistic*	*lbfgs*	0.727272727	0.875	0.777777778
50	*logistic*	*lbfgs*	0.545454545	0.666666667	0.75
50	*logistic*	*lbfgs*	0.545454545	0.6	0.5
50	*logistic*	*lbfgs*	0.727272727	0.7	1
50	*logistic*	*lbfgs*	0.818181818	0.777777778	1
50	*logistic*	*lbfgs*	0.545454545	0.75	0.666666667
50	*logistic*	*lbfgs*	0.636363636	1	0.5
50	*logistic*	*lbfgs*	0.545454545	0.555555556	0.833333333
50	*logistic*	*lbfgs*	0.545454545	0.555555556	0.833333333
50	*logistic*	*lbfgs*	0.727272727	1	0.666666667
50	*logistic*	*lbfgs*	0.727272727	0.8	0.888888889
50	*logistic*	*lbfgs*	1	1	1
50	*logistic*	*lbfgs*	0.636363636	0.571428571	0.8
50	*logistic*	*lbfgs*	0.818181818	0.75	1
50	*logistic*	*lbfgs*	0.727272727	0.777777778	0.875
50	*logistic*	*lbfgs*	0.545454545	0.8	0.5
50	*logistic*	*lbfgs*	0.909090909	0.875	1
50	*logistic*	*lbfgs*	0.545454545	0.666666667	0.75
50	*logistic*	*lbfgs*	0.545454545	0.666666667	0.75
50	*logistic*	*lbfgs*	0.727272727	0.777777778	0.875
50	*logistic*	*lbfgs*	0.545454545	0.571428571	0.666666667
50	*logistic*	*lbfgs*	0.727272727	0.571428571	1
50	*logistic*	*lbfgs*	0.636363636	0.666666667	0.666666667
50	*logistic*	*lbfgs*	0.727272727	0.857142857	0.75
50	*logistic*	*lbfgs*	0.545454545	0.625	0.714285714
50	*logistic*	*lbfgs*	0.636363636	0.7	0.875
50	*logistic*	*lbfgs*	0.363636364	0.5	0.428571429
50	*logistic*	*lbfgs*	0.636363636	0.625	0.833333333
50	*logistic*	*lbfgs*	0.454545455	0.625	0.625
50	*logistic*	*lbfgs*	0.727272727	0.777777778	0.875
50	*logistic*	*lbfgs*	0.727272727	0.8	0.666666667
50	*logistic*	*lbfgs*	0.636363636	0.666666667	0.857142857
50	*logistic*	*lbfgs*	0.636363636	0.5	1
50	*logistic*	*lbfgs*	0.363636364	0.6	0.375
50	*logistic*	*lbfgs*	0.818181818	0.8	1
50	*logistic*	*sgd*	0.727272727	0.727272727	1
50	*logistic*	*sgd*	0.545454545	0.545454545	1
50	*logistic*	*sgd*	0.727272727	0.7	1
50	*logistic*	*sgd*	0.636363636	0.7	0.875
50	*logistic*	*sgd*	0.727272727	0.727272727	1
50	*logistic*	*sgd*	0.636363636	0.636363636	1
50	*logistic*	*sgd*	0.545454545	0.545454545	1

50	*logistic*	*sgd*	0.545454545	0.5	1
50	*logistic*	*sgd*	0.454545455	0.5	0.833333333
50	*logistic*	*sgd*	0.545454545	0.545454545	1
50	*logistic*	*sgd*	0.545454545	0.545454545	1
50	*logistic*	*sgd*	0.545454545	0.6	0.857142857
50	*logistic*	*sgd*	0.454545455	0.454545455	1
50	*logistic*	*sgd*	0.727272727	0.8	0.888888889
50	*logistic*	*sgd*	0.454545455	0.4	1
50	*logistic*	*sgd*	0.545454545	0.545454545	1
50	*logistic*	*sgd*	0.727272727	0.727272727	1
50	*logistic*	*sgd*	0.727272727	0.727272727	1
50	*logistic*	*sgd*	0.545454545	0.545454545	1
50	*logistic*	*sgd*	0.636363636	0.636363636	1
50	*logistic*	*sgd*	0.636363636	0.7	0.875
50	*logistic*	*sgd*	0.909090909	1	0.888888889
50	*logistic*	*sgd*	0.727272727	0.727272727	1
50	*logistic*	*sgd*	0.727272727	0.875	0.777777778
50	*logistic*	*sgd*	0.727272727	0.727272727	1
50	*logistic*	*sgd*	0.818181818	0.818181818	1
50	*logistic*	*sgd*	0.636363636	0.625	0.833333333
50	*logistic*	*sgd*	0.545454545	0.545454545	1
50	*logistic*	*sgd*	0.545454545	0.6	0.857142857
50	*logistic*	*sgd*	0.727272727	0.7	1
50	*logistic*	*sgd*	0.363636364	0.363636364	1
50	*logistic*	*sgd*	0.636363636	0.7	0.875
50	*logistic*	*sgd*	0.363636364	0.363636364	1
50	*logistic*	*sgd*	0.454545455	0.454545455	1
50	*logistic*	*sgd*	0.636363636	0.7	0.875
50	*logistic*	*sgd*	0.727272727	0.727272727	1
50	*logistic*	*sgd*	0.636363636	0.6	1
50	*logistic*	*sgd*	0.727272727	0.777777778	0.875
50	*logistic*	*sgd*	0.363636364	0.363636364	1
50	*logistic*	*sgd*	0.636363636	0.7	0.875
50	*logistic*	*sgd*	0.818181818	0.818181818	1
50	*logistic*	*sgd*	0.818181818	0.818181818	1
50	*logistic*	*sgd*	0.636363636	0.636363636	1
50	*logistic*	*sgd*	0.727272727	0.7	1
50	*logistic*	*sgd*	0.545454545	0.545454545	1
50	*logistic*	*sgd*	0.727272727	0.727272727	1
50	*logistic*	*sgd*	0.727272727	0.8	0.888888889
50	*logistic*	*sgd*	0.545454545	0.545454545	1
50	*logistic*	*sgd*	0.545454545	0.545454545	1
50	*logistic*	*sgd*	0.636363636	0.7	0.875

50	*relu*	*adam*	0.454545455	0.454545455	1
50	*relu*	*adam*	0.818181818	0.8	1
50	*relu*	*adam*	1	1	1
50	*relu*	*adam*	0.636363636	0.625	0.833333333
50	*relu*	*adam*	0.727272727	0.75	0.857142857
50	*relu*	*adam*	0.636363636	1	0.555555556
50	*relu*	*adam*	0.636363636	0.75	0.5
50	*relu*	*adam*	0.909090909	1	0.875
50	*relu*	*adam*	0.636363636	0.6	1
50	*relu*	*adam*	0.818181818	0.833333333	0.833333333
50	*relu*	*adam*	0.636363636	0.7	0.875
50	*relu*	*adam*	0.272727273	0	0
50	*relu*	*adam*	0.818181818	0.875	0.875
50	*relu*	*adam*	0.818181818	0.8	0.8
50	*relu*	*adam*	0.818181818	0.833333333	0.833333333
50	*relu*	*adam*	0.818181818	0.875	0.875
50	*relu*	*adam*	0.909090909	0.857142857	1
50	*relu*	*adam*	0.636363636	0.833333333	0.625
50	*relu*	*adam*	0.545454545	0.545454545	1
50	*relu*	*adam*	1	1	1
50	*relu*	*adam*	0.636363636	0.666666667	0.857142857
50	*relu*	*adam*	0.909090909	1	0.875
50	*relu*	*adam*	0.818181818	1	0.75
50	*relu*	*adam*	0.636363636	0.75	0.75
50	*relu*	*adam*	0.636363636	0.8	0.571428571
50	*relu*	*adam*	0.909090909	0.9	1
50	*relu*	*adam*	0.636363636	0.636363636	1
50	*relu*	*adam*	0.727272727	1	0.625
50	*relu*	*adam*	0.818181818	0.875	0.875
50	*relu*	*adam*	0.272727273	1	0.111111111
50	*relu*	*adam*	0.636363636	0.75	0.5
50	*relu*	*adam*	0.727272727	0.571428571	1
50	*relu*	*adam*	0.727272727	0.6	0.75
50	*relu*	*adam*	0.454545455	0.333333333	0.5
50	*relu*	*adam*	0.454545455	0	0
50	*relu*	*adam*	0.545454545	0.75	0.666666667
50	*relu*	*adam*	0.545454545	0.545454545	1
50	*relu*	*adam*	0.545454545	0.545454545	1
50	*relu*	*adam*	0.909090909	1	0.888888889
50	*relu*	*adam*	0.727272727	0.833333333	0.714285714
50	*relu*	*adam*	0.818181818	0.8	1
50	*relu*	*adam*	0.727272727	0.833333333	0.714285714
50	*relu*	*adam*	0.909090909	0.857142857	1

50	*relu*	*adam*	0.727272727	0.857142857	0.75
50	*relu*	*adam*	0.818181818	0.875	0.875
50	*relu*	*adam*	0.909090909	1	0.888888889
50	*relu*	*adam*	0.454545455	0.5	0.833333333
50	*relu*	*adam*	0.909090909	1	0.75
50	*relu*	*adam*	0.909090909	1	0.888888889
50	*relu*	*adam*	0.909090909	0.875	1
50	*relu*	*lbfgs*	0.818181818	0.875	0.875
50	*relu*	*lbfgs*	0.909090909	0.875	1
50	*relu*	*lbfgs*	0.545454545	0.4	0.5
50	*relu*	*lbfgs*	0.727272727	0.8	0.888888889
50	*relu*	*lbfgs*	0.818181818	0.875	0.875
50	*relu*	*lbfgs*	0.636363636	0.857142857	0.666666667
50	*relu*	*lbfgs*	0.727272727	0.727272727	1
50	*relu*	*lbfgs*	0.818181818	0.8	1
50	*relu*	*lbfgs*	0.909090909	0.857142857	1
50	*relu*	*lbfgs*	0.363636364	0.363636364	1
50	*relu*	*lbfgs*	0.818181818	0.875	0.875
50	*relu*	*lbfgs*	0.636363636	0.666666667	0.666666667
50	*relu*	*lbfgs*	0.909090909	0.833333333	1
50	*relu*	*lbfgs*	0.545454545	0.571428571	0.666666667
50	*relu*	*lbfgs*	0.909090909	0.8	1
50	*relu*	*lbfgs*	0.636363636	0.75	0.75
50	*relu*	*lbfgs*	0.454545455	0.454545455	1
50	*relu*	*lbfgs*	0.727272727	0.7	1
50	*relu*	*lbfgs*	1	1	1
50	*relu*	*lbfgs*	1	1	1
50	*relu*	*lbfgs*	0.727272727	0.571428571	1
50	*relu*	*lbfgs*	0.818181818	0.875	0.875
50	*relu*	*lbfgs*	1	1	1
50	*relu*	*lbfgs*	0.818181818	0.75	1
50	*relu*	*lbfgs*	0.545454545	0.625	0.714285714
50	*relu*	*lbfgs*	0.727272727	0.777777778	0.875
50	*relu*	*lbfgs*	0.727272727	0.777777778	0.875
50	*relu*	*lbfgs*	0.545454545	0.8	0.5
50	*relu*	*lbfgs*	0.818181818	0.857142857	0.857142857
50	*relu*	*lbfgs*	1	1	1
50	*relu*	*lbfgs*	0.636363636	0.8	0.571428571
50	*relu*	*lbfgs*	0.909090909	0.857142857	1
50	*relu*	*lbfgs*	0.636363636	0.571428571	0.8
50	*relu*	*lbfgs*	0.727272727	0.75	0.857142857
50	*relu*	*lbfgs*	0.909090909	0.857142857	1
50	*relu*	*lbfgs*	0.909090909	1	0.888888889

50	*relu*	*lbfgs*	0.909090909	0.857142857	1
50	*relu*	*lbfgs*	0.727272727	0.8	0.888888889
50	*relu*	*lbfgs*	0.727272727	0.8	0.666666667
50	*relu*	*lbfgs*	0.636363636	0.7	0.875
50	*relu*	*lbfgs*	0.727272727	0.857142857	0.75
50	*relu*	*lbfgs*	0.818181818	0.75	1
50	*relu*	*lbfgs*	0.636363636	0.636363636	1
50	*relu*	*lbfgs*	0.909090909	0.857142857	1
50	*relu*	*lbfgs*	0.818181818	0.818181818	1
50	*relu*	*lbfgs*	0.727272727	0.857142857	0.75
50	*relu*	*lbfgs*	1	1	1
50	*relu*	*lbfgs*	0.818181818	0.818181818	1
50	*relu*	*lbfgs*	0.909090909	1	0.888888889
50	*relu*	*lbfgs*	0.545454545	0.545454545	1
50	*relu*	*sgd*	0.636363636	1	0.555555556
50	*relu*	*sgd*	0.363636364	0.4	0.333333333
50	*relu*	*sgd*	0.727272727	0.727272727	1
50	*relu*	*sgd*	0.727272727	0.777777778	0.875
50	*relu*	*sgd*	0.727272727	0.8	0.888888889
50	*relu*	*sgd*	0.545454545	0.8	0.5
50	*relu*	*sgd*	0.636363636	0.6	1
50	*relu*	*sgd*	0.545454545	0.545454545	1
50	*relu*	*sgd*	0.818181818	0.818181818	1
50	*relu*	*sgd*	0.818181818	1	0.75
50	*relu*	*sgd*	0.636363636	0.6	1
50	*relu*	*sgd*	0.818181818	0.8	1
50	*relu*	*sgd*	0.636363636	0.6	1
50	*relu*	*sgd*	0.545454545	0.545454545	1
50	*relu*	*sgd*	0.545454545	0.545454545	1
50	*relu*	*sgd*	0.727272727	0.727272727	1
50	*relu*	*sgd*	0.363636364	0.5	0.571428571
50	*relu*	*sgd*	0.727272727	0.75	0.857142857
50	*relu*	*sgd*	0.545454545	0.666666667	0.571428571
50	*relu*	*sgd*	0.636363636	0.7	0.875
50	*relu*	*sgd*	0.272727273	1	0.111111111
50	*relu*	*sgd*	0.545454545	0.6	0.857142857
50	*relu*	*sgd*	0.636363636	0.7	0.875
50	*relu*	*sgd*	0.545454545	0.666666667	0.75
50	*relu*	*sgd*	0.545454545	0.555555556	0.833333333
50	*relu*	*sgd*	0.363636364	0.4	0.8
50	*relu*	*sgd*	0.727272727	0.727272727	1
50	*relu*	*sgd*	0.727272727	0.727272727	1
50	*relu*	*sgd*	0.363636364	0.363636364	1

50	*relu*	*sgd*	0.545454545	0.555555556	0.833333333
50	*relu*	*sgd*	0.545454545	0.571428571	0.666666667
50	*relu*	*sgd*	0.727272727	1	0.4
50	*relu*	*sgd*	0.636363636	0.636363636	1
50	*relu*	*sgd*	0.545454545	0.75	0.666666667
50	*relu*	*sgd*	0.727272727	0.777777778	0.875
50	*relu*	*sgd*	0.545454545	0.555555556	0.833333333
50	*relu*	*sgd*	0.545454545	0.545454545	1
50	*relu*	*sgd*	0.363636364	0.363636364	1
50	*relu*	*sgd*	0.636363636	0.636363636	1
50	*relu*	*sgd*	0.181818182	0	0
50	*relu*	*sgd*	0.454545455	0.4	1
50	*relu*	*sgd*	0.181818182	0	0
50	*relu*	*sgd*	0.181818182	0.333333333	0.125
50	*relu*	*sgd*	0.818181818	0.8	1
50	*relu*	*sgd*	0.545454545	0.5	0.2
50	*relu*	*sgd*	0.545454545	0.6	0.857142857
50	*relu*	*sgd*	0.545454545	0.444444444	1
50	*relu*	*sgd*	0.727272727	0.8	0.888888889
50	*relu*	*sgd*	0.363636364	0.666666667	0.25
50	*relu*	*sgd*	0.545454545	0.6	0.5
50	*tanh*	*adam*	0.636363636	0.7	0.875
50	*tanh*	*adam*	0.454545455	0.555555556	0.714285714
50	*tanh*	*adam*	0.636363636	0.75	0.75
50	*tanh*	*adam*	0.727272727	0.714285714	0.833333333
50	*tanh*	*adam*	0.818181818	0.777777778	1
50	*tanh*	*adam*	0.545454545	0.555555556	0.833333333
50	*tanh*	*adam*	0.727272727	0.666666667	1
50	*tanh*	*adam*	0.818181818	1	0.777777778
50	*tanh*	*adam*	0.818181818	0.818181818	1
50	*tanh*	*adam*	0.545454545	0.6	0.857142857
50	*tanh*	*adam*	0.727272727	0.875	0.777777778
50	*tanh*	*adam*	0.454545455	0.5	0.833333333
50	*tanh*	*adam*	0.636363636	0.5	0.75
50	*tanh*	*adam*	0.545454545	0.714285714	0.625
50	*tanh*	*adam*	0.636363636	0.7	0.875
50	*tanh*	*adam*	0.454545455	0.714285714	0.555555556
50	*tanh*	*adam*	0.909090909	1	0.875
50	*tanh*	*adam*	0.818181818	0.777777778	1
50	*tanh*	*adam*	0.636363636	0.777777778	0.777777778
50	*tanh*	*adam*	0.545454545	0.6	0.857142857
50	*tanh*	*adam*	0.818181818	0.875	0.875
50	*tanh*	*adam*	0.636363636	0.6	1

50	*tanh*	*adam*	0.727272727	0.8	0.888888889
50	*tanh*	*adam*	0.272727273	0.3	0.75
50	*tanh*	*adam*	0.727272727	0.666666667	1
50	*tanh*	*adam*	0.454545455	0.4	1
50	*tanh*	*adam*	0.363636364	0.363636364	1
50	*tanh*	*adam*	0.636363636	0.6	1
50	*tanh*	*adam*	0.818181818	0.8	1
50	*tanh*	*adam*	0.727272727	0.777777778	0.875
50	*tanh*	*adam*	0.636363636	0.571428571	0.8
50	*tanh*	*adam*	0.545454545	0.571428571	0.666666667
50	*tanh*	*adam*	0.545454545	0.666666667	0.75
50	*tanh*	*adam*	0.818181818	0.875	0.875
50	*tanh*	*adam*	0.363636364	0.5	0.571428571
50	*tanh*	*adam*	0.818181818	0.8	1
50	*tanh*	*adam*	0.636363636	0.625	0.833333333
50	*tanh*	*adam*	0.818181818	0.777777778	1
50	*tanh*	*adam*	0.636363636	0.6	1
50	*tanh*	*adam*	0.545454545	1	0.444444444
50	*tanh*	*adam*	0.545454545	0.625	0.714285714
50	*tanh*	*adam*	0.545454545	0.5	0.8
50	*tanh*	*adam*	0.727272727	0.625	1
50	*tanh*	*adam*	0.818181818	0.875	0.875
50	*tanh*	*adam*	0.636363636	0.75	0.75
50	*tanh*	*adam*	0.727272727	0.727272727	1
50	*tanh*	*adam*	0.454545455	0.5	0.833333333
50	*tanh*	*adam*	0.727272727	0.714285714	0.833333333
50	*tanh*	*adam*	0.545454545	0.6	0.5
50	*tanh*	*adam*	0.727272727	0.777777778	0.875
50	*tanh*	*lbfgs*	0.454545455	0.375	0.75
50	*tanh*	*lbfgs*	0.636363636	0.777777778	0.777777778
50	*tanh*	*lbfgs*	0.636363636	0.75	0.75
50	*tanh*	*lbfgs*	0.454545455	0.571428571	0.571428571
50	*tanh*	*lbfgs*	0.545454545	0.714285714	0.625
50	*tanh*	*lbfgs*	0.545454545	0.6	0.5
50	*tanh*	*lbfgs*	0.545454545	0.444444444	1
50	*tanh*	*lbfgs*	0.454545455	0.666666667	0.5
50	*tanh*	*lbfgs*	0.727272727	0.875	0.777777778
50	*tanh*	*lbfgs*	0.727272727	0.777777778	0.875
50	*tanh*	*lbfgs*	0.545454545	0.571428571	0.666666667
50	*tanh*	*lbfgs*	0.818181818	0.666666667	1
50	*tanh*	*lbfgs*	0.545454545	0.8	0.5
50	*tanh*	*lbfgs*	0.636363636	0.75	0.75
50	*tanh*	*lbfgs*	0.636363636	0.625	0.833333333

50	*tanh*	*lbfgs*	0.545454545	0.5	0.6
50	*tanh*	*lbfgs*	0.909090909	1	0.875
50	*tanh*	*lbfgs*	0.545454545	0.444444444	1
50	*tanh*	*lbfgs*	0.818181818	0.875	0.875
50	*tanh*	*lbfgs*	0.545454545	0.5	0.2
50	*tanh*	*lbfgs*	0.454545455	0.571428571	0.571428571
50	*tanh*	*lbfgs*	0.454545455	0.5	0.5
50	*tanh*	*lbfgs*	0.636363636	0.75	0.75
50	*tanh*	*lbfgs*	0.636363636	0.833333333	0.625
50	*tanh*	*lbfgs*	0.545454545	0.714285714	0.625
50	*tanh*	*lbfgs*	0.727272727	0.75	0.857142857
50	*tanh*	*lbfgs*	0.818181818	0.857142857	0.857142857
50	*tanh*	*lbfgs*	0.727272727	1	0.5
50	*tanh*	*lbfgs*	0.727272727	0.833333333	0.714285714
50	*tanh*	*lbfgs*	0.727272727	0.625	1
50	*tanh*	*lbfgs*	0.818181818	0.857142857	0.857142857
50	*tanh*	*lbfgs*	0.727272727	0.666666667	1
50	*tanh*	*lbfgs*	0.727272727	1	0.666666667
50	*tanh*	*lbfgs*	0.545454545	0.666666667	0.75
50	*tanh*	*lbfgs*	0.545454545	0.6	0.857142857
50	*tanh*	*lbfgs*	0.636363636	0.6	1
50	*tanh*	*lbfgs*	0.545454545	0.666666667	0.75
50	*tanh*	*lbfgs*	0.818181818	0.8	1
50	*tanh*	*lbfgs*	0.636363636	0.625	0.833333333
50	*tanh*	*lbfgs*	0.454545455	0.571428571	0.571428571
50	*tanh*	*lbfgs*	0.454545455	0.5	0.5
50	*tanh*	*lbfgs*	0.727272727	1	0.666666667
50	*tanh*	*lbfgs*	0.454545455	0.5	0.5
50	*tanh*	*lbfgs*	0.818181818	0.833333333	0.833333333
50	*tanh*	*lbfgs*	0.636363636	0.857142857	0.666666667
50	*tanh*	*lbfgs*	0.727272727	0.875	0.777777778
50	*tanh*	*lbfgs*	0.727272727	0.8	0.666666667
50	*tanh*	*lbfgs*	0.454545455	0.5	0.5
50	*tanh*	*lbfgs*	0.727272727	0.8	0.888888889
50	*tanh*	*lbfgs*	0.636363636	0.833333333	0.625
50	*tanh*	*sgd*	0.818181818	0.777777778	1
50	*tanh*	*sgd*	0.545454545	0.428571429	0.75
50	*tanh*	*sgd*	0.636363636	0.571428571	0.8
50	*tanh*	*sgd*	0.545454545	0.6	0.5
50	*tanh*	*sgd*	0.545454545	0.666666667	0.75
50	*tanh*	*sgd*	0.545454545	0.75	0.666666667
50	*tanh*	*sgd*	0.727272727	0.7	1
50	*tanh*	*sgd*	0.454545455	0.571428571	0.571428571

50	*tanh*	*sgd*	0.545454545	0.444444444	1
50	*tanh*	*sgd*	0.818181818	0.888888889	0.888888889
50	*tanh*	*sgd*	0.454545455	0.625	0.625
50	*tanh*	*sgd*	0.727272727	0.727272727	1
50	*tanh*	*sgd*	0.545454545	0.555555556	0.833333333
50	*tanh*	*sgd*	0.727272727	0.875	0.777777778
50	*tanh*	*sgd*	0.727272727	0.75	0.857142857
50	*tanh*	*sgd*	0.636363636	0.7	0.875
50	*tanh*	*sgd*	0.545454545	0.444444444	1
50	*tanh*	*sgd*	0.545454545	0.571428571	0.666666667
50	*tanh*	*sgd*	0.727272727	0.727272727	1
50	*tanh*	*sgd*	0.545454545	0.428571429	0.75
50	*tanh*	*sgd*	0.545454545	0.666666667	0.75
50	*tanh*	*sgd*	0.454545455	0.454545455	1
50	*tanh*	*sgd*	0.545454545	0.571428571	0.666666667
50	*tanh*	*sgd*	0.636363636	0.625	0.833333333
50	*tanh*	*sgd*	0.636363636	0.7	0.875
50	*tanh*	*sgd*	0.545454545	0.666666667	0.571428571
50	*tanh*	*sgd*	0.727272727	0.8	0.888888889
50	*tanh*	*sgd*	0.454545455	0.454545455	1
50	*tanh*	*sgd*	1	1	1
50	*tanh*	*sgd*	0.636363636	0.6	1
50	*tanh*	*sgd*	0.636363636	0.75	0.75
50	*tanh*	*sgd*	0.727272727	0.75	0.857142857
50	*tanh*	*sgd*	0.818181818	0.75	1
50	*tanh*	*sgd*	0.454545455	0.571428571	0.571428571
50	*tanh*	*sgd*	0.636363636	0.833333333	0.625
50	*tanh*	*sgd*	0.727272727	0.7	1
50	*tanh*	*sgd*	0.818181818	0.888888889	0.888888889
50	*tanh*	*sgd*	0.909090909	1	0.875
50	*tanh*	*sgd*	0.636363636	0.666666667	0.666666667
50	*tanh*	*sgd*	0.545454545	0.571428571	0.666666667
50	*tanh*	*sgd*	0.636363636	0.666666667	0.666666667
50	*tanh*	*sgd*	0.727272727	0.666666667	1
50	*tanh*	*sgd*	0.727272727	0.727272727	1
50	*tanh*	*sgd*	0.545454545	0.555555556	0.833333333
50	*tanh*	*sgd*	0.818181818	0.8	1
50	*tanh*	*sgd*	0.636363636	0.6	1
50	*tanh*	*sgd*	0.818181818	0.818181818	1
50	*tanh*	*sgd*	0.545454545	0.666666667	0.75
50	*tanh*	*sgd*	0.818181818	0.875	0.875
50	*tanh*	*sgd*	0.818181818	0.888888889	0.888888889
100	*identity*	*adam*	0.909090909	1	0.888888889

100	*identity*	*adam*	0.181818182	0.4	0.25
100	*identity*	*adam*	1	1	1
100	*identity*	*adam*	0.818181818	0.833333333	0.833333333
100	*identity*	*adam*	0.636363636	1	0.5
100	*identity*	*adam*	0.818181818	0.8	1
100	*identity*	*adam*	0.909090909	1	0.857142857
100	*identity*	*adam*	0.909090909	0.888888889	1
100	*identity*	*adam*	0.545454545	0.666666667	0.75
100	*identity*	*adam*	0.909090909	0.888888889	1
100	*identity*	*adam*	0.909090909	0.857142857	1
100	*identity*	*adam*	0.818181818	0.666666667	1
100	*identity*	*adam*	0.545454545	0.6	0.5
100	*identity*	*adam*	0.727272727	0.777777778	0.875
100	*identity*	*adam*	1	1	1
100	*identity*	*adam*	0.636363636	0.75	0.75
100	*identity*	*adam*	0.636363636	1	0.428571429
100	*identity*	*adam*	0.909090909	0.857142857	1
100	*identity*	*adam*	0.727272727	0.777777778	0.875
100	*identity*	*adam*	1	1	1
100	*identity*	*adam*	1	1	1
100	*identity*	*adam*	0.909090909	1	0.857142857
100	*identity*	*adam*	0.454545455	0.375	0.75
100	*identity*	*adam*	1	1	1
100	*identity*	*adam*	0.909090909	1	0.875
100	*identity*	*adam*	0.818181818	1	0.666666667
100	*identity*	*adam*	0.818181818	0.714285714	1
100	*identity*	*adam*	0.545454545	0.6	0.5
100	*identity*	*adam*	1	1	1
100	*identity*	*adam*	0.545454545	0.666666667	0.571428571
100	*identity*	*adam*	0.909090909	0.857142857	1
100	*identity*	*adam*	0.454545455	1	0.333333333
100	*identity*	*adam*	0.272727273	0.375	0.5
100	*identity*	*adam*	0.909090909	0.875	1
100	*identity*	*adam*	1	1	1
100	*identity*	*adam*	1	1	1
100	*identity*	*adam*	0.454545455	0.333333333	0.5
100	*identity*	*adam*	1	1	1
100	*identity*	*adam*	0.818181818	1	0.6
100	*identity*	*adam*	0.727272727	0.8	0.888888889
100	*identity*	*adam*	1	1	1
100	*identity*	*adam*	0.727272727	0.777777778	0.875
100	*identity*	*adam*	0.909090909	1	0.833333333
100	*identity*	*adam*	0.909090909	0.875	1

100	*identity*	*adam*	0.545454545	0.625	0.714285714
100	*identity*	*adam*	0.909090909	1	0.888888889
100	*identity*	*adam*	1	1	1
100	*identity*	*adam*	1	1	1
100	*identity*	*adam*	0.636363636	1	0.333333333
100	*identity*	*adam*	0.909090909	1	0.888888889
100	*identity*	*lbfgs*	1	1	1
100	*identity*	*lbfgs*	0.909090909	0.875	1
100	*identity*	*lbfgs*	0.909090909	1	0.875
100	*identity*	*lbfgs*	1	1	1
100	*identity*	*lbfgs*	0.818181818	0.75	1
100	*identity*	*lbfgs*	0.727272727	0.727272727	1
100	*identity*	*lbfgs*	0.818181818	0.818181818	1
100	*identity*	*lbfgs*	1	1	1
100	*identity*	*lbfgs*	0.272727273	0.333333333	0.6
100	*identity*	*lbfgs*	0.727272727	0.727272727	1
100	*identity*	*lbfgs*	0.909090909	0.888888889	1
100	*identity*	*lbfgs*	0.909090909	1	0.833333333
100	*identity*	*lbfgs*	0.636363636	0.636363636	1
100	*identity*	*lbfgs*	0.363636364	0.363636364	1
100	*identity*	*lbfgs*	0.909090909	1	0.857142857
100	*identity*	*lbfgs*	1	1	1
100	*identity*	*lbfgs*	0.727272727	0.727272727	1
100	*identity*	*lbfgs*	0.545454545	0.545454545	1
100	*identity*	*lbfgs*	0.909090909	1	0.875
100	*identity*	*lbfgs*	0.909090909	0.875	1
100	*identity*	*lbfgs*	0.909090909	0.857142857	1
100	*identity*	*lbfgs*	0.909090909	0.8	1
100	*identity*	*lbfgs*	0.909090909	0.888888889	1
100	*identity*	*lbfgs*	0.818181818	1	0.777777778
100	*identity*	*lbfgs*	0.545454545	0.545454545	1
100	*identity*	*lbfgs*	0.545454545	0.545454545	1
100	*identity*	*lbfgs*	0.545454545	0.545454545	1
100	*identity*	*lbfgs*	0.727272727	0.666666667	1
100	*identity*	*lbfgs*	0.545454545	0.545454545	1
100	*identity*	*lbfgs*	0.909090909	1	0.875
100	*identity*	*lbfgs*	0.909090909	1	0.888888889
100	*identity*	*lbfgs*	0.818181818	0.875	0.875
100	*identity*	*lbfgs*	0.909090909	0.8	1
100	*identity*	*lbfgs*	0.909090909	1	0.857142857
100	*identity*	*lbfgs*	0.454545455	0.5	0.833333333
100	*identity*	*lbfgs*	0.818181818	0.875	0.875
100	*identity*	*lbfgs*	0.909090909	1	0.833333333

100	*identity*	*lbfgs*	0.363636364	0.363636364	1
100	*identity*	*lbfgs*	0.636363636	0.7	0.875
100	*identity*	*lbfgs*	0.636363636	0.777777778	0.777777778
100	*identity*	*lbfgs*	0.909090909	0.875	1
100	*identity*	*lbfgs*	0.727272727	0.727272727	1
100	*identity*	*lbfgs*	0.909090909	1	0.875
100	*identity*	*lbfgs*	1	1	1
100	*identity*	*lbfgs*	0.454545455	0.454545455	1
100	*identity*	*lbfgs*	0.909090909	1	0.888888889
100	*identity*	*lbfgs*	1	1	1
100	*identity*	*lbfgs*	1	1	1
100	*identity*	*lbfgs*	1	1	1
100	*identity*	*lbfgs*	0.909090909	1	0.857142857
100	*identity*	*sgd*	0.727272727	0.75	0.857142857
100	*identity*	*sgd*	0.727272727	0.7	1
100	*identity*	*sgd*	0.545454545	0.545454545	1
100	*identity*	*sgd*	0.636363636	0.833333333	0.625
100	*identity*	*sgd*	0.818181818	0.8	1
100	*identity*	*sgd*	0.454545455	0.625	0.625
100	*identity*	*sgd*	0.363636364	0.444444444	0.666666667
100	*identity*	*sgd*	0.727272727	0.7	1
100	*identity*	*sgd*	0.727272727	0.857142857	0.75
100	*identity*	*sgd*	0.636363636	0.75	0.75
100	*identity*	*sgd*	0.727272727	0.714285714	0.833333333
100	*identity*	*sgd*	0.545454545	0.5	0.2
100	*identity*	*sgd*	0.818181818	0.833333333	0.833333333
100	*identity*	*sgd*	1	1	1
100	*identity*	*sgd*	0.545454545	0.4	0.5
100	*identity*	*sgd*	0.818181818	0.75	1
100	*identity*	*sgd*	0.545454545	0.714285714	0.625
100	*identity*	*sgd*	0.727272727	0.777777778	0.875
100	*identity*	*sgd*	0.545454545	0.545454545	1
100	*identity*	*sgd*	0.545454545	0.666666667	0.75
100	*identity*	*sgd*	0.727272727	0.727272727	1
100	*identity*	*sgd*	0.545454545	0.571428571	0.666666667
100	*identity*	*sgd*	0.545454545	0.545454545	1
100	*identity*	*sgd*	0.727272727	1	0.666666667
100	*identity*	*sgd*	0.454545455	0.5	0.833333333
100	*identity*	*sgd*	0.636363636	0.8	0.571428571
100	*identity*	*sgd*	0.545454545	0.833333333	0.555555556
100	*identity*	*sgd*	0.545454545	1	0.166666667
100	*identity*	*sgd*	0.727272727	0.6	0.75
100	*identity*	*sgd*	0.545454545	0.6	0.5

100	*identity*	*sgd*	0.272727273	0.2	0.2
100	*identity*	*sgd*	0.636363636	0.75	0.75
100	*identity*	*sgd*	0.272727273	1	0.111111111
100	*identity*	*sgd*	0.818181818	0.777777778	1
100	*identity*	*sgd*	0.545454545	0.666666667	0.571428571
100	*identity*	*sgd*	0.818181818	1	0.75
100	*identity*	*sgd*	0.727272727	0.666666667	0.8
100	*identity*	*sgd*	0.454545455	0.571428571	0.571428571
100	*identity*	*sgd*	0.636363636	0.75	0.75
100	*identity*	*sgd*	0.727272727	0.777777778	0.875
100	*identity*	*sgd*	0.272727273	0.166666667	0.25
100	*identity*	*sgd*	0.636363636	0.5	0.75
100	*identity*	*sgd*	0.454545455	0.555555556	0.714285714
100	*identity*	*sgd*	0.636363636	0.777777778	0.777777778
100	*identity*	*sgd*	0.363636364	0.75	0.333333333
100	*identity*	*sgd*	0.454545455	0.5	0.833333333
100	*identity*	*sgd*	0.363636364	0.6	0.375
100	*identity*	*sgd*	0.363636364	1	0.222222222
100	*identity*	*sgd*	0.545454545	0.6	0.5
100	*identity*	*sgd*	0.636363636	0.857142857	0.666666667
100	*logistic*	*adam*	0.818181818	0.777777778	1
100	*logistic*	*adam*	0.818181818	0.75	1
100	*logistic*	*adam*	0.909090909	0.888888889	1
100	*logistic*	*adam*	0.727272727	0.777777778	0.875
100	*logistic*	*adam*	0.545454545	0.444444444	1
100	*logistic*	*adam*	0.818181818	0.8	1
100	*logistic*	*adam*	0.727272727	0.75	0.857142857
100	*logistic*	*adam*	0.909090909	0.888888889	1
100	*logistic*	*adam*	0.909090909	0.888888889	1
100	*logistic*	*adam*	0.636363636	0.7	0.875
100	*logistic*	*adam*	0.454545455	0.6	0.428571429
100	*logistic*	*adam*	0.636363636	0.6	1
100	*logistic*	*adam*	0.636363636	0.7	0.875
100	*logistic*	*adam*	0.909090909	0.875	1
100	*logistic*	*adam*	0.727272727	0.571428571	1
100	*logistic*	*adam*	0.727272727	0.666666667	1
100	*logistic*	*adam*	0.727272727	0.714285714	0.833333333
100	*logistic*	*adam*	0.727272727	0.727272727	1
100	*logistic*	*adam*	0.909090909	0.888888889	1
100	*logistic*	*adam*	0.636363636	0.571428571	0.8
100	*logistic*	*adam*	0.909090909	0.857142857	1
100	*logistic*	*adam*	1	1	1
100	*logistic*	*adam*	0.818181818	0.818181818	1

100	*logistic*	*adam*	0.818181818	0.8	1
100	*logistic*	*adam*	0.636363636	0.75	0.75
100	*logistic*	*adam*	0.727272727	0.666666667	1
100	*logistic*	*adam*	0.818181818	0.875	0.875
100	*logistic*	*adam*	0.727272727	0.875	0.777777778
100	*logistic*	*adam*	0.727272727	1	0.666666667
100	*logistic*	*adam*	0.727272727	0.714285714	0.833333333
100	*logistic*	*adam*	0.818181818	0.8	1
100	*logistic*	*adam*	0.727272727	0.8	0.888888889
100	*logistic*	*adam*	0.727272727	0.875	0.777777778
100	*logistic*	*adam*	0.636363636	0.571428571	0.8
100	*logistic*	*adam*	0.818181818	0.857142857	0.857142857
100	*logistic*	*adam*	0.727272727	0.7	1
100	*logistic*	*adam*	0.818181818	0.833333333	0.833333333
100	*logistic*	*adam*	0.727272727	0.75	0.857142857
100	*logistic*	*adam*	0.818181818	0.666666667	1
100	*logistic*	*adam*	0.727272727	0.875	0.777777778
100	*logistic*	*adam*	0.909090909	0.857142857	1
100	*logistic*	*adam*	0.909090909	0.857142857	1
100	*logistic*	*adam*	0.545454545	0.444444444	1
100	*logistic*	*adam*	0.818181818	0.8	1
100	*logistic*	*adam*	0.636363636	0.555555556	1
100	*logistic*	*adam*	0.818181818	0.75	1
100	*logistic*	*adam*	0.727272727	0.7	1
100	*logistic*	*adam*	0.636363636	0.625	0.833333333
100	*logistic*	*adam*	0.727272727	0.8	0.888888889
100	*logistic*	*adam*	0.454545455	0.5	0.833333333
100	*logistic*	*lbfgs*	0.818181818	1	0.714285714
100	*logistic*	*lbfgs*	0.818181818	0.875	0.875
100	*logistic*	*lbfgs*	0.818181818	0.875	0.875
100	*logistic*	*lbfgs*	0.545454545	0.625	0.714285714
100	*logistic*	*lbfgs*	0.363636364	0.428571429	0.5
100	*logistic*	*lbfgs*	0.636363636	0.857142857	0.666666667
100	*logistic*	*lbfgs*	0.454545455	0.75	0.375
100	*logistic*	*lbfgs*	0.818181818	1	0.777777778
100	*logistic*	*lbfgs*	0.636363636	0.666666667	0.4
100	*logistic*	*lbfgs*	0.636363636	0.833333333	0.625
100	*logistic*	*lbfgs*	0.545454545	0.714285714	0.625
100	*logistic*	*lbfgs*	0.454545455	0.5	0.333333333
100	*logistic*	*lbfgs*	0.545454545	0.444444444	1
100	*logistic*	*lbfgs*	0.545454545	0.666666667	0.571428571
100	*logistic*	*lbfgs*	0.363636364	0.5	0.428571429
100	*logistic*	*lbfgs*	0.545454545	0.6	0.5

100	*logistic*	*lbfgs*	1	1	1
100	*logistic*	*lbfgs*	0.636363636	0.666666667	0.857142857
100	*logistic*	*lbfgs*	0.818181818	0.833333333	0.833333333
100	*logistic*	*lbfgs*	0.727272727	1	0.625
100	*logistic*	*lbfgs*	0.545454545	0.714285714	0.625
100	*logistic*	*lbfgs*	0.727272727	0.8	0.666666667
100	*logistic*	*lbfgs*	0.454545455	0.375	0.75
100	*logistic*	*lbfgs*	0.545454545	0.571428571	0.666666667
100	*logistic*	*lbfgs*	0.727272727	0.714285714	0.833333333
100	*logistic*	*lbfgs*	0.727272727	0.8	0.666666667
100	*logistic*	*lbfgs*	0.636363636	0.857142857	0.666666667
100	*logistic*	*lbfgs*	0.727272727	0.727272727	1
100	*logistic*	*lbfgs*	0.636363636	0.666666667	0.666666667
100	*logistic*	*lbfgs*	0.636363636	0.625	0.833333333
100	*logistic*	*lbfgs*	0.909090909	1	0.833333333
100	*logistic*	*lbfgs*	0.454545455	0.555555556	0.714285714
100	*logistic*	*lbfgs*	0.727272727	0.571428571	1
100	*logistic*	*lbfgs*	0.727272727	0.8	0.888888889
100	*logistic*	*lbfgs*	0.909090909	0.888888889	1
100	*logistic*	*lbfgs*	0.818181818	0.8	1
100	*logistic*	*lbfgs*	0.636363636	0.777777778	0.777777778
100	*logistic*	*lbfgs*	0.636363636	0.5	0.5
100	*logistic*	*lbfgs*	0.909090909	1	0.833333333
100	*logistic*	*lbfgs*	0.454545455	0.625	0.625
100	*logistic*	*lbfgs*	0.636363636	0.75	0.75
100	*logistic*	*lbfgs*	0.545454545	0.714285714	0.625
100	*logistic*	*lbfgs*	0.818181818	0.857142857	0.857142857
100	*logistic*	*lbfgs*	0.818181818	0.8	1
100	*logistic*	*lbfgs*	0.454545455	0.444444444	0.8
100	*logistic*	*lbfgs*	0.636363636	0.857142857	0.666666667
100	*logistic*	*lbfgs*	0.727272727	0.8	0.666666667
100	*logistic*	*lbfgs*	0.454545455	0.714285714	0.555555556
100	*logistic*	*lbfgs*	0.727272727	0.833333333	0.714285714
100	*logistic*	*lbfgs*	0.545454545	0.5	0.6
100	*logistic*	*sgd*	0.545454545	0.545454545	1
100	*logistic*	*sgd*	0.818181818	0.8	1
100	*logistic*	*sgd*	0.818181818	0.8	1
100	*logistic*	*sgd*	0.818181818	0.8	1
100	*logistic*	*sgd*	0.545454545	0.444444444	1
100	*logistic*	*sgd*	0.727272727	0.666666667	1
100	*logistic*	*sgd*	0.818181818	0.888888889	0.888888889
100	*logistic*	*sgd*	0.636363636	0.6	1
100	*logistic*	*sgd*	0.727272727	0.7	1

100	*logistic*	*sgd*	0.545454545	0.555555556	0.833333333
100	*logistic*	*sgd*	0.727272727	0.727272727	1
100	*logistic*	*sgd*	0.727272727	0.727272727	1
100	*logistic*	*sgd*	0.454545455	0.5	0.666666667
100	*logistic*	*sgd*	0.636363636	0.833333333	0.625
100	*logistic*	*sgd*	0.636363636	0.7	0.875
100	*logistic*	*sgd*	0.636363636	0.636363636	1
100	*logistic*	*sgd*	0.727272727	0.7	1
100	*logistic*	*sgd*	0.727272727	0.714285714	0.833333333
100	*logistic*	*sgd*	0.727272727	0.727272727	1
100	*logistic*	*sgd*	0.636363636	0.6	1
100	*logistic*	*sgd*	0.545454545	0.5	1
100	*logistic*	*sgd*	0.818181818	0.75	1
100	*logistic*	*sgd*	0.727272727	0.777777778	0.875
100	*logistic*	*sgd*	0.545454545	0.545454545	1
100	*logistic*	*sgd*	0.636363636	0.666666667	0.857142857
100	*logistic*	*sgd*	0.727272727	0.727272727	1
100	*logistic*	*sgd*	0.909090909	0.9	1
100	*logistic*	*sgd*	0.818181818	0.818181818	1
100	*logistic*	*sgd*	0.636363636	0.7	0.875
100	*logistic*	*sgd*	0.727272727	0.777777778	0.875
100	*logistic*	*sgd*	0.363636364	0.363636364	1
100	*logistic*	*sgd*	0.454545455	0.4	1
100	*logistic*	*sgd*	0.727272727	0.833333333	0.714285714
100	*logistic*	*sgd*	0.727272727	0.666666667	1
100	*logistic*	*sgd*	0.727272727	1	0.666666667
100	*logistic*	*sgd*	0.545454545	0.545454545	1
100	*logistic*	*sgd*	0.636363636	0.7	0.875
100	*logistic*	*sgd*	0.818181818	0.818181818	1
100	*logistic*	*sgd*	0.363636364	0.363636364	1
100	*logistic*	*sgd*	0.636363636	0.625	0.833333333
100	*logistic*	*sgd*	0.454545455	0.454545455	1
100	*logistic*	*sgd*	0.727272727	0.727272727	1
100	*logistic*	*sgd*	0.636363636	0.777777778	0.777777778
100	*logistic*	*sgd*	0.727272727	0.8	0.888888889
100	*logistic*	*sgd*	0.545454545	0.555555556	0.833333333
100	*logistic*	*sgd*	0.909090909	0.888888889	1
100	*logistic*	*sgd*	0.454545455	0.444444444	0.8
100	*logistic*	*sgd*	0.545454545	0.625	0.714285714
100	*logistic*	*sgd*	0.636363636	0.636363636	1
100	*logistic*	*sgd*	0.727272727	0.7	1
100	*relu*	*adam*	0.636363636	0.5	1
100	*relu*	*adam*	0.727272727	0.857142857	0.75

100	*relu*	*adam*	0.727272727	0.714285714	0.833333333
100	*relu*	*adam*	0.727272727	1	0.666666667
100	*relu*	*adam*	0.818181818	0.8	1
100	*relu*	*adam*	0.727272727	1	0.625
100	*relu*	*adam*	0.818181818	0.888888889	0.888888889
100	*relu*	*adam*	0.636363636	0.5	0.5
100	*relu*	*adam*	0.818181818	0.857142857	0.857142857
100	*relu*	*adam*	0.818181818	0.875	0.875
100	*relu*	*adam*	0.727272727	0.727272727	1
100	*relu*	*adam*	0.727272727	0.571428571	1
100	*relu*	*adam*	0.818181818	0.875	0.875
100	*relu*	*adam*	0.818181818	0.833333333	0.833333333
100	*relu*	*adam*	0.454545455	0.454545455	1
100	*relu*	*adam*	0.636363636	0.714285714	0.714285714
100	*relu*	*adam*	0.818181818	0.8	1
100	*relu*	*adam*	0.909090909	0.8	1
100	*relu*	*adam*	0.363636364	0.4	0.333333333
100	*relu*	*adam*	0.909090909	0.833333333	1
100	*relu*	*adam*	0.636363636	0.833333333	0.625
100	*relu*	*adam*	0.727272727	0.8	0.666666667
100	*relu*	*adam*	0.909090909	0.857142857	1
100	*relu*	*adam*	0.818181818	1	0.777777778
100	*relu*	*adam*	0.545454545	0.5	1
100	*relu*	*adam*	0.818181818	0.777777778	1
100	*relu*	*adam*	0.909090909	0.875	1
100	*relu*	*adam*	0.636363636	0.666666667	0.666666667
100	*relu*	*adam*	0.818181818	0.875	0.875
100	*relu*	*adam*	0.636363636	0.833333333	0.625
100	*relu*	*adam*	0.727272727	0.75	0.857142857
100	*relu*	*adam*	0.363636364	0.6	0.375
100	*relu*	*adam*	0.454545455	0.75	0.375
100	*relu*	*adam*	0.909090909	0.857142857	1
100	*relu*	*adam*	0.727272727	0.75	0.857142857
100	*relu*	*adam*	0.909090909	1	0.857142857
100	*relu*	*adam*	0.545454545	0.833333333	0.555555556
100	*relu*	*adam*	0.727272727	1	0.666666667
100	*relu*	*adam*	0.818181818	1	0.666666667
100	*relu*	*adam*	1	1	1
100	*relu*	*adam*	0.636363636	0.857142857	0.666666667
100	*relu*	*adam*	0.909090909	0.888888889	1
100	*relu*	*adam*	1	1	1
100	*relu*	*adam*	0.909090909	0.857142857	1
100	*relu*	*adam*	0.545454545	0.714285714	0.625

100	*relu*	*adam*	0.727272727	0.833333333	0.714285714
100	*relu*	*adam*	0.727272727	0.727272727	1
100	*relu*	*adam*	0.818181818	1	0.777777778
100	*relu*	*adam*	0.545454545	0.555555556	0.833333333
100	*relu*	*adam*	0.727272727	0.666666667	1
100	*relu*	*lbfgs*	0.727272727	0.571428571	1
100	*relu*	*lbfgs*	0.818181818	0.818181818	1
100	*relu*	*lbfgs*	0.636363636	0.714285714	0.714285714
100	*relu*	*lbfgs*	0.727272727	0.8	0.888888889
100	*relu*	*lbfgs*	0.818181818	0.875	0.875
100	*relu*	*lbfgs*	0.545454545	0.545454545	1
100	*relu*	*lbfgs*	1	1	1
100	*relu*	*lbfgs*	0.727272727	0.727272727	1
100	*relu*	*lbfgs*	0.727272727	0.857142857	0.75
100	*relu*	*lbfgs*	0.727272727	0.727272727	1
100	*relu*	*lbfgs*	1	1	1
100	*relu*	*lbfgs*	0.636363636	0.636363636	1
100	*relu*	*lbfgs*	0.727272727	0.857142857	0.75
100	*relu*	*lbfgs*	0.727272727	0.75	0.857142857
100	*relu*	*lbfgs*	0.818181818	0.75	1
100	*relu*	*lbfgs*	0.636363636	0.666666667	0.666666667
100	*relu*	*lbfgs*	0.818181818	0.8	1
100	*relu*	*lbfgs*	0.909090909	0.857142857	1
100	*relu*	*lbfgs*	1	1	1
100	*relu*	*lbfgs*	0.545454545	0.545454545	1
100	*relu*	*lbfgs*	0.545454545	0.545454545	1
100	*relu*	*lbfgs*	0.727272727	0.8	0.666666667
100	*relu*	*lbfgs*	0.636363636	0.5	0.75
100	*relu*	*lbfgs*	0.454545455	0.454545455	1
100	*relu*	*lbfgs*	0.727272727	0.727272727	1
100	*relu*	*lbfgs*	0.636363636	0.666666667	0.666666667
100	*relu*	*lbfgs*	0.727272727	0.727272727	1
100	*relu*	*lbfgs*	0.636363636	0.636363636	1
100	*relu*	*lbfgs*	0.545454545	0.6	0.857142857
100	*relu*	*lbfgs*	0.727272727	0.727272727	1
100	*relu*	*lbfgs*	0.636363636	0.636363636	1
100	*relu*	*lbfgs*	0.727272727	0.727272727	1
100	*relu*	*lbfgs*	1	1	1
100	*relu*	*lbfgs*	0.909090909	1	0.833333333
100	*relu*	*lbfgs*	0.727272727	0.875	0.777777778
100	*relu*	*lbfgs*	0.727272727	0.727272727	1
100	*relu*	*lbfgs*	0.727272727	0.875	0.777777778
100	*relu*	*lbfgs*	0.636363636	0.5	0.75

100	*relu*	*lbfgs*	0.636363636	0.666666667	0.666666667
100	*relu*	*lbfgs*	0.818181818	0.714285714	1
100	*relu*	*lbfgs*	0.727272727	0.727272727	1
100	*relu*	*lbfgs*	0.818181818	0.833333333	0.833333333
100	*relu*	*lbfgs*	0.818181818	0.857142857	0.857142857
100	*relu*	*lbfgs*	0.909090909	0.833333333	1
100	*relu*	*lbfgs*	0.909090909	1	0.875
100	*relu*	*lbfgs*	0.818181818	0.818181818	1
100	*relu*	*lbfgs*	0.727272727	0.857142857	0.75
100	*relu*	*lbfgs*	0.818181818	0.818181818	1
100	*relu*	*lbfgs*	0.545454545	0.545454545	1
100	*relu*	*lbfgs*	0.727272727	0.727272727	1
100	*relu*	*sgd*	0.454545455	0.5	0.833333333
100	*relu*	*sgd*	0.636363636	0.666666667	0.666666667
100	*relu*	*sgd*	0.818181818	0.8	1
100	*relu*	*sgd*	0.727272727	0.727272727	1
100	*relu*	*sgd*	0.727272727	0.714285714	0.833333333
100	*relu*	*sgd*	0.727272727	0.777777778	0.875
100	*relu*	*sgd*	0.727272727	0.666666667	1
100	*relu*	*sgd*	0.636363636	0.714285714	0.714285714
100	*relu*	*sgd*	0.727272727	0.875	0.777777778
100	*relu*	*sgd*	0.363636364	0.333333333	0.4
100	*relu*	*sgd*	0.545454545	0.714285714	0.625
100	*relu*	*sgd*	0.636363636	0.666666667	0.4
100	*relu*	*sgd*	0.818181818	0.777777778	1
100	*relu*	*sgd*	0.636363636	0.7	0.875
100	*relu*	*sgd*	1	1	1
100	*relu*	*sgd*	0.636363636	0.666666667	0.666666667
100	*relu*	*sgd*	0.545454545	0.6	0.857142857
100	*relu*	*sgd*	0.818181818	0.8	1
100	*relu*	*sgd*	0.636363636	0.625	0.833333333
100	*relu*	*sgd*	0.636363636	0.833333333	0.625
100	*relu*	*sgd*	0.727272727	0.727272727	1
100	*relu*	*sgd*	0.454545455	0.428571429	0.6
100	*relu*	*sgd*	0.727272727	0.714285714	0.833333333
100	*relu*	*sgd*	0.727272727	0.666666667	1
100	*relu*	*sgd*	0.636363636	0.857142857	0.666666667
100	*relu*	*sgd*	0.636363636	0.5	1
100	*relu*	*sgd*	0.545454545	1	0.166666667
100	*relu*	*sgd*	0.454545455	0.375	0.75
100	*relu*	*sgd*	0.545454545	0.4	0.5
100	*relu*	*sgd*	0.545454545	0.8	0.5
100	*relu*	*sgd*	0.636363636	0.7	0.875

100	*relu*	*sgd*	0.636363636	0.857142857	0.666666667
100	*relu*	*sgd*	0.545454545	0.714285714	0.625
100	*relu*	*sgd*	0.727272727	0.7	1
100	*relu*	*sgd*	0.545454545	0.6	0.857142857
100	*relu*	*sgd*	0.545454545	0.833333333	0.555555556
100	*relu*	*sgd*	0.727272727	0.875	0.777777778
100	*relu*	*sgd*	0.636363636	0.666666667	0.857142857
100	*relu*	*sgd*	0.727272727	0.777777778	0.875
100	*relu*	*sgd*	0.545454545	0.833333333	0.555555556
100	*relu*	*sgd*	0.272727273	0.3	0.75
100	*relu*	*sgd*	0.636363636	1	0.333333333
100	*relu*	*sgd*	0.818181818	0.8	1
100	*relu*	*sgd*	0.454545455	0.6	0.428571429
100	*relu*	*sgd*	0.636363636	0.625	0.833333333
100	*relu*	*sgd*	0.818181818	0.8	1
100	*relu*	*sgd*	0.454545455	0.5	0.5
100	*relu*	*sgd*	0.636363636	0.625	0.833333333
100	*relu*	*sgd*	0.909090909	0.9	1
100	*relu*	*sgd*	0.818181818	0.777777778	1
100	*tanh*	*adam*	0.818181818	0.833333333	0.833333333
100	*tanh*	*adam*	0.818181818	0.818181818	1
100	*tanh*	*adam*	0.636363636	0.75	0.75
100	*tanh*	*adam*	0.818181818	0.75	1
100	*tanh*	*adam*	0.636363636	0.857142857	0.666666667
100	*tanh*	*adam*	0.818181818	1	0.777777778
100	*tanh*	*adam*	0.818181818	0.833333333	0.833333333
100	*tanh*	*adam*	0.545454545	0.555555556	0.833333333
100	*tanh*	*adam*	0.636363636	0.777777778	0.777777778
100	*tanh*	*adam*	0.636363636	0.625	0.833333333
100	*tanh*	*adam*	0.636363636	0.7	0.875
100	*tanh*	*adam*	0.636363636	0.833333333	0.625
100	*tanh*	*adam*	0.727272727	0.714285714	0.833333333
100	*tanh*	*adam*	0.727272727	0.7	1
100	*tanh*	*adam*	0.727272727	0.777777778	0.875
100	*tanh*	*adam*	0.818181818	0.777777778	1
100	*tanh*	*adam*	0.636363636	0.6	1
100	*tanh*	*adam*	0.636363636	0.555555556	1
100	*tanh*	*adam*	0.727272727	0.7	1
100	*tanh*	*adam*	0.727272727	0.666666667	1
100	*tanh*	*adam*	0.818181818	1	0.777777778
100	*tanh*	*adam*	0.727272727	0.833333333	0.714285714
100	*tanh*	*adam*	0.636363636	0.625	0.833333333
100	*tanh*	*adam*	0.545454545	0.6	0.857142857

100	*tanh*	*adam*	0.727272727	0.777777778	0.875
100	*tanh*	*adam*	0.909090909	0.888888889	1
100	*tanh*	*adam*	0.727272727	0.777777778	0.875
100	*tanh*	*adam*	0.727272727	0.7	1
100	*tanh*	*adam*	0.636363636	0.6	0.6
100	*tanh*	*adam*	0.727272727	0.75	0.857142857
100	*tanh*	*adam*	0.727272727	0.714285714	0.833333333
100	*tanh*	*adam*	0.818181818	0.875	0.875
100	*tanh*	*adam*	0.636363636	0.5	1
100	*tanh*	*adam*	0.818181818	0.875	0.875
100	*tanh*	*adam*	0.818181818	0.875	0.875
100	*tanh*	*adam*	0.636363636	0.666666667	0.666666667
100	*tanh*	*adam*	0.636363636	0.571428571	0.8
100	*tanh*	*adam*	0.636363636	0.6	1
100	*tanh*	*adam*	0.636363636	0.5	1
100	*tanh*	*adam*	0.727272727	0.777777778	0.875
100	*tanh*	*adam*	0.636363636	0.75	0.75
100	*tanh*	*adam*	0.727272727	0.875	0.777777778
100	*tanh*	*adam*	0.636363636	0.7	0.875
100	*tanh*	*adam*	0.636363636	0.75	0.75
100	*tanh*	*adam*	0.818181818	0.75	1
100	*tanh*	*adam*	0.454545455	0.4	1
100	*tanh*	*adam*	0.909090909	0.888888889	1
100	*tanh*	*adam*	0.454545455	0.571428571	0.571428571
100	*tanh*	*adam*	0.363636364	0.363636364	1
100	*tanh*	*adam*	0.727272727	0.875	0.777777778
100	*tanh*	*lbfgs*	0.636363636	0.666666667	0.666666667
100	*tanh*	*lbfgs*	0.454545455	0.4	1
100	*tanh*	*lbfgs*	0.727272727	0.875	0.777777778
100	*tanh*	*lbfgs*	0.727272727	0.875	0.777777778
100	*tanh*	*lbfgs*	0.636363636	0.714285714	0.714285714
100	*tanh*	*lbfgs*	0.636363636	0.625	0.833333333
100	*tanh*	*lbfgs*	0.636363636	1	0.5
100	*tanh*	*lbfgs*	0.272727273	0.5	0.375
100	*tanh*	*lbfgs*	0.727272727	0.727272727	1
100	*tanh*	*lbfgs*	0.727272727	0.75	0.857142857
100	*tanh*	*lbfgs*	0.636363636	1	0.555555556
100	*tanh*	*lbfgs*	0.454545455	0.666666667	0.5
100	*tanh*	*lbfgs*	0.545454545	0.6	0.5
100	*tanh*	*lbfgs*	0.545454545	0.8	0.5
100	*tanh*	*lbfgs*	0.545454545	0.666666667	0.571428571
100	*tanh*	*lbfgs*	0.545454545	0.666666667	0.333333333
100	*tanh*	*lbfgs*	0.545454545	0.571428571	0.666666667

100	*tanh*	*lbfgs*	0.818181818	0.8	1
100	*tanh*	*lbfgs*	0.636363636	0.666666667	0.666666667
100	*tanh*	*lbfgs*	0.727272727	0.666666667	1
100	*tanh*	*lbfgs*	0.545454545	0.4	0.5
100	*tanh*	*lbfgs*	0.545454545	0.5	1
100	*tanh*	*lbfgs*	0.909090909	0.888888889	1
100	*tanh*	*lbfgs*	0.272727273	0.375	0.5
100	*tanh*	*lbfgs*	0.909090909	1	0.857142857
100	*tanh*	*lbfgs*	0.454545455	0.5	0.5
100	*tanh*	*lbfgs*	0.636363636	0.666666667	0.857142857
100	*tanh*	*lbfgs*	0.909090909	1	0.875
100	*tanh*	*lbfgs*	0.545454545	0.714285714	0.625
100	*tanh*	*lbfgs*	0.727272727	0.7	1
100	*tanh*	*lbfgs*	0.727272727	0.6	0.75
100	*tanh*	*lbfgs*	0.636363636	0.666666667	0.666666667
100	*tanh*	*lbfgs*	0.727272727	0.777777778	0.875
100	*tanh*	*lbfgs*	0.545454545	0.6	0.857142857
100	*tanh*	*lbfgs*	0.454545455	0.5	0.5
100	*tanh*	*lbfgs*	0.636363636	0.857142857	0.666666667
100	*tanh*	*lbfgs*	1	1	1
100	*tanh*	*lbfgs*	0.545454545	0.714285714	0.625
100	*tanh*	*lbfgs*	0.545454545	0.444444444	1
100	*tanh*	*lbfgs*	0.545454545	0.666666667	0.75
100	*tanh*	*lbfgs*	0.727272727	0.75	0.857142857
100	*tanh*	*lbfgs*	0.909090909	0.857142857	1
100	*tanh*	*lbfgs*	0.454545455	0.4	0.4
100	*tanh*	*lbfgs*	0.545454545	0.5	0.8
100	*tanh*	*lbfgs*	0.545454545	0.666666667	0.75
100	*tanh*	*lbfgs*	0.909090909	1	0.875
100	*tanh*	*lbfgs*	0.454545455	0.714285714	0.555555556
100	*tanh*	*lbfgs*	0.818181818	0.875	0.875
100	*tanh*	*lbfgs*	0.454545455	0.5	0.5
100	*tanh*	*lbfgs*	0.636363636	0.777777778	0.777777778
100	*tanh*	*sgd*	0.727272727	0.777777778	0.875
100	*tanh*	*sgd*	0.727272727	0.857142857	0.75
100	*tanh*	*sgd*	0.545454545	0.625	0.714285714
100	*tanh*	*sgd*	0.727272727	0.666666667	1
100	*tanh*	*sgd*	0.727272727	0.7	1
100	*tanh*	*sgd*	0.727272727	0.714285714	0.833333333
100	*tanh*	*sgd*	0.727272727	0.714285714	0.833333333
100	*tanh*	*sgd*	0.545454545	1	0.444444444
100	*tanh*	*sgd*	0.727272727	0.875	0.777777778
100	*tanh*	*sgd*	0.818181818	0.777777778	1

100	*tanh*	*sgd*	0.545454545	0.75	0.666666667
100	*tanh*	*sgd*	0.545454545	0.555555556	0.833333333
100	*tanh*	*sgd*	0.636363636	0.5	1
100	*tanh*	*sgd*	0.545454545	0.666666667	0.75
100	*tanh*	*sgd*	0.545454545	0.666666667	0.75
100	*tanh*	*sgd*	0.636363636	0.777777778	0.777777778
100	*tanh*	*sgd*	0.363636364	0.571428571	0.5
100	*tanh*	*sgd*	0.727272727	0.75	0.857142857
100	*tanh*	*sgd*	0.636363636	0.777777778	0.777777778
100	*tanh*	*sgd*	0.545454545	0.625	0.714285714
100	*tanh*	*sgd*	0.727272727	0.777777778	0.875
100	*tanh*	*sgd*	0.454545455	0.5	0.833333333
100	*tanh*	*sgd*	0.545454545	0.833333333	0.555555556
100	*tanh*	*sgd*	0.454545455	0.375	0.75
100	*tanh*	*sgd*	0.636363636	0.666666667	0.666666667
100	*tanh*	*sgd*	0.545454545	0.428571429	0.75
100	*tanh*	*sgd*	0.636363636	0.5	1
100	*tanh*	*sgd*	0.818181818	0.8	1
100	*tanh*	*sgd*	0.818181818	0.75	1
100	*tanh*	*sgd*	0.454545455	0.666666667	0.5
100	*tanh*	*sgd*	0.545454545	0.5	1
100	*tanh*	*sgd*	0.818181818	0.75	1
100	*tanh*	*sgd*	0.545454545	0.666666667	0.75
100	*tanh*	*sgd*	0.909090909	0.888888889	1
100	*tanh*	*sgd*	0.727272727	0.75	0.857142857
100	*tanh*	*sgd*	0.727272727	0.727272727	1
100	*tanh*	*sgd*	0.818181818	0.833333333	0.833333333
100	*tanh*	*sgd*	0.545454545	0.625	0.714285714
100	*tanh*	*sgd*	0.636363636	0.6	1
100	*tanh*	*sgd*	0.636363636	0.714285714	0.714285714
100	*tanh*	*sgd*	0.727272727	0.875	0.777777778
100	*tanh*	*sgd*	0.727272727	0.666666667	0.8
100	*tanh*	*sgd*	0.727272727	0.625	1
100	*tanh*	*sgd*	0.727272727	0.777777778	0.875
100	*tanh*	*sgd*	0.818181818	0.8	1
100	*tanh*	*sgd*	0.727272727	0.727272727	1
100	*tanh*	*sgd*	0.545454545	0.555555556	0.833333333
100	*tanh*	*sgd*	0.727272727	0.714285714	0.833333333
100	*tanh*	*sgd*	0.636363636	0.625	0.833333333
100	*tanh*	*sgd*	0.818181818	0.8	1
300	*identity*	*adam*	1	1	1
300	*identity*	*adam*	1	1	1
300	*identity*	*adam*	0.909090909	0.888888889	1

300	*identity*	*adam*	0.909090909	1	0.888888889
300	*identity*	*adam*	0.727272727	0.8	0.666666667
300	*identity*	*adam*	0.818181818	1	0.6
300	*identity*	*adam*	0.818181818	1	0.75
300	*identity*	*adam*	1	1	1
300	*identity*	*adam*	0.909090909	0.857142857	1
300	*identity*	*adam*	0.636363636	0.5	0.75
300	*identity*	*adam*	0.909090909	0.857142857	1
300	*identity*	*adam*	0.818181818	0.8	1
300	*identity*	*adam*	0.909090909	1	0.857142857
300	*identity*	*adam*	0.363636364	0.6	0.375
300	*identity*	*adam*	1	1	1
300	*identity*	*adam*	1	1	1
300	*identity*	*adam*	1	1	1
300	*identity*	*adam*	0.727272727	0.833333333	0.714285714
300	*identity*	*adam*	1	1	1
300	*identity*	*adam*	0.909090909	1	0.875
300	*identity*	*adam*	0.909090909	0.8	1
300	*identity*	*adam*	0.909090909	1	0.875
300	*identity*	*adam*	1	1	1
300	*identity*	*adam*	0.909090909	0.857142857	1
300	*identity*	*adam*	0.454545455	0.666666667	0.285714286
300	*identity*	*adam*	0.909090909	1	0.888888889
300	*identity*	*adam*	0.818181818	0.75	1
300	*identity*	*adam*	1	1	1
300	*identity*	*adam*	0.818181818	0.857142857	0.857142857
300	*identity*	*adam*	0.636363636	0.75	0.5
300	*identity*	*adam*	1	1	1
300	*identity*	*adam*	0.909090909	0.888888889	1
300	*identity*	*adam*	0.818181818	1	0.666666667
300	*identity*	*adam*	0.818181818	0.75	0.75
300	*identity*	*adam*	1	1	1
300	*identity*	*adam*	0.454545455	1	0.333333333
300	*identity*	*adam*	1	1	1
300	*identity*	*adam*	0.727272727	0.833333333	0.714285714
300	*identity*	*adam*	1	1	1
300	*identity*	*adam*	1	1	1
300	*identity*	*adam*	0.909090909	0.875	1
300	*identity*	*adam*	1	1	1
300	*identity*	*adam*	0.727272727	0.8	0.888888889
300	*identity*	*adam*	1	1	1
300	*identity*	*adam*	0.818181818	0.8	1
300	*identity*	*adam*	0.454545455	0.5	0.166666667

300	*identity*	*adam*	1	1	1
300	*identity*	*adam*	1	1	1
300	*identity*	*adam*	0.727272727	0.666666667	1
300	*identity*	*adam*	1	1	1
300	*identity*	*lbfgs*	0.909090909	1	0.875
300	*identity*	*lbfgs*	0.909090909	0.857142857	1
300	*identity*	*lbfgs*	0.545454545	0.545454545	1
300	*identity*	*lbfgs*	0.545454545	0.545454545	1
300	*identity*	*lbfgs*	0.818181818	0.857142857	0.857142857
300	*identity*	*lbfgs*	1	1	1
300	*identity*	*lbfgs*	0.909090909	1	0.888888889
300	*identity*	*lbfgs*	0.818181818	0.875	0.875
300	*identity*	*lbfgs*	0.909090909	0.888888889	1
300	*identity*	*lbfgs*	1	1	1
300	*identity*	*lbfgs*	0.909090909	0.8	1
300	*identity*	*lbfgs*	0.545454545	0.6	0.5
300	*identity*	*lbfgs*	1	1	1
300	*identity*	*lbfgs*	0.454545455	0.5	0.333333333
300	*identity*	*lbfgs*	0.909090909	1	0.888888889
300	*identity*	*lbfgs*	1	1	1
300	*identity*	*lbfgs*	1	1	1
300	*identity*	*lbfgs*	0.909090909	1	0.888888889
300	*identity*	*lbfgs*	0.545454545	0.545454545	1
300	*identity*	*lbfgs*	1	1	1
300	*identity*	*lbfgs*	0.909090909	1	0.857142857
300	*identity*	*lbfgs*	1	1	1
300	*identity*	*lbfgs*	0.909090909	0.888888889	1
300	*identity*	*lbfgs*	0.818181818	0.818181818	1
300	*identity*	*lbfgs*	1	1	1
300	*identity*	*lbfgs*	0.545454545	0.545454545	1
300	*identity*	*lbfgs*	0.909090909	0.888888889	1
300	*identity*	*lbfgs*	1	1	1
300	*identity*	*lbfgs*	0.727272727	0.727272727	1
300	*identity*	*lbfgs*	0.636363636	0.636363636	1
300	*identity*	*lbfgs*	0.818181818	0.818181818	1
300	*identity*	*lbfgs*	0.909090909	1	0.888888889
300	*identity*	*lbfgs*	0.727272727	0.777777778	0.875
300	*identity*	*lbfgs*	1	1	1
300	*identity*	*lbfgs*	1	1	1
300	*identity*	*lbfgs*	0.909090909	1	0.875
300	*identity*	*lbfgs*	0.636363636	0.636363636	1
300	*identity*	*lbfgs*	1	1	1
300	*identity*	*lbfgs*	0.545454545	0.555555556	0.833333333

300	*identity*	*lbfgs*	1	1	1
300	*identity*	*lbfgs*	0.909090909	0.888888889	1
300	*identity*	*lbfgs*	1	1	1
300	*identity*	*lbfgs*	0.909090909	0.875	1
300	*identity*	*lbfgs*	0.727272727	0.727272727	1
300	*identity*	*lbfgs*	0.545454545	0.545454545	1
300	*identity*	*lbfgs*	0.545454545	0.545454545	1
300	*identity*	*lbfgs*	1	1	1
300	*identity*	*lbfgs*	0.636363636	0.636363636	1
300	*identity*	*lbfgs*	0.818181818	0.75	0.75
300	*identity*	*lbfgs*	0.636363636	0.636363636	1
300	*identity*	*sgd*	0.454545455	0.714285714	0.555555556
300	*identity*	*sgd*	0.636363636	0.857142857	0.666666667
300	*identity*	*sgd*	0.636363636	1	0.5
300	*identity*	*sgd*	0.636363636	1	0.5
300	*identity*	*sgd*	0.454545455	0.25	0.25
300	*identity*	*sgd*	0.727272727	0.7	1
300	*identity*	*sgd*	0.454545455	0.333333333	0.5
300	*identity*	*sgd*	0.545454545	0.555555556	0.833333333
300	*identity*	*sgd*	0.454545455	0.8	0.444444444
300	*identity*	*sgd*	0.454545455	0.5	0.666666667
300	*identity*	*sgd*	0.454545455	0.625	0.625
300	*identity*	*sgd*	0.727272727	0.8	0.888888889
300	*identity*	*sgd*	0.363636364	0.363636364	1
300	*identity*	*sgd*	0.545454545	0.571428571	0.666666667
300	*identity*	*sgd*	0.272727273	0.2	0.2
300	*identity*	*sgd*	0.636363636	0.777777778	0.777777778
300	*identity*	*sgd*	0.636363636	0.7	0.875
300	*identity*	*sgd*	0.909090909	0.9	1
300	*identity*	*sgd*	0.454545455	0	0
300	*identity*	*sgd*	0.909090909	1	0.875
300	*identity*	*sgd*	0.909090909	1	0.8
300	*identity*	*sgd*	0.636363636	0.666666667	0.857142857
300	*identity*	*sgd*	0.272727273	0.428571429	0.428571429
300	*identity*	*sgd*	0.545454545	0.666666667	0.571428571
300	*identity*	*sgd*	0.545454545	0.545454545	1
300	*identity*	*sgd*	0.545454545	1	0.375
300	*identity*	*sgd*	0.727272727	0.857142857	0.75
300	*identity*	*sgd*	0.818181818	1	0.75
300	*identity*	*sgd*	0.454545455	0.333333333	0.5
300	*identity*	*sgd*	0.818181818	0.75	1
300	*identity*	*sgd*	0.636363636	0.625	0.833333333
300	*identity*	*sgd*	0.636363636	0.857142857	0.666666667

300	*identity*	*sgd*	0.636363636	0.666666667	0.857142857
300	*identity*	*sgd*	0.454545455	0.625	0.625
300	*identity*	*sgd*	0.454545455	0.5	0.333333333
300	*identity*	*sgd*	0.454545455	0.5	0.666666667
300	*identity*	*sgd*	0.727272727	0.777777778	0.875
300	*identity*	*sgd*	0.727272727	0.857142857	0.75
300	*identity*	*sgd*	0.727272727	0.777777778	0.875
300	*identity*	*sgd*	0.545454545	0.75	0.428571429
300	*identity*	*sgd*	0.727272727	0.7	1
300	*identity*	*sgd*	0.636363636	0.625	0.833333333
300	*identity*	*sgd*	0.545454545	0.545454545	1
300	*identity*	*sgd*	0.636363636	0.7	0.875
300	*identity*	*sgd*	0.545454545	0.555555556	0.833333333
300	*identity*	*sgd*	0.454545455	0.454545455	1
300	*identity*	*sgd*	0.727272727	0.777777778	0.875
300	*identity*	*sgd*	0.818181818	1	0.666666667
300	*identity*	*sgd*	0.454545455	0.625	0.625
300	*identity*	*sgd*	0.454545455	0.666666667	0.285714286
300	*logistic*	*adam*	0.818181818	0.875	0.875
300	*logistic*	*adam*	0.909090909	0.888888889	1
300	*logistic*	*adam*	0.818181818	0.666666667	1
300	*logistic*	*adam*	0.727272727	0.7	1
300	*logistic*	*adam*	1	1	1
300	*logistic*	*adam*	1	1	1
300	*logistic*	*adam*	0.636363636	0.625	0.833333333
300	*logistic*	*adam*	0.727272727	0.833333333	0.714285714
300	*logistic*	*adam*	0.909090909	0.888888889	1
300	*logistic*	*adam*	0.909090909	0.888888889	1
300	*logistic*	*adam*	0.909090909	0.888888889	1
300	*logistic*	*adam*	0.727272727	0.75	0.857142857
300	*logistic*	*adam*	0.818181818	0.75	1
300	*logistic*	*adam*	0.818181818	0.75	1
300	*logistic*	*adam*	0.818181818	0.666666667	1
300	*logistic*	*adam*	0.818181818	0.8	1
300	*logistic*	*adam*	0.727272727	0.727272727	1
300	*logistic*	*adam*	1	1	1
300	*logistic*	*adam*	0.727272727	0.777777778	0.875
300	*logistic*	*adam*	0.909090909	0.888888889	1
300	*logistic*	*adam*	0.818181818	1	0.75
300	*logistic*	*adam*	0.909090909	0.857142857	1
300	*logistic*	*adam*	0.909090909	0.833333333	1
300	*logistic*	*adam*	0.818181818	0.818181818	1
300	*logistic*	*adam*	0.909090909	0.888888889	1

300	*logistic*	*adam*	0.818181818	1	0.777777778
300	*logistic*	*adam*	0.727272727	0.714285714	0.833333333
300	*logistic*	*adam*	1	1	1
300	*logistic*	*adam*	0.909090909	0.857142857	1
300	*logistic*	*adam*	0.818181818	0.888888889	0.888888889
300	*logistic*	*adam*	1	1	1
300	*logistic*	*adam*	1	1	1
300	*logistic*	*adam*	0.727272727	0.7	1
300	*logistic*	*adam*	0.818181818	0.875	0.875
300	*logistic*	*adam*	0.818181818	0.8	0.8
300	*logistic*	*adam*	0.818181818	1	0.777777778
300	*logistic*	*adam*	1	1	1
300	*logistic*	*adam*	0.727272727	0.571428571	1
300	*logistic*	*adam*	0.909090909	0.857142857	1
300	*logistic*	*adam*	0.818181818	0.8	1
300	*logistic*	*adam*	1	1	1
300	*logistic*	*adam*	0.818181818	0.777777778	1
300	*logistic*	*adam*	1	1	1
300	*logistic*	*adam*	0.727272727	0.875	0.777777778
300	*logistic*	*adam*	0.909090909	1	0.833333333
300	*logistic*	*adam*	0.818181818	0.777777778	1
300	*logistic*	*adam*	0.818181818	0.75	1
300	*logistic*	*adam*	0.727272727	0.8	0.888888889
300	*logistic*	*adam*	0.727272727	0.625	1
300	*logistic*	*adam*	0.636363636	0.666666667	0.666666667
300	*logistic*	*lbfgs*	0.727272727	0.8	0.666666667
300	*logistic*	*lbfgs*	0.636363636	0.625	0.833333333
300	*logistic*	*lbfgs*	0.636363636	0.75	0.75
300	*logistic*	*lbfgs*	0.727272727	0.8	0.888888889
300	*logistic*	*lbfgs*	0.636363636	0.75	0.75
300	*logistic*	*lbfgs*	0.636363636	0.5	0.75
300	*logistic*	*lbfgs*	0.727272727	0.7	1
300	*logistic*	*lbfgs*	0.818181818	0.75	1
300	*logistic*	*lbfgs*	0.727272727	0.8	0.666666667
300	*logistic*	*lbfgs*	0.636363636	0.5	0.75
300	*logistic*	*lbfgs*	0.636363636	0.833333333	0.625
300	*logistic*	*lbfgs*	0.636363636	0.857142857	0.666666667
300	*logistic*	*lbfgs*	0.727272727	0.714285714	0.833333333
300	*logistic*	*lbfgs*	0.818181818	1	0.666666667
300	*logistic*	*lbfgs*	0.454545455	0.5	0.5
300	*logistic*	*lbfgs*	0.454545455	0.5	0.333333333
300	*logistic*	*lbfgs*	0.727272727	0.666666667	0.8
300	*logistic*	*lbfgs*	0.818181818	0.888888889	0.888888889

300	*logistic*	*lbfgs*	0.545454545	0.714285714	0.625
300	*logistic*	*lbfgs*	0.636363636	0.571428571	0.8
300	*logistic*	*lbfgs*	0.727272727	0.875	0.777777778
300	*logistic*	*lbfgs*	0.545454545	0.666666667	0.571428571
300	*logistic*	*lbfgs*	0.454545455	0.625	0.625
300	*logistic*	*lbfgs*	0.545454545	0.6	0.5
300	*logistic*	*lbfgs*	0.727272727	0.6	0.75
300	*logistic*	*lbfgs*	0.636363636	0.75	0.75
300	*logistic*	*lbfgs*	0.727272727	0.8	0.888888889
300	*logistic*	*lbfgs*	0.818181818	0.857142857	0.857142857
300	*logistic*	*lbfgs*	0.727272727	0.777777778	0.875
300	*logistic*	*lbfgs*	0.636363636	0.833333333	0.625
300	*logistic*	*lbfgs*	0.818181818	1	0.777777778
300	*logistic*	*lbfgs*	0.454545455	0.75	0.375
300	*logistic*	*lbfgs*	0.818181818	1	0.777777778
300	*logistic*	*lbfgs*	0.454545455	0.666666667	0.5
300	*logistic*	*lbfgs*	0.727272727	0.75	0.6
300	*logistic*	*lbfgs*	0.818181818	0.875	0.875
300	*logistic*	*lbfgs*	0.545454545	0.8	0.5
300	*logistic*	*lbfgs*	0.909090909	0.875	1
300	*logistic*	*lbfgs*	0.545454545	0.555555556	0.833333333
300	*logistic*	*lbfgs*	0.454545455	0.571428571	0.571428571
300	*logistic*	*lbfgs*	0.909090909	1	0.875
300	*logistic*	*lbfgs*	0.545454545	0.6	0.5
300	*logistic*	*lbfgs*	0.909090909	1	0.875
300	*logistic*	*lbfgs*	0.636363636	0.666666667	0.666666667
300	*logistic*	*lbfgs*	0.545454545	0.625	0.714285714
300	*logistic*	*lbfgs*	0.545454545	0.6	0.5
300	*logistic*	*lbfgs*	0.636363636	0.833333333	0.625

Table S10. Measures of quality in the models created by using the Support vector machines tool during assessment for classification of sheep farms based on prevalence of subclinical mastitis.

Regularisation parametre	Kernel	Accuracy	Precision	Recall
1	*linear*	1	1	1
1	*linear*	0.727272727	0.875	0.777777778
1	*linear*	0.909090909	1	0.857142857
1	*linear*	1	1	1
1	*linear*	0.909090909	1	0.833333333
1	*linear*	0.909090909	0.888888889	1
1	*linear*	1	1	1
1	*linear*	1	1	1
1	*linear*	1	1	1
1	*linear*	0.909090909	0.875	1
1	*linear*	0.909090909	0.875	1
1	*linear*	0.909090909	0.888888889	1
1	*linear*	1	1	1
1	*linear*	0.818181818	0.888888889	0.888888889
1	*linear*	0.909090909	0.888888889	1
1	*linear*	1	1	1
1	*linear*	1	1	1
1	*linear*	1	1	1
1	*linear*	0.909090909	1	0.875
1	*linear*	1	1	1
1	*linear*	1	1	1
1	*linear*	0.909090909	0.8	1
1	*linear*	1	1	1
1	*linear*	0.727272727	0.6	0.75
1	*linear*	0.909090909	1	0.888888889
1	*linear*	1	1	1
1	*linear*	0.909090909	1	0.888888889
1	*linear*	1	1	1
1	*linear*	1	1	1
1	*linear*	0.909090909	0.833333333	1
1	*linear*	0.909090909	0.888888889	1
1	*linear*	1	1	1
1	*linear*	0.909090909	0.857142857	1
1	*linear*	0.909090909	1	0.875
1	*linear*	0.909090909	1	0.888888889
1	*linear*	0.909090909	1	0.857142857
1	*linear*	0.909090909	0.857142857	1
1	*linear*	0.818181818	0.875	0.875

1	*linear*	0.909090909	0.888888889	1
1	*linear*	0.909090909	1	0.833333333
1	*linear*	1	1	1
1	*linear*	0.909090909	1	0.857142857
1	*linear*	1	1	1
1	*linear*	0.909090909	0.857142857	1
1	*linear*	1	1	1
1	*linear*	0.909090909	0.888888889	1
1	*linear*	0.909090909	0.875	1
1	*linear*	0.909090909	0.875	1
1	*linear*	1	1	1
1	*linear*	1	1	1
1	*poly*	0.545454545	0.545454545	1
1	*poly*	0.818181818	0.875	0.875
1	*poly*	0.727272727	0.727272727	1
1	*poly*	0.545454545	0.6	0.857142857
1	*poly*	0.818181818	0.818181818	1
1	*poly*	0.636363636	0.636363636	1
1	*poly*	0.545454545	0.545454545	1
1	*poly*	0.727272727	0.727272727	1
1	*poly*	0.818181818	0.818181818	1
1	*poly*	0.636363636	0.666666667	0.857142857
1	*poly*	0.818181818	0.8	1
1	*poly*	0.363636364	0.4	0.8
1	*poly*	0.272727273	0.333333333	0.6
1	*poly*	0.818181818	0.818181818	1
1	*poly*	0.727272727	0.727272727	1
1	*poly*	0.636363636	0.636363636	1
1	*poly*	0.545454545	0.571428571	0.666666667
1	*poly*	0.636363636	0.636363636	1
1	*poly*	0.545454545	0.6	0.857142857
1	*poly*	0.636363636	0.636363636	1
1	*poly*	0.545454545	0.545454545	1
1	*poly*	0.727272727	0.727272727	1
1	*poly*	0.727272727	0.727272727	1
1	*poly*	0.363636364	0.363636364	1
1	*poly*	0.727272727	0.727272727	1
1	*poly*	0.454545455	0.454545455	1
1	*poly*	0.454545455	0.5	0.833333333
1	*poly*	0.818181818	0.818181818	1
1	*poly*	0.363636364	0.363636364	1
1	*poly*	0.727272727	0.666666667	1
1	*poly*	0.545454545	0.6	0.857142857

1	*poly*	0.636363636	0.7	0.875
1	*poly*	0.727272727	0.727272727	1
1	*poly*	0.454545455	0.5	0.833333333
1	*poly*	0.545454545	0.545454545	1
1	*poly*	0.727272727	0.8	0.888888889
1	*poly*	0.727272727	0.727272727	1
1	*poly*	0.636363636	0.75	0.75
1	*poly*	0.545454545	0.555555556	0.833333333
1	*poly*	0.818181818	0.8	1
1	*poly*	0.454545455	0.5	0.833333333
1	*poly*	0.545454545	0.545454545	1
1	*poly*	0.727272727	0.727272727	1
1	*poly*	0.363636364	0.363636364	1
1	*poly*	0.636363636	0.7	0.875
1	*poly*	0.363636364	0.363636364	1
1	*poly*	0.909090909	0.9	1
1	*poly*	0.818181818	0.888888889	0.888888889
1	*poly*	0.454545455	0.5	0.833333333
1	*poly*	0.545454545	0.545454545	1
1	*rbf*	0.727272727	0.727272727	1
1	*rbf*	0.636363636	0.636363636	1
1	*rbf*	0.636363636	0.636363636	1
1	*rbf*	0.636363636	0.636363636	1
1	*rbf*	0.818181818	0.818181818	1
1	*rbf*	0.545454545	0.545454545	1
1	*rbf*	0.727272727	0.727272727	1
1	*rbf*	0.545454545	0.545454545	1
1	*rbf*	0.636363636	0.636363636	1
1	*rbf*	0.727272727	0.727272727	1
1	*rbf*	0.545454545	0.545454545	1
1	*rbf*	0.727272727	0.727272727	1
1	*rbf*	0.545454545	0.545454545	1
1	*rbf*	0.545454545	0.545454545	1
1	*rbf*	0.545454545	0.545454545	1
1	*rbf*	0.727272727	0.727272727	1
1	*rbf*	0.727272727	0.727272727	1
1	*rbf*	0.636363636	0.636363636	1
1	*rbf*	0.636363636	0.636363636	1
1	*rbf*	0.727272727	0.727272727	1
1	*rbf*	0.727272727	0.727272727	1
1	*rbf*	0.818181818	0.818181818	1
1	*rbf*	0.818181818	0.818181818	1
1	*rbf*	0.727272727	0.727272727	1

1	*rbf*	0.545454545	0.545454545	1
1	*rbf*	0.727272727	0.727272727	1
1	*rbf*	0.727272727	0.727272727	1
1	*rbf*	0.545454545	0.545454545	1
1	*rbf*	0.727272727	0.727272727	1
1	*rbf*	0.818181818	0.818181818	1
1	*rbf*	0.818181818	0.818181818	1
1	*rbf*	0.545454545	0.545454545	1
1	*rbf*	0.636363636	0.7	0.875
1	*rbf*	0.727272727	0.727272727	1
1	*rbf*	0.454545455	0.454545455	1
1	*rbf*	0.636363636	0.636363636	1
1	*rbf*	0.363636364	0.363636364	1
1	*rbf*	0.545454545	0.545454545	1
1	*rbf*	0.818181818	0.818181818	1
1	*rbf*	0.545454545	0.545454545	1
1	*rbf*	0.818181818	0.818181818	1
1	*rbf*	0.363636364	0.363636364	1
1	*rbf*	0.363636364	0.363636364	1
1	*rbf*	0.636363636	0.636363636	1
1	*rbf*	0.545454545	0.545454545	1
1	*rbf*	0.454545455	0.454545455	1
1	*rbf*	0.727272727	0.727272727	1
1	*rbf*	0.363636364	0.363636364	1
1	*rbf*	0.545454545	0.545454545	1
1	*rbf*	0.454545455	0.454545455	1
1	*sigmoid*	0.636363636	0.636363636	1
1	*sigmoid*	0.818181818	0.8	1
1	*sigmoid*	0.727272727	0.727272727	1
1	*sigmoid*	0.727272727	0.727272727	1
1	*sigmoid*	0.545454545	0.545454545	1
1	*sigmoid*	0.363636364	0.363636364	1
1	*sigmoid*	0.454545455	0.454545455	1
1	*sigmoid*	0.636363636	0.636363636	1
1	*sigmoid*	0.545454545	0.545454545	1
1	*sigmoid*	0.545454545	0.545454545	1
1	*sigmoid*	0.454545455	0.454545455	1
1	*sigmoid*	0.818181818	0.818181818	1
1	*sigmoid*	0.545454545	0.545454545	1
1	*sigmoid*	0.818181818	0.818181818	1
1	*sigmoid*	0.727272727	0.727272727	1
1	*sigmoid*	0.545454545	0.545454545	1
1	*sigmoid*	0.545454545	0.545454545	1

1	*sigmoid*	0.727272727	0.727272727	1
1	*sigmoid*	0.727272727	0.727272727	1
1	*sigmoid*	0.727272727	0.727272727	1
1	*sigmoid*	0.454545455	0.454545455	1
1	*sigmoid*	0.545454545	0.545454545	1
1	*sigmoid*	0.545454545	0.545454545	1
1	*sigmoid*	0.636363636	0.636363636	1
1	*sigmoid*	0.545454545	0.545454545	1
1	*sigmoid*	0.636363636	0.636363636	1
1	*sigmoid*	0.636363636	0.636363636	1
1	*sigmoid*	0.363636364	0.363636364	1
1	*sigmoid*	0.545454545	0.545454545	1
1	*sigmoid*	0.727272727	0.727272727	1
1	*sigmoid*	0.727272727	0.727272727	1
1	*sigmoid*	0.727272727	0.727272727	1
1	*sigmoid*	0.636363636	0.636363636	1
1	*sigmoid*	0.545454545	0.545454545	1
1	*sigmoid*	0.818181818	0.818181818	1
1	*sigmoid*	0.727272727	0.727272727	1
1	*sigmoid*	0.636363636	0.636363636	1
1	*sigmoid*	0.727272727	0.727272727	1
1	*sigmoid*	0.818181818	0.818181818	1
1	*sigmoid*	0.818181818	0.818181818	1
1	*sigmoid*	0.727272727	0.727272727	1
1	*sigmoid*	0.363636364	0.363636364	1
1	*sigmoid*	0.727272727	0.727272727	1
1	*sigmoid*	0.727272727	0.727272727	1
1	*sigmoid*	0.363636364	0.363636364	1
1	*sigmoid*	0.818181818	0.818181818	1
1	*sigmoid*	0.545454545	0.545454545	1
1	*sigmoid*	0.636363636	0.636363636	1
1	*sigmoid*	0.545454545	0.545454545	1
1	*sigmoid*	0.818181818	0.818181818	1
2	*linear*	1	1	1
2	*linear*	1	1	1
2	*linear*	0.909090909	0.875	1
2	*linear*	0.909090909	0.875	1
2	*linear*	0.909090909	1	0.833333333
2	*linear*	0.909090909	0.888888889	1
2	*linear*	0.909090909	1	0.857142857
2	*linear*	1	1	1
2	*linear*	0.909090909	0.857142857	1
2	*linear*	0.818181818	0.875	0.875

2	*linear*	1	1	1
2	*linear*	0.909090909	0.888888889	1
2	*linear*	1	1	1
2	*linear*	0.909090909	0.857142857	1
2	*linear*	0.909090909	0.888888889	1
2	*linear*	1	1	1
2	*linear*	1	1	1
2	*linear*	1	1	1
2	*linear*	1	1	1
2	*linear*	1	1	1
2	*linear*	0.909090909	1	0.888888889
2	*linear*	0.909090909	1	0.857142857
2	*linear*	0.909090909	0.857142857	1
2	*linear*	0.909090909	1	0.875
2	*linear*	1	1	1
2	*linear*	0.909090909	0.8	1
2	*linear*	1	1	1
2	*linear*	1	1	1
2	*linear*	1	1	1
2	*linear*	0.909090909	1	0.875
2	*linear*	0.909090909	1	0.888888889
2	*linear*	1	1	1
2	*linear*	1	1	1
2	*linear*	0.727272727	0.6	0.75
2	*linear*	0.909090909	0.875	1
2	*linear*	1	1	1
2	*linear*	0.909090909	0.888888889	1
2	*linear*	0.909090909	0.875	1
2	*linear*	1	1	1
2	*linear*	1	1	1
2	*linear*	0.909090909	0.888888889	1
2	*linear*	1	1	1
2	*linear*	1	1	1
2	*linear*	0.818181818	0.888888889	0.888888889
2	*linear*	1	1	1
2	*linear*	1	1	1
2	*linear*	0.909090909	1	0.833333333
2	*linear*	1	1	1
2	*linear*	0.818181818	0.857142857	0.857142857
2	*linear*	0.727272727	0.875	0.777777778
2	*poly*	0.545454545	0.545454545	1
2	*poly*	0.727272727	0.8	0.888888889
2	*poly*	0.727272727	0.727272727	1

2	*poly*	0.636363636	0.666666667	0.857142857
2	*poly*	0.727272727	0.777777778	0.875
2	*poly*	0.636363636	0.625	0.833333333
2	*poly*	0.818181818	0.8	1
2	*poly*	0.818181818	0.818181818	1
2	*poly*	0.363636364	0.363636364	1
2	*poly*	0.727272727	0.666666667	1
2	*poly*	0.818181818	0.8	1
2	*poly*	0.545454545	0.5	1
2	*poly*	0.545454545	0.555555556	0.833333333
2	*poly*	0.909090909	0.9	1
2	*poly*	0.818181818	0.888888889	0.888888889
2	*poly*	0.454545455	0.5	0.833333333
2	*poly*	0.636363636	0.6	1
2	*poly*	0.636363636	0.7	0.875
2	*poly*	0.545454545	0.444444444	1
2	*poly*	0.636363636	0.6	1
2	*poly*	0.818181818	0.8	1
2	*poly*	0.363636364	0.363636364	1
2	*poly*	0.727272727	0.777777778	0.875
2	*poly*	0.454545455	0.5	0.666666667
2	*poly*	0.818181818	0.8	1
2	*poly*	0.545454545	0.555555556	0.833333333
2	*poly*	0.636363636	0.666666667	0.857142857
2	*poly*	0.818181818	0.8	1
2	*poly*	0.363636364	0.4	0.8
2	*poly*	0.545454545	0.545454545	1
2	*poly*	0.727272727	0.727272727	1
2	*poly*	0.818181818	0.818181818	1
2	*poly*	0.545454545	0.6	0.857142857
2	*poly*	0.818181818	0.818181818	1
2	*poly*	0.727272727	0.7	1
2	*poly*	0.727272727	0.666666667	1
2	*poly*	0.818181818	0.875	0.875
2	*poly*	0.727272727	0.727272727	1
2	*poly*	0.818181818	0.8	1
2	*poly*	0.454545455	0.4	1
2	*poly*	0.545454545	0.6	0.857142857
2	*poly*	0.636363636	0.666666667	0.857142857
2	*poly*	0.545454545	0.545454545	1
2	*poly*	0.727272727	0.727272727	1
2	*poly*	0.818181818	0.8	1
2	*poly*	0.636363636	0.636363636	1

2	*poly*	0.545454545	0.571428571	0.666666667
2	*poly*	0.727272727	0.7	1
2	*poly*	0.454545455	0.428571429	0.6
2	*poly*	0.818181818	0.818181818	1
2	*rbf*	0.636363636	0.636363636	1
2	*rbf*	0.818181818	0.8	1
2	*rbf*	0.636363636	0.636363636	1
2	*rbf*	0.727272727	0.727272727	1
2	*rbf*	0.545454545	0.545454545	1
2	*rbf*	0.545454545	0.545454545	1
2	*rbf*	0.727272727	0.727272727	1
2	*rbf*	0.727272727	0.727272727	1
2	*rbf*	0.545454545	0.545454545	1
2	*rbf*	0.727272727	0.727272727	1
2	*rbf*	0.727272727	0.777777778	0.875
2	*rbf*	0.818181818	0.818181818	1
2	*rbf*	0.818181818	0.818181818	1
2	*rbf*	0.727272727	0.8	0.888888889
2	*rbf*	0.636363636	0.636363636	1
2	*rbf*	0.545454545	0.545454545	1
2	*rbf*	0.727272727	0.727272727	1
2	*rbf*	0.636363636	0.636363636	1
2	*rbf*	0.727272727	0.727272727	1
2	*rbf*	0.636363636	0.636363636	1
2	*rbf*	0.727272727	0.727272727	1
2	*rbf*	0.545454545	0.545454545	1
2	*rbf*	0.545454545	0.545454545	1
2	*rbf*	0.454545455	0.5	0.833333333
2	*rbf*	0.727272727	0.727272727	1
2	*rbf*	0.636363636	0.666666667	0.857142857
2	*rbf*	0.363636364	0.363636364	1
2	*rbf*	0.636363636	0.636363636	1
2	*rbf*	0.818181818	0.888888889	0.888888889
2	*rbf*	0.545454545	0.545454545	1
2	*rbf*	0.363636364	0.363636364	1
2	*rbf*	0.818181818	0.888888889	0.888888889
2	*rbf*	0.545454545	0.545454545	1
2	*rbf*	0.363636364	0.363636364	1
2	*rbf*	0.454545455	0.454545455	1
2	*rbf*	0.363636364	0.4	0.8
2	*rbf*	0.545454545	0.545454545	1
2	*rbf*	0.727272727	0.727272727	1
2	*rbf*	0.727272727	0.8	0.888888889

2	*rbf*	0.545454545	0.545454545	1
2	*rbf*	0.727272727	0.727272727	1
2	*rbf*	0.545454545	0.555555556	0.833333333
2	*rbf*	0.818181818	0.818181818	1
2	*rbf*	0.636363636	0.7	0.875
2	*rbf*	0.363636364	0.363636364	1
2	*rbf*	0.636363636	0.636363636	1
2	*rbf*	0.636363636	0.6	1
2	*rbf*	0.818181818	0.8	1
2	*rbf*	0.636363636	0.7	0.875
2	*rbf*	0.454545455	0.454545455	1
2	*sigmoid*	0.545454545	0.545454545	1
2	*sigmoid*	0.636363636	0.636363636	1
2	*sigmoid*	0.454545455	0.5	0.833333333
2	*sigmoid*	0.454545455	0.454545455	1
2	*sigmoid*	0.727272727	0.727272727	1
2	*sigmoid*	0.545454545	0.545454545	1
2	*sigmoid*	0.545454545	0.545454545	1
2	*sigmoid*	0.727272727	0.727272727	1
2	*sigmoid*	0.727272727	0.727272727	1
2	*sigmoid*	0.818181818	0.818181818	1
2	*sigmoid*	0.545454545	0.545454545	1
2	*sigmoid*	0.727272727	0.727272727	1
2	*sigmoid*	0.818181818	0.818181818	1
2	*sigmoid*	0.454545455	0.454545455	1
2	*sigmoid*	0.545454545	0.545454545	1
2	*sigmoid*	0.545454545	0.545454545	1
2	*sigmoid*	0.545454545	0.545454545	1
2	*sigmoid*	0.636363636	0.636363636	1
2	*sigmoid*	0.454545455	0.454545455	1
2	*sigmoid*	0.363636364	0.333333333	0.75
2	*sigmoid*	0.545454545	0.545454545	1
2	*sigmoid*	0.727272727	0.727272727	1
2	*sigmoid*	0.818181818	0.8	1
2	*sigmoid*	0.818181818	0.8	1
2	*sigmoid*	0.636363636	0.636363636	1
2	*sigmoid*	0.818181818	0.818181818	1
2	*sigmoid*	0.545454545	0.545454545	1
2	*sigmoid*	0.636363636	0.636363636	1
2	*sigmoid*	0.545454545	0.545454545	1
2	*sigmoid*	0.818181818	0.818181818	1
2	*sigmoid*	0.363636364	0.363636364	1
2	*sigmoid*	0.727272727	0.727272727	1

2	*sigmoid*	0.818181818	0.8	1
2	*sigmoid*	0.818181818	0.818181818	1
2	*sigmoid*	0.363636364	0.363636364	1
2	*sigmoid*	0.727272727	0.727272727	1
2	*sigmoid*	0.727272727	0.727272727	1
2	*sigmoid*	0.818181818	0.818181818	1
2	*sigmoid*	0.636363636	0.636363636	1
2	*sigmoid*	0.727272727	0.727272727	1
2	*sigmoid*	0.818181818	0.818181818	1
2	*sigmoid*	0.545454545	0.6	0.857142857
2	*sigmoid*	0.545454545	0.545454545	1
2	*sigmoid*	0.727272727	0.727272727	1
2	*sigmoid*	0.818181818	0.8	1
2	*sigmoid*	0.727272727	0.727272727	1
2	*sigmoid*	0.636363636	0.636363636	1
2	*sigmoid*	0.545454545	0.545454545	1
2	*sigmoid*	0.363636364	0.363636364	1
2	*sigmoid*	0.636363636	0.636363636	1
3	*linear*	1	1	1
3	*linear*	0.909090909	0.875	1
3	*linear*	0.909090909	0.875	1
3	*linear*	0.909090909	0.888888889	1
3	*linear*	1	1	1
3	*linear*	1	1	1
3	*linear*	0.909090909	0.888888889	1
3	*linear*	1	1	1
3	*linear*	1	1	1
3	*linear*	0.818181818	0.888888889	0.888888889
3	*linear*	1	1	1
3	*linear*	1	1	1
3	*linear*	1	1	1
3	*linear*	1	1	1
3	*linear*	0.909090909	1	0.875
3	*linear*	1	1	1
3	*linear*	0.909090909	1	0.888888889
3	*linear*	1	1	1
3	*linear*	1	1	1
3	*linear*	0.727272727	0.6	0.75
3	*linear*	1	1	1
3	*linear*	0.818181818	0.857142857	0.857142857
3	*linear*	0.727272727	0.875	0.777777778
3	*linear*	1	1	1
3	*linear*	1	1	1

3	*linear*	0.909090909	1	0.833333333
3	*linear*	0.909090909	0.875	1
3	*linear*	1	1	1
3	*linear*	1	1	1
3	*linear*	1	1	1
3	*linear*	1	1	1
3	*linear*	1	1	1
3	*linear*	1	1	1
3	*linear*	1	1	1
3	*linear*	1	1	1
3	*linear*	1	1	1
3	*linear*	0.909090909	1	0.888888889
3	*linear*	0.909090909	1	0.857142857
3	*linear*	0.909090909	0.857142857	1
3	*linear*	0.909090909	1	0.875
3	*linear*	0.909090909	0.888888889	1
3	*linear*	0.909090909	1	0.833333333
3	*linear*	1	1	1
3	*linear*	0.909090909	1	0.857142857
3	*linear*	0.909090909	0.857142857	1
3	*linear*	0.818181818	0.875	0.875
3	*linear*	1	1	1
3	*linear*	0.909090909	0.888888889	1
3	*linear*	1	1	1
3	*linear*	1	1	1
3	*poly*	0.545454545	0.545454545	1
3	*poly*	0.727272727	0.8	0.888888889
3	*poly*	0.727272727	0.727272727	1
3	*poly*	0.545454545	0.6	0.857142857
3	*poly*	0.727272727	0.777777778	0.875
3	*poly*	0.636363636	0.625	0.833333333
3	*poly*	0.818181818	0.8	1
3	*poly*	0.818181818	0.818181818	1
3	*poly*	0.454545455	0.4	1
3	*poly*	0.636363636	0.625	0.833333333
3	*poly*	0.818181818	0.8	1
3	*poly*	0.545454545	0.5	1
3	*poly*	0.545454545	0.555555556	0.833333333
3	*poly*	0.909090909	0.9	1
3	*poly*	0.818181818	0.888888889	0.888888889
3	*poly*	0.545454545	0.555555556	0.833333333
3	*poly*	0.727272727	0.666666667	1
3	*poly*	0.636363636	0.7	0.875

3	*poly*	0.545454545	0.444444444	1
3	*poly*	0.636363636	0.6	1
3	*poly*	0.818181818	0.8	1
3	*poly*	0.363636364	0.363636364	1
3	*poly*	0.727272727	0.777777778	0.875
3	*poly*	0.454545455	0.5	0.666666667
3	*poly*	0.818181818	0.8	1
3	*poly*	0.636363636	0.6	1
3	*poly*	0.636363636	0.666666667	0.857142857
3	*poly*	0.818181818	0.8	1
3	*poly*	0.454545455	0.444444444	0.8
3	*poly*	0.545454545	0.545454545	1
3	*poly*	0.727272727	0.727272727	1
3	*poly*	0.818181818	0.818181818	1
3	*poly*	0.545454545	0.6	0.857142857
3	*poly*	0.818181818	0.818181818	1
3	*poly*	0.727272727	0.7	1
3	*poly*	0.727272727	0.666666667	1
3	*poly*	0.818181818	0.875	0.875
3	*poly*	0.727272727	0.727272727	1
3	*poly*	0.818181818	0.8	1
3	*poly*	0.454545455	0.4	1
3	*poly*	0.545454545	0.6	0.857142857
3	*poly*	0.727272727	0.75	0.857142857
3	*poly*	0.545454545	0.545454545	1
3	*poly*	0.727272727	0.727272727	1
3	*poly*	0.818181818	0.8	1
3	*poly*	0.636363636	0.636363636	1
3	*poly*	0.545454545	0.571428571	0.666666667
3	*poly*	0.727272727	0.75	0.857142857
3	*poly*	0.454545455	0.428571429	0.6
3	*poly*	0.818181818	0.818181818	1
3	*rbf*	0.727272727	0.727272727	1
3	*rbf*	0.818181818	0.818181818	1
3	*rbf*	0.818181818	0.875	0.875
3	*rbf*	0.818181818	0.818181818	1
3	*rbf*	0.727272727	0.727272727	1
3	*rbf*	0.818181818	0.75	1
3	*rbf*	0.636363636	0.6	1
3	*rbf*	0.545454545	0.545454545	1
3	*rbf*	0.727272727	0.727272727	1
3	*rbf*	0.818181818	0.8	1
3	*rbf*	0.727272727	0.7	1

3	*rbf*	0.818181818	0.8	1
3	*rbf*	0.636363636	0.666666667	0.857142857
3	*rbf*	0.545454545	0.545454545	1
3	*rbf*	0.454545455	0.5	0.833333333
3	*rbf*	0.636363636	0.666666667	0.857142857
3	*rbf*	0.727272727	0.777777778	0.875
3	*rbf*	0.818181818	0.8	1
3	*rbf*	0.545454545	0.545454545	1
3	*rbf*	0.727272727	0.7	1
3	*rbf*	0.818181818	0.8	1
3	*rbf*	0.818181818	0.777777778	1
3	*rbf*	0.545454545	0.6	0.857142857
3	*rbf*	0.909090909	1	0.888888889
3	*rbf*	0.727272727	0.727272727	1
3	*rbf*	0.636363636	0.6	1
3	*rbf*	0.272727273	0.333333333	0.6
3	*rbf*	0.545454545	0.545454545	1
3	*rbf*	0.818181818	0.8	1
3	*rbf*	0.636363636	0.6	1
3	*rbf*	0.454545455	0.4	1
3	*rbf*	0.454545455	0.454545455	1
3	*rbf*	0.545454545	0.545454545	1
3	*rbf*	0.818181818	0.888888889	0.888888889
3	*rbf*	0.363636364	0.363636364	1
3	*rbf*	0.818181818	0.888888889	0.888888889
3	*rbf*	0.363636364	0.363636364	1
3	*rbf*	0.545454545	0.6	0.857142857
3	*rbf*	0.818181818	0.8	1
3	*rbf*	0.636363636	0.7	0.875
3	*rbf*	0.545454545	0.5	1
3	*rbf*	0.363636364	0.363636364	1
3	*rbf*	0.727272727	0.7	1
3	*rbf*	0.727272727	0.714285714	0.833333333
3	*rbf*	0.454545455	0.5	0.666666667
3	*rbf*	0.727272727	0.727272727	1
3	*rbf*	0.909090909	0.9	1
3	*rbf*	0.727272727	0.777777778	0.875
3	*rbf*	0.727272727	0.8	0.888888889
3	*rbf*	0.545454545	0.545454545	1
3	*sigmoid*	0.727272727	0.777777778	0.875
3	*sigmoid*	0.363636364	0.363636364	1
3	*sigmoid*	0.727272727	0.727272727	1
3	*sigmoid*	0.727272727	0.8	0.888888889

3	*sigmoid*	0.636363636	0.636363636	1
3	*sigmoid*	0.454545455	0.5	0.666666667
3	*sigmoid*	0.818181818	0.888888889	0.888888889
3	*sigmoid*	0.636363636	0.6	1
3	*sigmoid*	0.363636364	0.333333333	0.75
3	*sigmoid*	0.545454545	0.555555556	0.833333333
3	*sigmoid*	0.545454545	0.625	0.714285714
3	*sigmoid*	0.636363636	0.666666667	0.857142857
3	*sigmoid*	0.727272727	0.727272727	1
3	*sigmoid*	0.727272727	0.777777778	0.875
3	*sigmoid*	0.818181818	0.875	0.875
3	*sigmoid*	0.636363636	0.6	1
3	*sigmoid*	0.636363636	0.714285714	0.714285714
3	*sigmoid*	0.727272727	0.8	0.888888889
3	*sigmoid*	0.727272727	0.727272727	1
3	*sigmoid*	0.545454545	0.6	0.857142857
3	*sigmoid*	0.727272727	0.8	0.888888889
3	*sigmoid*	0.636363636	0.7	0.875
3	*sigmoid*	0.636363636	0.7	0.875
3	*sigmoid*	0.454545455	0.4	1
3	*sigmoid*	0.727272727	0.8	0.888888889
3	*sigmoid*	0.727272727	0.666666667	1
3	*sigmoid*	0.545454545	0.545454545	1
3	*sigmoid*	0.454545455	0.5	0.833333333
3	*sigmoid*	0.272727273	0.333333333	0.6
3	*sigmoid*	0.818181818	0.888888889	0.888888889
3	*sigmoid*	0.727272727	0.727272727	1
3	*sigmoid*	0.545454545	0.545454545	1
3	*sigmoid*	0.818181818	0.818181818	1
3	*sigmoid*	0.727272727	0.777777778	0.875
3	*sigmoid*	0.636363636	0.7	0.875
3	*sigmoid*	0.545454545	0.545454545	1
3	*sigmoid*	0.454545455	0.5	0.833333333
3	*sigmoid*	0.454545455	0.5	0.833333333
3	*sigmoid*	0.818181818	0.8	1
3	*sigmoid*	0.545454545	0.5	1
3	*sigmoid*	0.545454545	0.6	0.857142857
3	*sigmoid*	0.545454545	0.571428571	0.666666667
3	*sigmoid*	0.545454545	0.6	0.857142857
3	*sigmoid*	0.727272727	0.777777778	0.875
3	*sigmoid*	0.727272727	0.777777778	0.875
3	*sigmoid*	0.727272727	0.666666667	1
3	*sigmoid*	0.545454545	0.666666667	0.75

3	*sigmoid*	0.454545455	0.454545455	1
3	*sigmoid*	0.454545455	0.375	0.75
3	*sigmoid*	0.545454545	0.6	0.857142857
